# Cervimycin-Resistant Staphylococcus aureus Strains Display Vancomycin-Intermediate Resistant Phenotypes

**DOI:** 10.1128/spectrum.02567-22

**Published:** 2022-09-29

**Authors:** Alina Dietrich, Ursula Steffens, Mike Gajdiss, Anna-Lena Boschert, Jana Katharina Dröge, Christiane Szekat, Peter Sass, Imran T. Malik, Jan Bornikoel, Laura Reinke, Boris Maček, Mirita Franz-Wachtel, Kay Nieselt, Theresa Harbig, Kirstin Scherlach, Heike Brötz-Oesterhelt, Christian Hertweck, Hans-Georg Sahl, Gabriele Bierbaum

**Affiliations:** a University Hospital Bonn, Institute of Medical Microbiology, Immunology and Parasitology, Bonn, Germany; b University of Tübingen, Interfaculty Institute of Microbiology and Infection Medicine, Deptartment of Microbial Bioactive Compounds, Tübingen, Germany; c University of Tübingen, Proteome Center Tuebingen, Tübingen, Germany; d University of Tübingen, Interfaculty Institute for Bioinformatics and Medical Informatics, Tübingen, Germany; e Leibniz Institute for Natural Product Research and Infection Biology – Hans Knöll Institute (HKI), Jena, Germany; f Friedrich Schiller University Jena, Institute of Microbiology, Faculty of Biological Sciences, Jena, Germany; g University of Bonn, Institute for Pharmaceutical Microbiology, Bonn, Germany; Northwestern University

**Keywords:** ClpC, ClpP, DnaK, TCS, WalK/WalR, antibiotic resistance, naphthoquinone, vancomycin, vancomycin-intermediate resistant *S. aureus* (VISA)

## Abstract

Resistance to antibiotics is an increasing problem and necessitates novel antibacterial therapies. The polyketide antibiotics cervimycin A to D are natural products of Streptomyces tendae HKI 0179 with promising activity against multidrug-resistant staphylococci and vancomycin-resistant enterococci. To initiate mode of action studies, we selected cervimycin C- and D-resistant (CmR) Staphylococcus aureus strains. Genome sequencing of CmR mutants revealed amino acid exchanges in the essential histidine kinase WalK, the Clp protease proteolytic subunit ClpP or the Clp ATPase ClpC, and the heat shock protein DnaK. Interestingly, all characterized CmR mutants harbored a combination of mutations in *walK* and *clpP* or *clpC*. *In vitro* and *in vivo* analyses showed that the mutations in the Clp proteins abolished ClpP or ClpC activity, and the deletion of *clpP* rendered S. aureus but not all Bacillus subtilis strains cervimycin-resistant. The essential gene *walK* was the second mutational hotspot in the CmR S. aureus strains, which decreased WalK activity *in vitro* and generated a vancomycin-intermediate resistant phenotype, with a thickened cell wall, a lower growth rate, and reduced cell lysis. Transcriptomic and proteomic analyses revealed massive alterations in the CmR strains compared to the parent strain S. aureus SG511, with major shifts in the heat shock regulon, the metal ion homeostasis, and the carbohydrate metabolism. Taken together, mutations in the heat shock genes *clpP*, *clpC*, and *dnaK*, and the *walK* kinase gene in CmR mutants induced a vancomycin-intermediate resistant phenotype in S. aureus, suggesting cell wall metabolism or the Clp protease system as primary target of cervimycin.

**IMPORTANCE** Staphylococcus aureus is a frequent cause of infections in both the community and hospital setting. Resistance development of S. aureus to various antibiotics is a severe problem for the treatment of this pathogen worldwide. New powerful antimicrobial agents against Gram-positives are needed, since antibiotics like vancomycin fail to cure vancomycin-intermediate resistant S. aureus (VISA) and vancomycin-resistant enterococci (VRE) infections. One candidate substance with promising activity against these organisms is cervimycin, which is an antibiotic complex with a yet unknown mode of action. In our study, we provide first insights into the mode of action of cervimycins. By characterizing cervimycin-resistant S. aureus strains, we revealed the Clp system and the essential kinase WalK as mutational hotspots for cervimycin resistance in S. aureus. It further emerged that cervimycin-resistant S. aureus strains show a VISA phenotype, indicating a role of cervimycin in perturbing the bacterial cell envelope.

## INTRODUCTION

Gram-positive cocci like coagulase-negative staphylococci, Staphylococcus aureus, and *Enterococcus* spp., are important pathogens in the hospital environment (1). S. aureus is a leading cause of bacterial infections and mortality (2). Although the number of serious infections due to resistant strains has decreased in recent years (2), methicillin-resistant S. aureus (MRSA) remains a major health care issue (3). Therefore, new antibacterial agents are needed, preferably with novel modes of action to overcome resistance. *Streptomyces* species are a promising source of secondary metabolites, especially polyketides, with diverse biological activity (4).

**FIG 1:**
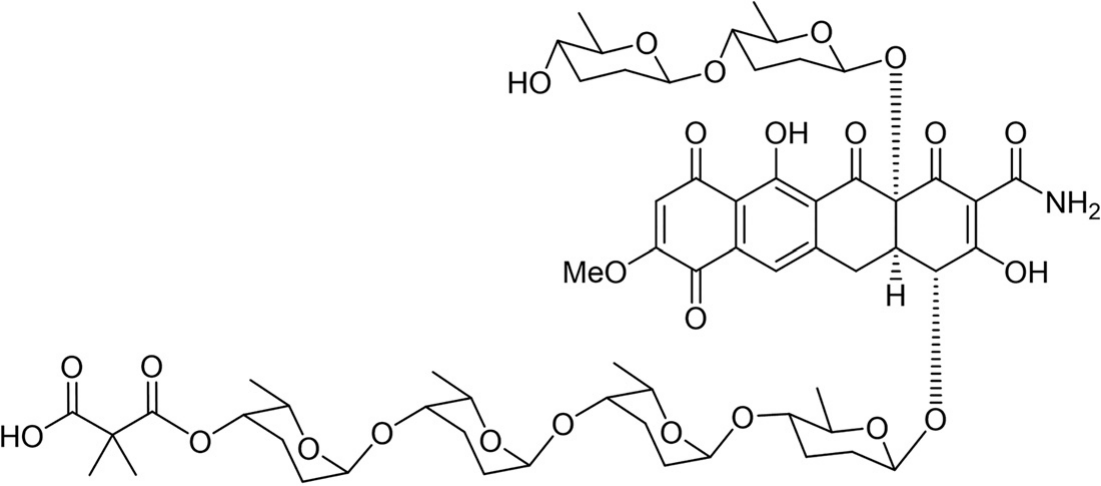
Structure of cervimycin C; relative configuration. Cervimycins are bi-glycosylated polyketides, decorated with unusual di- and tetrasaccharide chains. Cervimycin C is ring-substituted with a carbamoyl moiety and bears a dimethylmalonyl residue attached to the longer sugar side chain.

Cervimycins belong to this group and the substances were named after the location of their first discovery, the cave *Grotta dei Cervi* in Italy. The cervimycin antibiotic complex consists of four main components A to D, and the minor components E to K, produced by the actinomycete Streptomyces tendae HKI 0179 (5–7). Interestingly, the color of cervimycin is pH-dependent, yellow or violet, a typical property of naphthoquinoid systems that form phenolate salts under alkaline conditions (6). The structures of cervimycins are fully resolved (8) (Fig. 1); cervimycins are bi-glycosylated polyketides, ring-substituted with either a carbamoyl or an acetyl moiety and bear either a dimethylmalonyl or a monomethylmalonyl residue attached to the longer sugar side chain. The unusual di- and tetrasaccharide chains, composed of the trideoxysugars β-d-amicetose and α-l-rhodinose are pharmacophore groups of the molecules, connected via a 1,4-O-glycosidic linkage. The common structural feature of these molecules is the quinoid system on ring D of the naphthacene core, which is inverted relative to classical tetracyclines (8). Tetracyclines, that bear some structural resemblance, are inhibitors of protein biosynthesis by preventing the attachment of aminoacyl-tRNA to the ribosomal acceptor site (9). However, the cervimycins did not inhibit protein biosynthesis in a ribosome target test assay, indicating that the mode of action of these antibiotics is different (6).

Earlier precursor incorporation tests rather indicated the DNA metabolism as a possible target, but only at high concentrations (8× MIC) and with a considerable delay (35 min), whereas cell wall biosynthesis was not affected (6). Furthermore, a selection for cervimycin-resistant Bacillus subtilis mutants revealed an efflux-mediated resistance mechanism, but did not unveil the target structure of the antibiotic complex (10).

To close this gap, we investigated single nucleotide polymorphisms (SNPs) found in cervimycin-resistant S. aureus strains and the cervimycin resistance mechanisms. To this end, cervimycin-resistant S. aureus strains were generated by a serial passaging experiment. Interestingly, the transporter inhibitor reserpine only marginally affected cervimycin susceptibility in these strains, indicating an efflux-independent resistance mechanism. Genome sequencing of the cervimycin-resistant (CmR) strains revealed mutations in the nonessential caseinolytic protease gene *clpP* and its cognate ATPase gene *clpC*, and in the essential histidine kinase gene *walK*, which is part of the cell wall regulatory two-component system WalRK. Moreover, in one strain, an additional mutation in the nonessential heat shock protein 70 (HSP70) gene *dnaK* was observed. Importantly, the combination of *walK* and *clpP* or *clpC* mutations simultaneously increased the cervimycin and the vancomycin MICs of S. aureus, indicating the ClpCP complex or the Gram-positive cell envelope as possible targets of this novel antibiotic.

## RESULTS

### Cervimycin C is active against MRSA and VRE.

Extended testing of cervimycin C (CmC) and cervimycin D (CmD) confirmed earlier reports on the activity of cervimycin against MRSA and VRE (5) (Table 1). Analysis of the minimal bactericidal concentrations (MBCs) validated the bactericidal activity of CmC and CmD, with MBCs of one or two titer steps above the MIC values (Table S1). In contrast, mycobacteria and the Gram-negative bacteria Neisseria sicca and E. coli were hardly affected by the cervimycins. Even an E. coli strain with a defective outer membrane (E. coli MB5746) was not susceptible to cervimycin. The susceptibility of E. coli MB5746 was also not increased by the addition of polymyxin B nonapeptide (PMBN), which induces outer membrane permeability in Gram-negative bacteria (11). In contrast, E. coli MB5746 was further sensitized toward erythromycin (2-fold) and novobiocin (4-fold) by the addition of PMBN. Of note, the activity of cervimycin turned out to be pH-responsive, as the alkalization of the medium abolished the antibacterial activity (Table 2). This loss of antibacterial activity was accompanied by a color change from yellow to red-violet.

**TABLE 1:** MIC values (μg/mL) for cervimycin C (CmC) and D (CmD)^*a*^

Strain	CmC	CmD
Gram-positive		
Bacillus subtilis 168	0.25	0.5
Corynebacterium xerosis VA 167198	0.125	4
Enterococcus faecium BM 4147-1 (vancomycin-susceptible)	2	16
E. faecium BM 4147 (vancomycin-resistant)	2	4
Listeria welshimeri DSM 20650	0.25	4
Micrococcus luteus ATCC4698	0.5	1
Mycobacterium smegmatis	32	32
Staphylococcus aureus SG511 Berlin (methicillin-susceptible)	2	32
S. aureus N315 (methicillin-resistant)	4	64
Staphylococcus haemolyticus 655-2 (vancomycin-susceptible)	1	64
*S. haemolyticus* 655-2 R16 (vancomycin-resistant)	1	64
*S. haemolyticus* 655-2 R32 (vancomycin-resistant)	1	64
Staphylococcus simulans 22	8	64
Streptococcus agalactiae B Ku	0.5	0.25
Streptococcus pyogenes Ku	0.25	4
Gram-negative		
Escherichia coli MB5746 (*lpxC*, *tolC*:Tn*10*)	>64	>64
E. coli MB5746 (*lpxC*, *tolC*:Tn*10*) + 4 μg/mL PMBN^*b*^	>64	ND
Neisseria sicca Ku	64	64

a Broth dilution method was used in Müller Hinton medium and samples were incubated for 24 h.

*^b^*Polymyxin B nonapeptide (PMBN) is a derivative of polymyxin B and induces outer membrane permeability in Gram-negative bacteria.

**TABLE 2:** MIC values (μg/mL) of S. aureus SG511 Berlin for cervimycin C (CmC) at different pH values^*a*^

pH	MIC (μg/mL)
5	0.5
5.5	2
6	4
6.5	16
7	32
7.5	64
8	>64
8.5	>64

a Broth dilution method was used in Mäller Hinton medium with indicated pH values and samples were incubated for 24 h.

Also, as shown in Table 1, the methicillin-susceptible S. aureus SG511 Berlin was very susceptible toward CmC, but less susceptible toward CmD, which was generally less active than CmC. Therefore, all further tests were conducted with CmC, the major component of the antibiotic complex, unless stated otherwise. The mode of action of CmC is unknown. In B. subtilis firefly luciferase reporter strains (12) only the *yorB* promoter was activated, indicating a LexA-dependent DNA stress response. *yorB* encodes a protein of unknown function, that belongs to the LexA regulon of *Bacillus* and is strongly induced by substances, which produce double-strand breaks, while noncovalent DNA binding agents yield no signal induction. However, the induction level of the *yorB* promoter by CmC did not reach significance, remaining below a 2.5-fold increase above the baseline level (Fig. S1). Notably, the cell envelope stress reporters (*ypuA* and *liaI*) and the translation stalling reporter (*bmrC*) were not stimulated by cervimycin (Fig. S1).

### Whole-genome sequencing of cervimycin-resistant S. aureus strains reveals mutations in *walK* and *clpCP*.

To further explore, how cervimycin acts on bacterial cells, we generated cervimycin-resistant mutants by serial passaging of S. aureus SG511 with gradually increasing concentrations of CmC or CmD. In total, six cervimycin C-resistant (CmR-01 to CmR-03, resulting from 11 passages, and CmR-04 to CmR-06, from another 13 passages) and three cervimycin D-resistant S. aureus strains (CmR-07 to CmR-09, 22 passages) were isolated. Revertants (REV) were obtained by passaging of the CmR strains for several generations in nonselective medium (Fig. S2).

**FIG 2:**
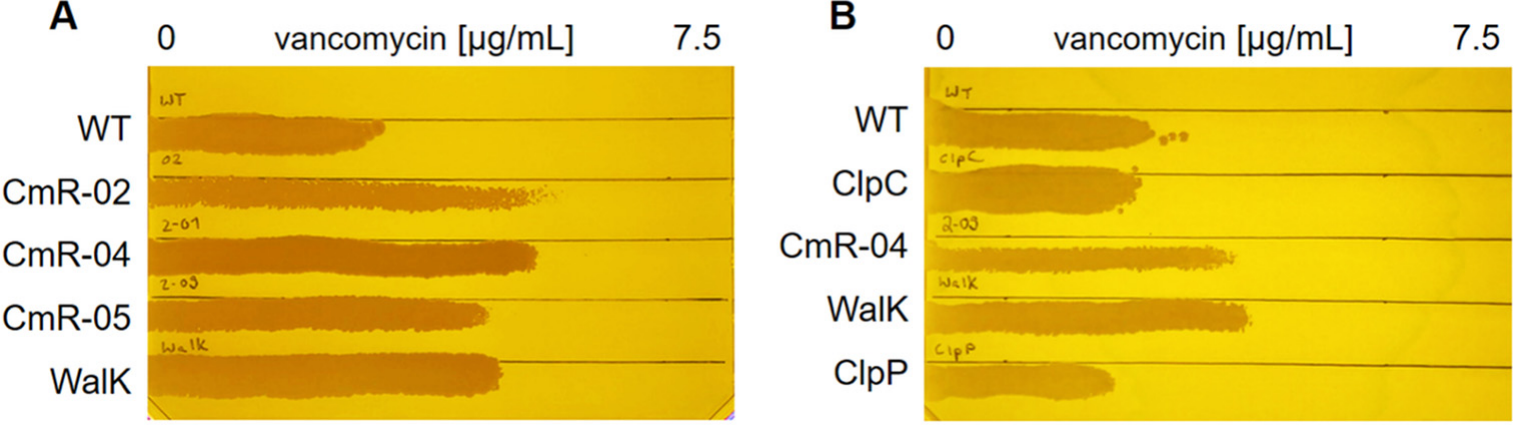
Gradient plates with increasing concentrations of vancomycin and 1.25 mM CaCl_2_. The CmR strains showed a decreased susceptibility toward vancomycin (A), which probably relied on the *walK* mutations in these strains, because the *walK* mutation alone decreased the vancomycin susceptibility (B). WT, S. aureus SG511; WalK, S. aureus WalK^A243V^; ClpC, S. aureus SG511 ClpC^T215I^; ClpP, S. aureus SG511 ClpP^I29F^.

Sequencing of resistant mutants and their revertants showed that all CmR strains harbored a combination of mutations in the essential histidine kinase gene *walK*, and in the genes encoding the nonessential Clp system, either in *clpP* or in *clpC* (Table 3 and Table S4). Only one strain (CmR-02) harbored an additional mutation in the heat shock protein gene *dnaK*. To investigate the effects of the single mutations and their possible participation in the resistance mechanism, SNPs were introduced into the genome of the S. aureus SG511 Berlin parent strain by markerless allelic replacement. S. aureus SG511 WalK^A243V^ was generated by reverting the *clpP* mutation in S. aureus CmR-01. Susceptibility testing toward commonly used antibiotics revealed a cross-resistance toward the lipopeptide daptomycin, and a reduced susceptibility of the CmR strains toward the glycopeptide antibiotics vancomycin and teicoplanin (Table 4, Fig. 2), but no further resistances toward other clinically used antibiotics, including tetracycline. In contrast, CmR-01, CmR-02, and CmR-03 were resistant toward the ClpP activating acyldepsipeptides (ADEPs). ADEPs bind to ClpP, the proteolytic core of the ClpP protease, leading to uncontrolled proteolysis, inhibition of bacterial cell division, and eventually cell death (13). As expected, the amino acid exchange in ClpP in CmR-02, which is also present in CmR-01 and CmR-03, or the deletion of the entire *clpP* (14), but not the mutations in the *clpC* gene (CmR-04 to CmR09) led to ADEP 4 resistance in S. aureus (Table 4). This suggested that the detected *clpP* mutations affected either the activity of ClpP or the binding of ADEPs to their target.

**TABLE 3:** Comparative whole-genome sequencing of S. aureus SG511 Berlin, the cervimycin resistant mutants (CmR), and the respective cervimycin-susceptible revertants (REV)^*a*^

Strain	MIC CmC	*clpP*	ClpP	*walK*	WalK
CmR-02^*b*^	128	A →T	I29F	C →T	A243V
02REV	2	A →T; G → C	I29F; M31I	C →T; C →T	S191L; A243V
CmR-03	128	A →T	I29F	C →T	A243V
03REV	2	A →G	I29V	C →T; C →T	S191L; A243V
	MIC CmC	*clpC*	ClpC	*walK*	WalK
CmR-04	128	4,561 bp Del.	Deletion	ΔCAA	ΔQ371
04REV	32	4,561 bp Del.	Deletion	ΔCAA; G →A	ΔQ371, A554T
CmR-05	128	C →T	T215I	C →T	A243V
05REV	16	C →T	T215I	C →T; C →T	A243V, T217M
	MIC CmD	*clpC*	ClpC	*walK*	WalK
CmR-09	107.5	C →A	P204H	T → G	Y549D
09REV	16	C →A	P204H	T → G; G →A	Y549D, R555H

*^a^*Sequence comparisons revealed a combination of amino acid exchanges in the protease ClpP or the cognate Clp ATPase ClpC, and the essential histidine kinase WalK.

b CmR-02 harbors an additional mutation in the heat shock protein gene *dnaK* (G → C), leading to the amino acid exchange A112P.

**TABLE 4:** MIC values of S. aureus strains in Müller Hinton medium or brain heart infusion broth^*a*^

S. aureus strain	Tet	VA	DAP	Teico	ADEP 4
SG511	0.5	1	0.25	0.5	0.25–0.5
CmR-02	0.0625	**4**	**2**	**8**	ND
CmR-04	0.25	**4**	**1**	**4**	ND
CmR-05	0.25	**4**	**1**	**4**	ND
CmR-09	0.25	**2**	**1**	**2**	ND
02REV	0.25	1	0.25	0.5	ND
04REV	0.25	1	0.125	0.5	ND
05REV	0.25	1	0.25	0.25	ND
09REV	0.25	**2**	0.25	**2**	ND
WalK^A243V^	0.5	**4**	**1**	**4**	0.5
ClpP^I29F^	0.125	1	0.5	1	**16**
ClpC^T215I^	0.25	1	0.25	1	0.125
USA300 JE2	0.5	**2**	**1**	**2**	0.5
USA300 JE2 ΔclpP	0.125	**2**	**1–2**	**2**	**>32**

a The tetracycline (Tet) and ADEP 4 MICs were determined in MH broth; the glycopeptide MICs were determined in BHI broth (VA, vancomycin; DAP, daptomycin; Teico, teicoplanin). 1.25 mM CaCl_2_ was added for MIC determinations with VA and DAP. According to the EUCAST guideline (published 01/2022), breakpoints for the tested antibiotics are as follows: Tet > 2 μg/mL; VA > 2 μg/mL; DAP ≥ 1 μg/mL; and Teico > 2 μg/mL. MICs exceeding the EUCAST breakpoints and significantly elevated ADEP-MICs are shown in bold. ND, no data. Grey shading is to distinguish between CmR mutants, revertants and allelic exchange mutants.

### CmR-01, CmR-02, and CmR-03 strains harbor loss-of-function mutations of ClpP.

Since MIC determinations of S. aureus SG511, CmR-02, 02REV, and 03REV revealed remarkable differences in ADEP-susceptibility, we wanted to explore this effect further. Three CmR strains showed cross-resistance to ADEP 2 (CmR-01, CmR-02, CmR-03: MIC >32 μg/mL; wild type: MIC 2 μg/mL), an effect which was alleviated in two revertants (01REV, 02REV; MIC 16 μg/mL) and reversed in one revertant (03REV; MIC 1 μg/mL). MIC determinations of *clpP* deletion strains in different genetic backgrounds of S. aureus revealed a 4-fold increased resistance to CmC compared to the respective wild-type S. aureus strains (Table 5). CmR-01, CmR-02, and CmR-03 carried a nucleotide exchange in *clpP*, yielding ClpP^I29F^. Interestingly, amino acid 29 is located within the hydrophobic pocket of ClpP, a major regulatory site of the protein, which is also known as the binding site of the ADEPs (15, 16). Furthermore, two different suppressor mutations were observed in the cervimycin-susceptible revertants, an additional exchange at position 31, yielding ClpP^I29F/M31I^, and a conservative exchange yielding ClpP^I29V^.

**TABLE 5:** MIC values (μg/mL) for cervimycin C (CmC) in strains that contain only single exchanges in the parental background or knockout mutants^*a*^

Strain	CmC
S. aureus SG511	2
S. aureus SG511 ClpC^T215I^	8
S. aureus SG511 ClpP^I29F^	4
S. aureus SG511 DnaK^A112P^	2
S. aureus SG511 WalK^A243V^	16
S. aureus NCTC 8325-4	16
S. aureus NCTC 8325-4 Δ*clpP*	64
S. aureus USA300 JE2	16
S. aureus USA300 JE2 Δ*clpP*	64
B. subtilis 168	0.25
B. subtilis 168 Δ*clpP*	0.25
B. subtilis 168 Δ*clpC*	0.125
B. subtilis 168 Δ*clpX*	0.125
B. subtilis JH642	1
B. subtilis JH642 Δ*spx*	0.5
B. subtilis JH642 Δ*spx* Δ*clpP*	2

a Broth dilution method was used in Müller Hinton medium and samples were incubated for 24 h.

**FIG 3:**
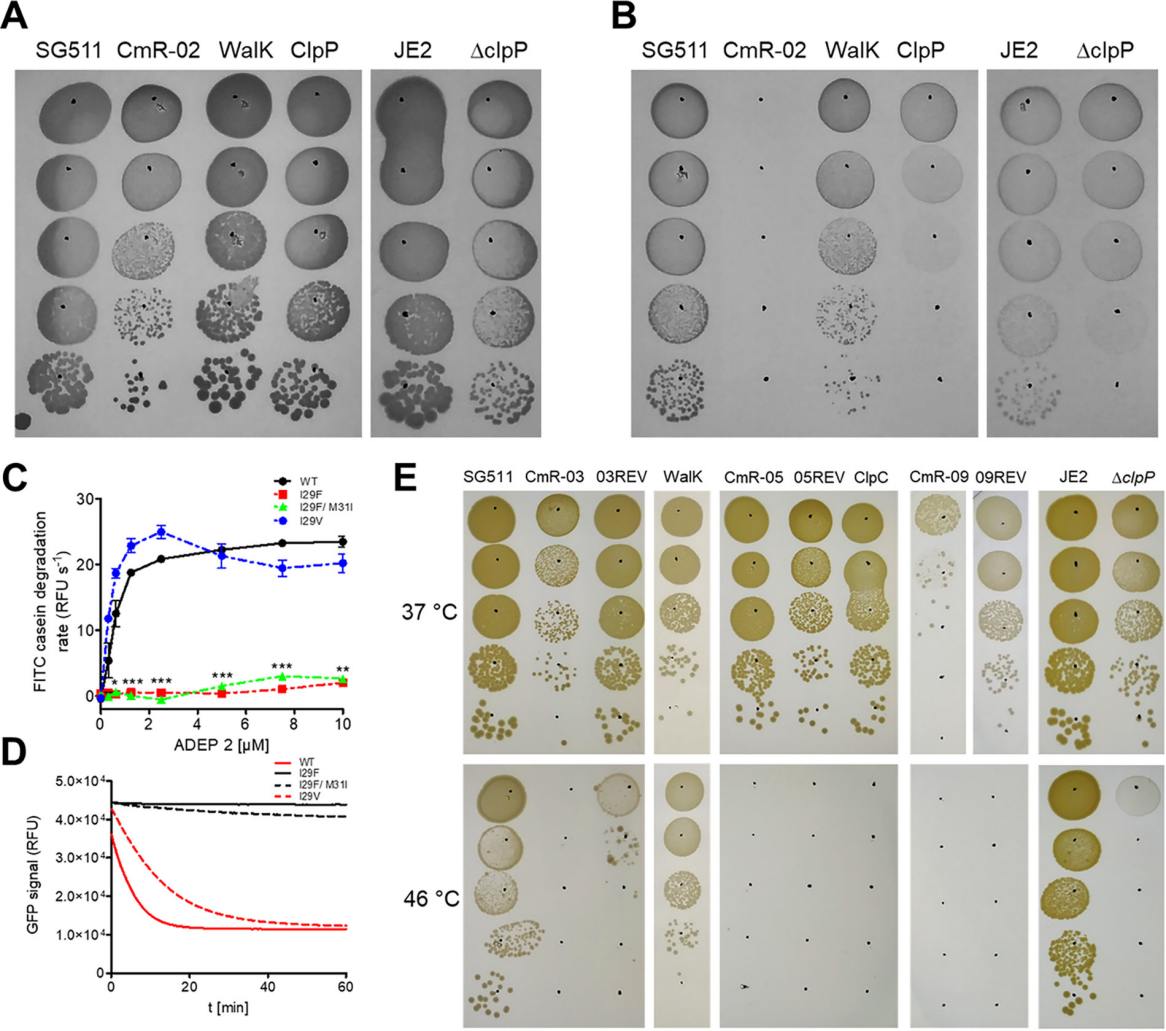
CmR strains harbor loss-of-function mutations in *clpP* or *clpC*. (A and B) A loss-of-function mutation in *clpP* abolishes growth of S. aureus under anaerobic growth conditions. Stationary-phase S. aureus strains were serially diluted 10-fold in 0.9% NaCl (10^−1^- to 10^−5^-fold) and 10 μL of each dilution was spotted on TSA plates. The plates were incubated under aerobic (A) or anaerobic (B) growth conditions. (C) FITC-casein (30 μM) degradation activity of wild-type and mutant SaClpP (1 μM) with increasing concentrations of ADEP 2 (0–10 μM). ADEP responsiveness of two ClpP variants was significantly decreased (*, *P* value ≤ 0.027; **, *P* value ≤ 0.015; ***, *P* value ≤ 0.0004). (D) SaClpXP eGFP-SsrA assay. The natural function of four ClpP-variants (2.8 μM: ClpP^WT^, red line; ClpP^I29F^, black line; ClpP^I29F/M31I^, black dashed line; ClpP^I29V^, red dashed line) was tested with SsrA-tagged eGFP-substrate (0.36 μM) and the ClpX ATPase (2.4 μM). The decrease of the fluorescence signal indicates unfolding or degradation of the substrate. The mutated ClpP variants displayed a consistently decreased GFP degradation activity, compared to the wild type (comparison of the initial 3 min: ***, *P* value ≤ 0.0002). (E) A loss-of-function mutation in *clpC* abolishes growth of S. aureus at elevated temperatures. Cervimycin-resistant and susceptible strains were grown in TSB at 37°C until OD_600_ ~0.5, serially diluted 10-fold in 0.9% NaCl (10^−1^- to 10^−5^-fold), then 10 μL of each dilution was spotted on TSA plates. The plates were incubated at indicated temperatures. WalK, S. aureus WalK^A243V^; ClpP, S. aureus SG511 ClpP^I29F^; ClpC, S. aureus SG511 ClpC^T215I^.

This together with a severe growth defect (17) of the *clpP*-mutation-containing strains CmR-01, CmR-02, and CmR-03 under anaerobic conditions (Fig. 3A and B) indicated a loss of ClpP function in these strains. To verify this, the intrinsic catalytic activity and ADEP-responsiveness of wild-type and ClpP variants, as well as the activity of the ClpP variants in association with the cognate Clp ATPase ClpX were characterized. In principle, ClpP alone can degrade small peptides like Suc-LY-AMC (18) and the activity of all ClpP variants was decreased with this substrate (Fig. S3). Larger substrates like FITC-casein normally cannot be degraded by ClpP alone (15), but degradation can be induced by the addition of ADEPs. ClpP^I29F^ and ClpP^I29F/M31I^ were not activated by ADEP 2 in the FITC-casein assays, whereas ClpP^I29V^ retained good ADEP responsiveness (Fig. 3C and D). To further study the effect of the SNPs on the interaction of ClpP with the cognate Clp ATPase ClpX, we used eGFP-SsrA as model substrate for the natural function of ClpP together with the S. aureus ClpX ATPase. In accordance with the casein degradation data, ClpP^I29F^ and ClpP^I29F/M31I^ did not lead to ClpX-mediated degradation of eGFP-SsrA. In contrast, ClpP^I29V^ nearly possessed wild-type activity. Hence, our results show that I29 of ClpP is important for ADEP- and ClpX-binding, which leads to a loss-of-function of the entire ClpP protease.

It may be hypothesized that cervimycin directly interferes with ClpP, leading to its deregulation, similar to ADEPs. However, we assumed this to be rather unlikely since CmC did not affect the activity of wild-type ClpXP in a GFP-SsrA assay (Fig. S4). In addition to that, MIC determination (Table 5) of a B. subtilis 168 *clpP* deletion mutant yielded the same susceptibility as seen in wild-type cells. However, in *Bacillus* the deletion of ClpP leads to an eventually toxic accumulation of the Spx protein, which is a ClpXP substrate. In order to eliminate this effect, a *spx* deletion mutant was also assayed. Here, the deletion of *spx* alone led to a sensitization of the mutant toward cervimycin because Spx activity can, by its regulatory function, also confer resistance to a wide range of stressors and thereby increase tolerance to certain antibiotics (19). The double *spx clpP* mutant showed an increased resistance to cervimycin and confirmed the results obtained with S. aureus.

### CmR-04 to CmR-09 harbor inactive ClpC variants.

We detected a 4,561-bp deletion of the genes *clpC* and *radA* in CmR-04, and other resistant mutants carried SNPs within *clpC*, leading to ClpC^T215I^ (CmR-05 and CmR-06) or ClpC^P204H^ (CmR-07 to CmR-09) (Table S4). The amino acid exchanges were localized within (T215I) or in proximity (P204H) to the first ATP-binding Walker A motif of the ClpC ATPase. Functionality of the ClpC ATPase was tested in a heat shock assay. The loss of *clpC* leads to a growth defect at 45°C (20), which was true for all strains with the mutated ClpC variants, indicating a loss of ClpC function in these strains (Fig. 3E).

### Effects of cervimycin resistance mutations on the essential WalK histidine kinase.

**FIG 4:**
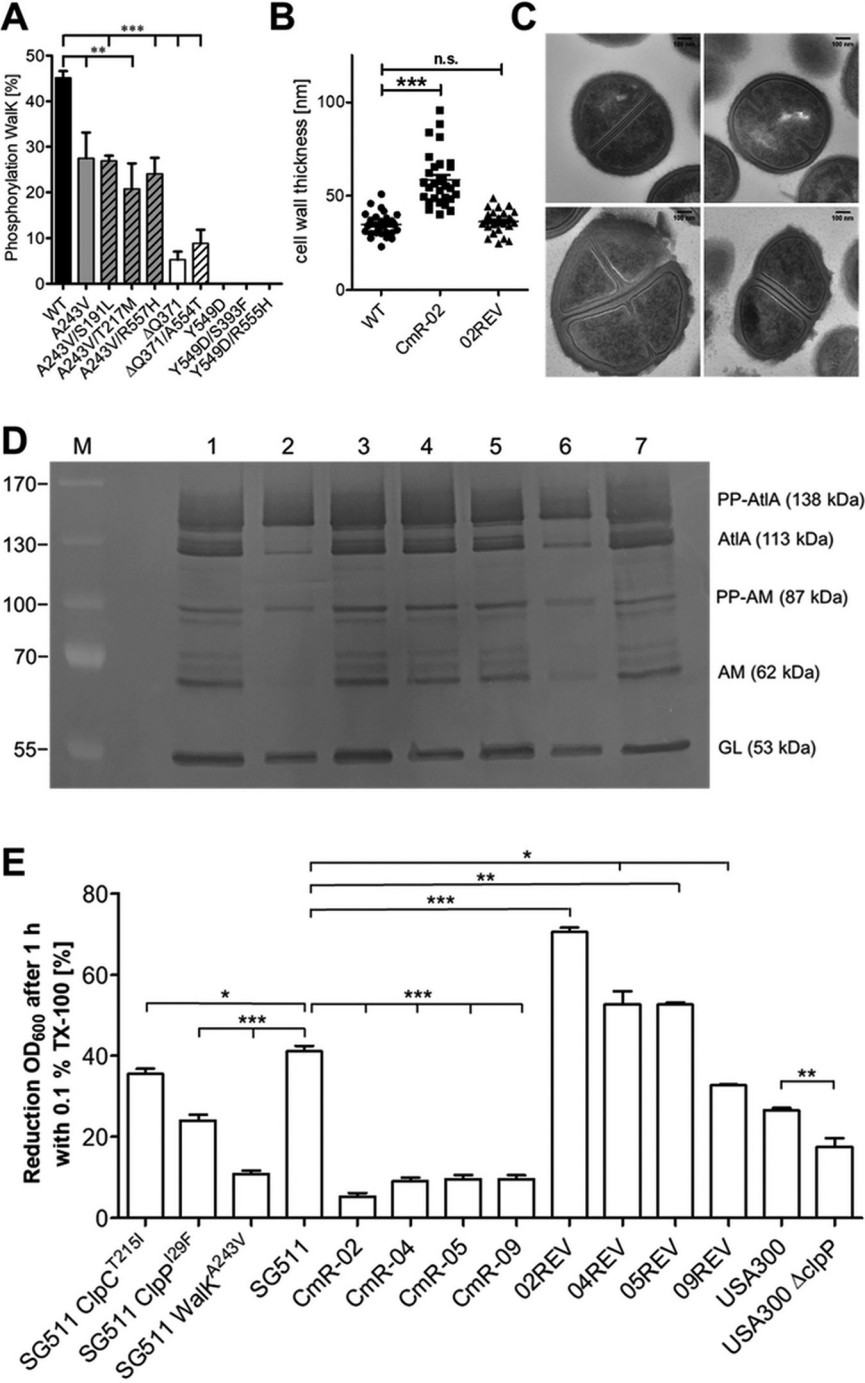
CmR-02 exhibits phenotypic characteristics of a vancomycin-intermediate resistant S. aureus strain. (A) The autophosphorylation of WalK variants from CmR strains and revertants is significantly reduced *in vitro* (**, *P* < 0.018; ***, *P* ≤ 0.0001). (B) Quantification of the cell wall thickness in S. aureus SG511 (WT), CmR-02, and 02REV reveals a significant cell wall thickening in CmR-02 (***, *P* value < 0.0001). (C) Scanning transmission electron microscopy (STEM) of exponentially growing S. aureus strains: top left S. aureus SG511, top right 02REV, bottom CmR-02. (D) Zymogram of LiCl extracts from S. aureus strains SG511 (1), CmR-02 (2), 02REV (3), CmR-04 (4), 04REV (5), CmR-09 (6), and 09REV (7). Numbers to the left of the gel indicate the molecular weights of size standard (PageRuler Plus prestained protein ladder). Dark bands indicate regions of hydrolase activity of the major S. aureus hydrolase AtlA, which appears in different subunits (PP, propeptide; AM, amidase; GL, glucosaminidase). (E) Percentage reduction of the OD_600_ after 60 min of exponential S. aureus cells in PBS with 0.1% Triton X-100. The OD_600_ was normalized to the starting value (* *P* ≤ 0.0272; ** *P* ≤ 0.0063; *** *P* ≤ 0.0001).

All cervimycin-resistant S. aureus strains carried mutations in the essential histidine kinase gene *walK*, yielding either WalK^A243V^, WalK^ΔQ371^ (cervimycin C-resistant strains), or WalK^Y549D^ (cervimycin D-resistant S. aureus strains). Zymographic analysis showed that the autolysin (Atl) activity was severely restricted in the CmR strains, with the exception of CmR-04, and that Triton X-100 induced cell lysis was significantly reduced in all strains (Fig. 4B–E), which might be due to alterations of WalK activity. Electron microscopy of one cervimycin-resistant model strain (CmR-02) and its revertant (02REV), in comparison to the wild-type strain S. aureus SG511, confirmed the VISA phenotype, as CmR-02 possessed a significantly thickened cell wall (wild type: 34.91 ± 5.93 nm, CmR-02: 58.7 ± 13.93 nm, 02REV: 36.41 ± 5.64 nm) (Fig. 4B and C).

We next determined the autophosphorylation activity of the WalK variants as the proportion of autophosphorylated WalK kinase after 30 min under activating conditions. WalK variants from the CmR strains displayed a significantly decreased WalK activity, with WalK^Y549D^ being completely inactive in the *in vitro* test system (Fig. 4A). Surprisingly, the additional *walK* mutations from the revertant strains had no extra effect on WalK autophosphorylation *in vitro*, and resembled the activity of the respective WalK variants from the cervimycin-resistant mutants (Fig. 4A). Unfortunately, we were not able to obtain reproducible results of the influence of cervimycin on the phosphorylation activity of WalK, neither with the Phos-tag activity assay, nor with a ^32^P liposome system. The naphthoquinoid system of cervimycin formed phenolate salts under the assay conditions (pH 8, 5 mM dithiothreitol [DTT]), indicated by a color change from yellow to violet, and in MIC assays the violet form of cervimycin showed poor activity. However, in an agar diffusion assay, induced downregulation of *walK* or *walR* acted synergistically with CmC (Table 6). SsaA and LytM expression were shown to restore cell viability in the absence of WalRK (21). However, overexpression of SsaA or LytM from the xylose-inducible vector pEPSA5 did not promote cervimycin resistance, but acted synergistically with CmC, as well as the overexpression of a constitutively active WalR variant WalR^D55E^ (Table 6). Hence, a lower and a higher WalRK activity increased the susceptibility of S. aureus toward cervimycin.

**TABLE 6:** Downregulation of the histidine kinase gene *walK* or the response regulator gene *walR* increases the susceptibility of S. aureus RN4220 to cervimycin C^*a*^

Altered gene expression	Function	Fold increase diam inhibition zone
Decreased gene expression		
Heat shock response		
*dnaK*	Heat shock protein 70	1.10
Cell wall metabolism		
*pbp3*	Penicillin-binding protein 3	1.00
*smc*	Chromosome segregation protein	1.00
*ftsZ*	Cell division protein	1.08
*walK*	Cell wall metabolism sensor histidine kinase	1.24
*walR*	DNA-binding response regulator	1.30
Δ*atl*	Bifunctional autolysin	1.00
Increased gene expression		
*ssaA*	Secretory antigen precursor	1.10
*lytM*	Glycyl-glycine endopeptidase	1.65
*walR*	DNA-binding response regulator	1.10
*walR^C^*	DNA-binding response regulator, constitutively active (D55E amino acid exchange)	1.24

a Expression levels of indicated genes were increased/decreased with the pEPSA5-system (95). CmC susceptibility was tested in agar diffusion assays in comparison to an empty vector control.

### The *dnaK* mutation has no effect on cervimycin susceptibility.

One CmR strain (CmR-02) carried a nucleotide polymorphism in *dnaK*. The nucleotide exchange C358G in *dnaK* of S. aureus CmR-02 led to an amino acid exchange from alanine to proline at position 112 in the N-terminal nucleotide-binding domain of the protein. However, the reconstitution of the A112P amino acid exchange into the parent strain and the downregulation of *dnaK* using the pEPSA5 system showed no effect on CmC susceptibility (Table 5 and 6).

### Transcriptomic and proteomic analyses reveal major alterations in the cervimycin-resistant mutant CmR-02.

Mutations found in the CmR strains raised both the cervimycin and the vancomycin MICs. Because the regulatory aspect of these mutations is of interest, and also to obtain a more global view on the effect of the mutations found in the cervimycin-resistant mutants, transcriptomic and proteomic analyses of one CmR strain, its susceptible revertant, and the wild-type S. aureus SG511 were performed.

**FIG 5:**
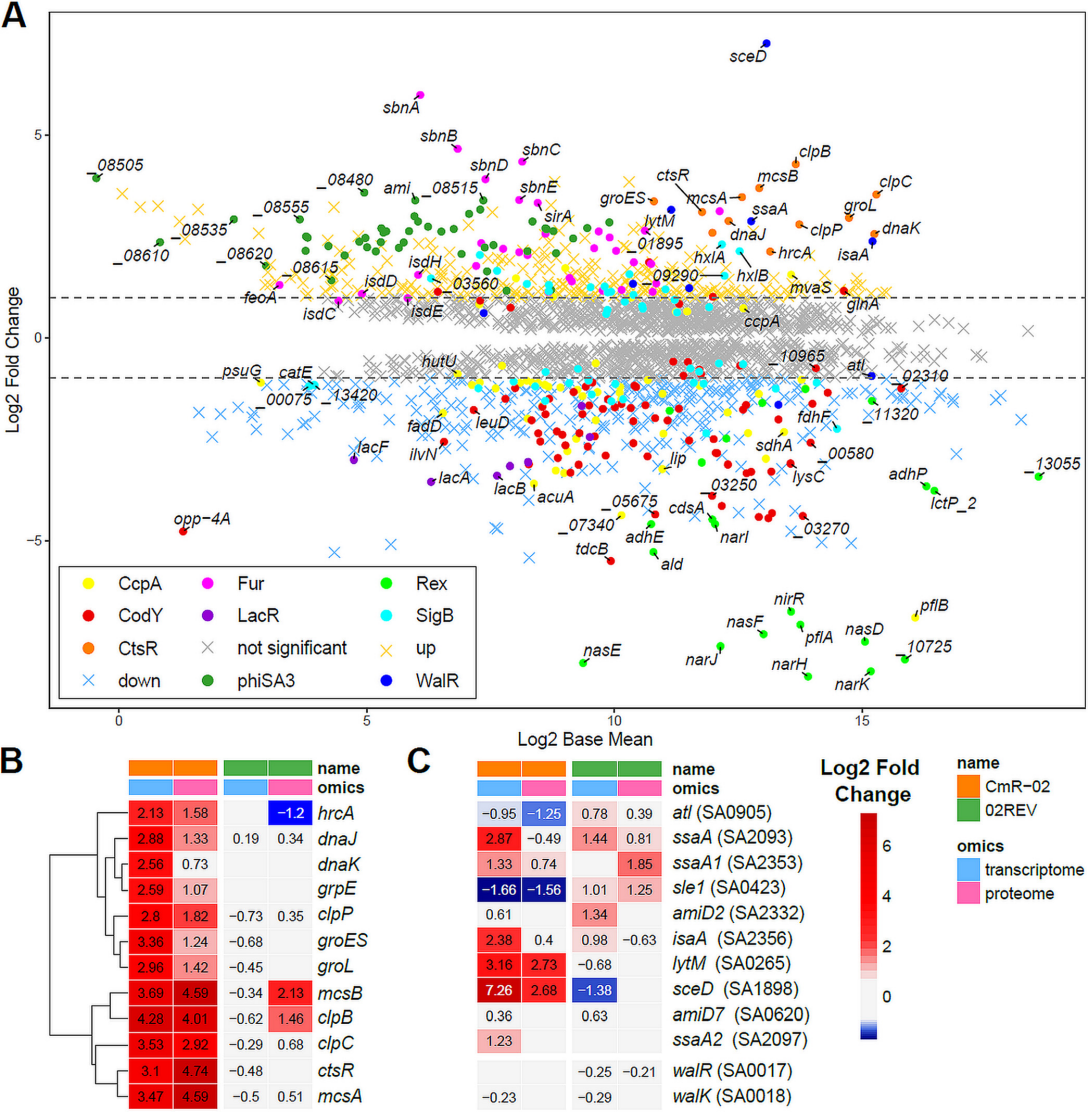
RNA-Seq transcriptomics of S. aureus CmR-02. (A) The expression profile is shown as ratio/intensity scatterplot (M/A-plot: M value, log_2_ fold change; A value, log_2_ base mean), which is based on the differential gene expression analysis. Colored symbols indicate significantly induced or repressed transcripts (M value ≥ 1 or ≤ −1; *P* value ≤ 0.05). Colors refer to the annotated regulator (based on the AureoWiki Database [118]). Regulons with at least seven differentially expressed genes and the phage ΦSA3 encoding genes are shown. Light gray symbols denote transcripts with similar expression levels in comparison to the wild-type S. aureus SG511 Berlin (*P* value ≤ 0.05, log_2_ FC ≤ 1 or ≥ −1). Genes of interest with at least 1.5-fold differential expression are also color-coded (e.g., *atl*). The RNA-seq data of differential transcription of all genes and regulons of CmR-02 are listed in Table S5. Expression profiles of the CtsR/HrcA heat shock operon (B) and the WalR regulon (C) together with the *walRK* genes of S. aureus CmR-02 (orange) and its revertant 02REV (green) reveal major alterations. Up-regulation (log_2_ FC ≥ 1, *P* value ≤ 0.05; red color) and downregulation (log_2_ FC ≤ −1; blue color) on the transcriptomic (light-blue) and proteomic level (light-pink) are compared.

While only minor alterations occurred in the transcriptome of the revertant 02REV in comparison to the S. aureus SG511 wild type, a large number of genes was differentially expressed in the resistant strain CmR-02 (Table 7, Fig. 5A, Fig. S5). This was also confirmed by the proteomic analysis and revealed the broad impact of the mutations in *clpP* and *walK* in CmR-02 (and possibly *dnaK*), which was substantially alleviated by the additional mutations in the revertant 02REV.

**TABLE 7:** Differential expression/translation of genes/proteins in S. aureus CmR-02 and its revertant 02REV in comparison to S. aureus SG511 Berlin^*a*^

	CmR-02		02REV	
Sample	Transcriptome	Proteome	Transcriptome	Proteome
Total	1993	565	1315	439
Up	370 (18.57%)	78 (13.81%)	56 (4.26%)	38 (8.66%)
Down	442 (22.18%)	73 (12.92%)	100 (7.61%)	21 (4.78%)
Sum	812 (40.74%)	151 (26.73%)	156 (11.86%)	59 (13.44%)

a Shown are the total numbers of genes/proteins with a significant detection level (total, *P* value ≤ 0.05), as well as the numbers of significantly upregulated (up, log_2_ fold change ≥ 1) and downregulated (down, log_2_ fold change ≤ −1) genes/proteins.

In CmR-02 the expression of several ABC transporters was repressed, as well as the expression of purine biosynthesis genes, of several regulons of the amino acid biosynthesis (ArgR regulon, S-box), of major parts of the carbohydrate metabolism (MalR, GapR, CcpA-regulon), and of the CodY-regulon (Fig. S6). On a transcriptomic level, also the *lac* operon was repressed. On the other hand, several metabolic pathways were upregulated, like the riboflavin-biosynthesis pathway (FMN-box regulon) and the last four genes of the staphyloxanthin (STX) biosynthesis cluster (KQU62_11530 to KQU62_11545), which might contribute to the intense coloring of CmR-02 on blood agar (Fig. S2).

As expected, major alterations were also detected in the expression of the CtsR/HrcA operon (including *clpP* and *dnaK*) and the WalR regulon (regulated by WalRK). *In vitro* and *in vivo* experiments indicated that the I29F amino acid exchange led to a loss of ClpP function in CmR-02. Expression of the CtsR/HrcA operon, which encodes ClpP and DnaK, was completely derepressed in CmR-02, and the Clp machinery and the chaperones were also more abundant on the protein level (Fig. 5B and Fig. S6), which is in accordance with the observations in the *clpP* mutant of Michel et al. (17). Transcription of *clpX* and *trfA* (sometimes also called *mecA*), which are not encoded in the CtsR/HrcA operon, was also increased (Table S5).

The WalRK TCS positively controls global autolytic activity, particularly via AtlA and the LytM endopeptidase (22). The *walK* mutation of CmR-02 is located in the HAMP-domain coding region, which is a signal transduction domain (23). The reduced autolytic activity in the zymography of CmR cell extracts, together with the reduced cell lysis and the thickened cell walls observed via electron microscopy, suggest a hampered WalK function due to the detected mutation. However, the expression profile of the WalR regulon in CmR-02 was not as explicit as for the CtsR/HrcA operon (Fig. 5C and Fig. S6). Six genes of the WalR regulon were significantly upregulated, the expression of *sle1* was repressed, and the reduced abundance of the major autolysin Atl and the Sle1 amidase was confirmed on the protein level. Interestingly, WalJ (YycJ), which is encoded downstream of the *walRKHI* operon but functionally unrelated and transcribed from a separate promoter (24), was more abundant in CmR-02. However, the consequences of the increased abundance of WalJ remain to be elucidated. Although functions in mismatch repair and chromosome segregation have been described for the genus *Bacillus* (25, 26), a *walJ* knockout mutant did not show any growth related phenotype in S. aureus (24).

In the suppressor mutant, the expression of three hydrolases was increased, and *sceD* expression was decreased. The altered expression of autolysins in 02REV in comparison to CmR-02 indicates an effect of the additional *walK* mutation in the revertant strain, which was not detectable in the *in vitro* activity assay. An upregulation of the VraS regulon, indicating increased cell wall biosynthesis (27) in the resistant mutant, was not observed.

## DISCUSSION

Streptomyces tendae HKI 0179 produces the antibiotic complex of cervimycin, which possesses promising activity against Gram-positive organisms, especially against MRSA and VRE (8). Earlier incorporation studies indicated disturbance of the DNA metabolism, due to cervimycin treatment, although at high concentrations and time-delayed (6). The anthracycline antibiotics dutomycin (28) and polyketomycin (29) are structural homologs of cervimycin, and it has been assumed that these substances might intercalate into DNA, as described for other anthracyclines (30), an activity which was not seen for the cervimycins (6).

We selected cervimycin-resistant strains from S. aureus SG511 Berlin. Cross-resistances to other antibiotics were observed for vancomycin, daptomycin, and teicoplanin for all strains and for acyldepsipeptides (ADEPs) for some strains. Genome sequencing revealed SNPs in the caseinolytic protease gene *clpP* or its cognate ATPase gene *clpC*, the HSP70 gene *dnaK*, and the essential histidine kinase gene *walK*.

ClpP is the proteolytic core of the caseinolytic protease and possesses a key function in protein homeostasis, generally maintaining protein quality, and tightly controlling key regulatory proteins (31), affecting a large number of processes. ClpP forms two-component proteases together with ATPases like ClpX and ClpC (32). This proteolytic machinery degrades various substrates in an ATP-dependent manner (32), allowing adaptation to multiple stresses by degrading accumulated and misfolded proteins (17). The ClpC ATPase forms a complex with the ClpP protease for protein degradation under thermal stress conditions (20). In addition, ClpC is thought to act as a chaperone for the CtsR repressor, which blocks transcription of the CtsR/HrcA operon, encoding *clpCP*, *dnaK*, *groESL*, and more (20). DnaK is a HSP70 chaperone, which catalyzes the folding of newly synthesized polypeptides and the refolding of stress-denatured and aggregated proteins in an ATP-dependent manner (33–35). Although ClpP is not essential, proteolysis of “unemployed,” disintegrated and inactive proteins is a fundamental process of cellular regulation in all organisms (36). Substrates larger than small peptides are degraded by a complex of ClpP and a Clp ATPase, i.e., ClpX or ClpC in S. aureus (37), which also determines the substrate specificity for protein degradation (38, 39).

The ClpP^I29F^ amino acid exchange is located in the N-terminal hydrophobic pocket, which is a major regulatory site of the protease (15, 16). The expression of the Clp machinery is autoregulated, since the CtsR repressor is a substrate of ClpXP (40). However, the CtsR repressor seems to accumulate in an inactive form in CmR-02, an effect which was also seen in a *clpP* deletion mutant (17) and a daptomycin-resistant mutant with a SNP in *clpP* (41), leading to a derepressed transcription of the CtsR/HrcA operon. Several additional characteristics of a *clpP* deletion mutant (17) were also detected in the transcriptome of CmR-02: (i) the derepression of the Fur regulon and the down-regulation of the MntR regulon, which are both involved in metal ion homeostasis; (ii) increased expression of the urease operon; and (iii) reduced expression of the ArcR, NreC, and Rex regulons, which are important in anaerobic growth. In addition, the enhanced expression of the *sirABC* and the *clpC* operons was shared with the previously described *clpP* mutant (42). The loss of ClpP function was also reflected on the proteomic level, because proteins of the CtsR/HrcA operon accumulated in CmR-02, and proteins of the purine/pyrimidine metabolism were depleted, which was also described for *clpP* mutants of different genetic backgrounds (43, 44). The transcriptome and the proteome of the revertant 02REV showed only little similarity to the *clpP* mutant and resembled the wild type.

ClpP activity is an important mechanism to regulate gene expression in S. aureus, which might be the reason for the occurrence of *clpP* mutations in VISA strains (42, 45). Inactivation of either ClpX (46–48) or ClpP (48–50) were found in either vancomycin or daptomycin, and cefotaxime-resistant Gram-positives. In addition to that, a multitude of SNPs was found in ClpP, conferring mostly glycopeptide, β-lactam, or ADEP resistance (41, 42, 50–52). Bæk et al. (48) revealed that S. aureus may under some circumstances benefit from shutting down the Clp proteolytic activity during treatment with antibiotics that target the cell wall, due to the accumulation of ClpP target substrates participating in cell wall metabolism. However, on MIC level, the *clpP* mutation had a lower impact than the *walK* mutation (42), which was also seen for CmR-02 (Table 4). Still, the combination of the *clpP* and the *walK* mutation had a synergistic effect on glycopeptide/daptomycin resistance, with CmR-02 being more resistant than S. aureus WalK^A243V^ (Table 4). Such a strong synergy was not observed for the combination of the same *walK* mutation with the loss-of-function *clpC* mutation in CmR-05 (Table 4).

Three different mutations were identified in the ClpC ATPase gene of different CmR strains, all leading to a complete deletion or an inactivation of the ClpC ATPase (Table 3, Table S4, Fig. 3E). ClpC is important under thermal stress conditions (20) and affects the expression of genes and/or proteins of gluconeogenesis, the pentose-phosphate pathway, pyruvate metabolism, the electron transport chain, nucleotide metabolism, oxidative stress, metal ion homeostasis, stringent response, and programmed cell death (53–56). In addition, ClpCP is responsible for degradation of SsrA-tagged proteins in S. aureus, proteins that arise from stalled ribosomes and may contain translation errors (57); however, ClpXP is also capable to degrade SsrA-tagged substrates *in vitro* (15, 52, 58). In B. subtilis, ClpXP degrades SsrA-tagged proteins (59). Hitherto, the precise role of ClpC in response to drug exposure and antibiotic resistance is not well defined (60). Although *clpC* mutants were generated and extensively studied (20, 53, 54, 61–64), antibiotic resistant mutants with *clpC* mutations have not yet been described for S. aureus. However, in mycobacteria, ClpC1 is the molecular target for the lassopeptide lassomycin and the cyclic peptide cyclomarin A, which activate the ATPase activity, or the proteolysis mediated by the ClpP protease, respectively (65–67). However, these compounds act specifically on the N-terminal domain of mycobacterial ClpC1, while other Gram-positive and Gram-negative bacteria are resistant (65, 66), possibly because of the protective role of ClpC adapter proteins (67). Here, Mycobacterium smegmatis showed poor susceptibility toward cervimycin. So far, a direct interaction between cervimycin and ClpC or ClpP similar to the ADEP antibiotics seems unlikely. Although a *clpP* deletion in S. aureus conferred low-level CmC resistance, a *clpP* mutant in B. subtilis was still susceptible to cervimycin, and cervimycin did not interfere with the degradation of the model substrate eGFP-SsrA in *in vitro* assays using S. aureus ClpXP.

Clinically relevant resistance is often built up through multiple steps, each of which contributes to an increase in resistance (68). The loss-of-function mutation in *clpP* or *clpC* conferred only a two- to 4-fold decrease in cervimycin susceptibility (Table 5). Possible additional contributors were the HSP70 DnaK and the histidine kinase WalK. However, the introduction of the *dnaK* mutation in an antibiotic-susceptible background did not alter the cervimycin susceptibility on the MIC level, and *dnaK* was only mutated in one CmR strain.

Depletion of functional ClpP alters cell wall composition, thickness, and cross-linking (48), which are also extensively influenced by the WalK histidine kinase (22). The WalRK two-component system, which comprises WalK and its cognate response regulator WalR, functions in the regulation of peptidoglycan maturation, cell wall turnover, cell separation and protein secretion, as well as biofilm formation, and positively controls global autolytic activity, particularly via AtlA and the LytM endopeptidase (22). Combinations of mutations in *clpP* and *walK* have been described before and were identified after selection for vancomycin- (42) or daptomycin-resistant mutants (41). Shoji et al. (42) described a laboratory-derived VISA (LR5P1-V3) strain with an N-terminal truncation of ClpP and an amino acid deletion in WalK. Interestingly, the very same amino acid deletion was also detected in a cervimycin-resistant mutant (CmR-04: WalKΔQ371). The nucleotide exchanges found in the *walK* gene of the CmR strains affected the HAMP-domain (A243V), the region between the intracellular PAS-domain and the HisKA-domain (ΔQ371), or the HATPase_c-domain (Y549D) of the essential kinase. The *in vitro* activity of these variants was significantly decreased, although the inactivity of WalK^Y549D^ is doubtful, due to the essentiality of the WalK kinase in S. aureus (69) and the fact that the activating signal for WalK is still unknown and, therefore, could not be incorporated into the *in vitro* assays. There is a high diversity of SNPs in the *walRK* locus, which were mainly identified in VISA strains or daptomycin-resistant mutants (27, 41, 42, 51, 70–72), but were also induced by the lassopeptides siamycin-I and streptomonomicin (73, 74), the membrane active compound chlorhexidine (75), or the redox-active antibiotic actinorhodin (76). No clear clustering of mutations in specific functional regions of these genes has been apparent (77). Numerous mutations were already detected in the HAMP-domain of WalK (27, 78, 79), including amino acid exchanges on position 243 (42, 77, 80). In these clinical VISA, the *walK* mutation was frequently accompanied by mutations in other two-component systems, like VraSR or GraSR, that influence biosynthesis and composition of the cell envelope (42, 77, 80). Mutations in the HATPase_c domain of WalK are more likely to occur in laboratory-derived antibiotic resistant S. aureus strains (72, 80, 81), but are also seen in clinical isolates (27).

The multitude of SNPs observed across all functional WalRK domains leads to the assumption that WalRK activity is reduced in VISA strains (27, 78, 82, 83). This was supported by the *in vitro* activity tests with the WalK variants from the CmR strains, the increased lysis resistance and increased cell wall thickness. WalK depletion also caused this phenotype (22, 82). However, intermediate vancomycin resistance was also seen with a hybrid promoter that increased *walRK* transcription (84), but a constant WalR activation failed to produce a VISA phenotype (82).

In *walHI* deletion mutants, which exhibit reduced WalRK activity, the expression of *atlA* and *sle1* was decreased (83), which is consistent with the gene expression in CmR-02. Also, the upregulation of *isaA* was seen in another S. aureus strain with a mutation in the HAMP-domain of WalK (27). However, most autolysins under the control of WalRK were overexpressed in CmR-02. This contradicts the theory of a decreased WalK activity in CmR-02, but actually an elevated expression of the minor autolysins was also seen for other VISA/glycopeptide-intermediately resistant *Staphylococcus aureus* (GISA) strains (81, 85–87), and these proteins are most probably not involved in cell lysis but in growth and confer plasticity to the cell envelope (21).

Taken together, the activity measurements and phenotype of the CmR strains indicate a reduced WalK activity. However, the identified mutations do not cluster around a possible interaction site between WalK and cervimycin, and tests of WalK activity did not yield consistent results in the presence of cervimycin *in vitro*. The uncoupling of autolysin expression from WalRK through homologous expression of LytM or SsaA was shown to restore cell viability in S. aureus (21). However, neither overexpression of these autolysins nor downregulation of WalK did abrogate the bactericidal effect of CmC in S. aureus RN4220 (Table 6). Therefore, a direct interaction between cervimycin and the kinase seems unlikely as antibiotic mode of action, in contrast, a carefully regulated activity of WalK seems to support survival in the presence of CmC.

In conclusion, we studied the mode of action of the naphthoquinone antibiotic cervimycin, which is bactericidal and exclusively active against Gram-positive bacteria. A selection for cervimycin-resistant S. aureus mutants revealed loss-of-function mutations in the caseinolytic protease gene *clpP* or its cognate Clp ATPase gene *clpC*, and mutations in the essential histidine kinase gene *walK*, eventually leading to a VISA phenotype. However, a direct interaction with cervimycin was neither seen for ClpP nor for WalK, leaving the definitive target of cervimycin to be determined. Omics data of cervimycin treated S. aureus might give further insight into what happens inside the cell in response to cervimycin, and whether there are analogies to antibiotics with known mode of action. Nevertheless, the combination of *clp* and *walK* mutations in the cervimycin-resistant mutants might suggest either a target structure associated with growth and cell wall biosynthesis, like lipid II, which is the target of vancomycin, or protection of the target site from cervimycin by a thickened cell envelope. The VISA phenotype, based on the mutations in *walK*, might also have a compensatory effect toward cervimycin activity, either by slowing down growth, or by modulation of the cell wall and central metabolism.

## MATERIALS AND METHODS

### Construction of cervimycin-resistant mutants.

S. aureus SG511 Berlin was serially passaged by subculturing the strain in 1/100 dilutions each day in medium supplemented with cervimycin C or D (for 15–28 days). Cervimycin was added at 0.5× MIC (1 μg/mL) and increased 1.5- to 2-fold each day up to 64× MIC. This culture was plated on TSA with 120 μg/mL cervimycin and incubated overnight at 37°C. Ten sibling clones of three different passaging experiments were isolated and three clones (CmR strains, CmR-01 to CmR-09) of each lineage were analyzed thoroughly. Revertants (01REV–09REV) were generated by passaging the CmR strains in the absence of cervimycin for several times.

Genomic DNA was isolated from nine CmR mutants and their respective revertants using the Master Pure Gram-positive DNA purification kit (Epicentre Biotechnologies) with one modification: lysostaphin solution (30 ng/mL) was added during the lysis step. Whole-genome sequencing of the wild-type strain S. aureus SG511 (PacBio) was previously described (88). Illumina technology was used for resequencing of the mutant strains, according to Kehl et al. (89). Genomes of all sequenced mutants were assembled *de novo* and were aligned to the parent strain S. aureus SG511 Berlin (NZ_CP076660) using the Geneious mapping and assembly tool. SNPs were called on the mapped reads by comparing the mutant genomes with the parent strain using Geneious SNP caller (Geneious R10.2.6; https://www.geneious.com [90]). Mutations were verified via PCR and Sanger sequencing. Bacterial strains and primers used in this study are listed in Tables S2 and S3.

### Allelic exchange using the pMAD-system.

The pMAD-vector is a temperature-sensitive shuttle vector for markerless allelic replacement in Gram-positive bacteria (91). The pMAD-system was used to introduce SNPs identified in cervimycin-resistant mutants into the genome of S. aureus SG511. Fragments of the respective genes (500 bp) were amplified via PCR (primers are listed in Table S3), cloned into the pMAD-vector, transformed into E. coli DC10B, and verified via sequencing. The constructs were then transformed into S. aureus RN4220, and afterwards transduced into S. aureus SG511 or CmR-01 using phage 85. For the allelic exchange, a blue colony of S. aureus, containing the respective pMAD-vector, was inoculated into 5 mL brain heart infusion (BHI) broth with 10 μg/mL erythromycin and grown at 30°C. Subsequently, the temperature was shifted to the nonpermissive temperature (42 or 44°C) or a 1:10 dilution was directly plated on BHI agar containing erythromycin and X-gal (300 mg/L) and incubated at the nonpermissive temperature. Then, a blue colony was inoculated into 5 mL BHI without antibiotic and the procedure was repeated. This time the culture was diluted 1:10,000-fold and plated on BHI agar containing only X-gal. The presence of specific mutations in white colonies was verified via PCR and sequencing. Whole-genome sequencing was performed for S. aureus WalK^A243V^ and S. aureus SG511 ClpP^I29F^ using Illumina technology as described above.

### Phenotyping of CmR strains and revertants.

The colony morphology of the different S. aureus isolates was analyzed after plating of serially diluted stationary cells on Columbia blood agar and overnight incubation at 37°C. The colony size was quantified using the ImageJ Software (92). Growth of S. aureus at different temperatures was carried out as described earlier (93). Briefly, TSB was inoculated with 1% of a S. aureus overnight preculture and incubated at 37°C until reaching an optical density at 600 nm wavelength (OD_600_) of 0.5. Serial dilutions (0.9% NaCl, 10-fold dilutions, 10 μL each) of the culture were spotted on TSA plates. The agar plates were incubated at different temperatures until visible growth occurred, usually overnight. Anaerobic growth conditions were generated using the GasPak system (Becton, Dickinson, Heidelberg, Germany).

### Electron microscopy.

For scanning transmission electron microscopy (STEM) sample preparation, BHI was inoculated with 1% of a S. aureus SG511, CmR-02, or 02REV preculture and incubated with aeration at 37°C until reaching an OD_600_ of 1.0. Cells were harvested (8,000 × *g*, 5 min, room temperature (RT)), washed with 1 mL ultrapure water, resuspended in fixation buffer (4% paraformaldehyde and 2.5% glutaraldehyde in cacodylate buffer, pH 7.4) and incubated at 4°C overnight. After fixation, bacteria were washed with cacodylate buffer before treatment with osmium (1% osmium tetroxide, 0.8% ferricyanate in 0.1 M cacodylate buffer) for 2 h at room temperature. Bacteria were pelleted and dehydrated in ethanol. During dehydration, at 70% ethanol, 0.5% uranyl acetate was added for 1 h. After dehydration in ethanol, samples were incubated in propylene oxide before embedding in EPON. Ultrathin sections were collected onto Formvar/Carbon coated copper slot grids, and counter stained with uranyl acetate and lead citrate. Imaging was done using a Zeiss Crossbeam 550 at 30 kV with STEM detector.

### Firefly luciferase assay.

Construction and validation of whole-cell antibiotic biosensors was reported earlier (12). The assay allows a high-throughput diagnosis of antibiotic interference in the major metabolic pathways of bacteria and was performed as described previously (94). Briefly, the promoter and upstream regions of certain genes induced selectively by reference antibiotics were fused to the firefly luciferase reporter gene in B. subtilis 1S34. Specifically, the promoter regions of *ypuA*, *liaI*, *bmrC* (*yheI*), *helD*, and *yorB* signaled cell membrane stress, interference with the undecaprenyl-phosphate cycle, translation stalling, inhibition of RNA synthesis, and interference with DNA synthesis or structure, respectively. Overnight cultures grown in LB medium with 5 μg/mL erythromycin at 37°C with shaking were diluted to an OD_600_ of 0.05 in LB or BMM (*bmrC*) with 5 μg/mL erythromycin and were grown as before to an OD_600_ of approximately 0.8 or 0.4 (*bmrC*), respectively. Serial 2-fold dilutions of cervimycin C (0.002–4 μg/mL) were inoculated with suspensions of the reporter strains in white 96-well flat-bottom polystyrene microtiter plates and incubated at 37°C for different periods of time depending on the induction kinetics of the respective strain (12). Subsequently, flash luminescence was measured in a Tecan M200 microtiter plate reader after injecting 60 μL of a 2 mM luciferin solution.

### Antimicrobial susceptibility testing.

Determination of MICs was performed in polystyrene round-bottom microtiter plates (Greiner, Frickenhausen, Germany) using cation-adjusted Müller Hinton (MH) broth or BHI broth. An inoculum of 5 × 10^5^ CFU/mL was employed in the arithmetic broth microdilution method. Due to the high mutation rate, only 1 × 10^5^ CFU/mL were used for ADEP MICs. For dilution of Mycobacterium smegmatis, 0.05% Tween 80 was added to the MH broth. For MIC testing of vancomycin and daptomycin (Vancomycin CP LILLY, Lilly GmbH, Bad Homburg, Germany; Cubicin, Novartis Pharma GmbH, Nürnberg, Germany), CaCl_2_ was added to all cultures to a final concentration of 1.25 mM (51). The MIC was defined as the lowest concentration of the antibiotic that inhibited visible growth after 24 h of incubation at 37°C. Susceptibility testing of S. aureus strains against vancomycin was performed on BHI gradient agar supplemented with 1.25 mM CaCl_2_. Material of several colonies was resuspended to an optical density at 600 nm wavelength of 0.1 in 0.9% NaCl and the gradient agar plates were inoculated using a cotton swab.

### Antisense-based susceptibility profiling.

The vector-based antisense clones were created in S. aureus RN4220 by Forsyth et al. (95) and provided by Merck (USA). The pEPSA5 vector is xylose-inducible and selected by 34 μg/mL chloramphenicol. Antisense strains were inoculated into LB broth and grown at 37°C without shaking. Next, 20 μL of the cell suspension were diluted in 4 mL 0.9% potassium chloride, poured on LB agar with chloramphenicol and xylose, allowed to settle for 2 min, and surplus suspension was removed. The inocula of antisense strains and the empty vector control were adjusted to yield similar inhibition zones with the control antibiotics. Antibiotic discs with 5 μg CIP, 30 μg VA, and 25 μg cervimycin were added. Inhibition zones were measured after overnight incubation at 37°C. The same procedure was used to measure the effect of protein-overexpression on antibiotic susceptibility. The primers used to construct the *lytM*- and *ssaA*-overexpression strains are listed in Table S3. The pEPSA5-ssaA vector was cloned using S. aureus VC40 as the template (96), and the SNP present in this strain was repaired using the QuikChange lightning kit (Agilent) and the primers listed in Table S3.

### Zymographic analysis of autolysin extracts and Triton X-100-induced autolysis.

Zymographic analyses of autolysin extracts were performed according to Gajdiss et al. (97). For Triton X-100-induced lysis experiments, S. aureus strains were grown in tryptic soy broth (TSB) with aeration at 37°C. At an OD_600_ of 0.6, the cells were cooled on ice and harvested by centrifugation. The cell pellets were washed once with ultrapure water, resuspended in phosphate-buffered saline (pH 7), and autolysis was induced by the addition of 0.1% Triton X-100. The cells were incubated at 37°C and lysis was observed photometrically at 600 nm every 30 min.

### Transcriptomics.

For RNA extraction, cultures of S. aureus SG511, CmR-02, and 02REV were grown in TSB at 37°C with shaking until an OD_600_ of 1.0 was reached. Next, 20 mL cultures were harvested (5 min, 8,000 × *g*, RT), the cells were resuspended in 1 mL RNA protect buffer (New England Biolabs, Frankfurt am Main, Germany) and incubated for 5 min at RT. The cells were resuspended in 200 μL Tris-EDTA (TE) buffer with 25 μg lysostaphin and cell lysis was achieved by incubation at 37°C for 30 min. RNA was extracted using the Monarch total RNA miniprep kit (New England Biolabs) following the manufacturer’s instructions and stored at −80°C. Quality and quantity of total RNA were determined by agarose gel electrophoresis. Per replicate, a total amount of 400 ng RNA was subjected to rRNA depletion. For rRNA depletion and cDNA library construction, the Illumina Stranded Total RNA Prep Kit and the Ribo-Zero Plus Kit were used according to the manufacturer’s instructions. Libraries were sequenced as single-read (101 bp read length) on a NovaSeq 6000 platform (Illumina) at a depth of 10.1 to 24.9 million reads each. Library preparation and sequencing procedures were performed by the same individual and a design aimed to minimize technical batch effects was chosen. RNA sequencing was performed by the Institute for Medical Microbiology (part of the NGS Competence Center NCCT (Tübingen, Germany)) while data management, including data storage of raw data for this project were done by the Quantitative Biology Center (QBiC, Tübingen, Germany).

Sequencing statistics, including the quality per base and adapter content assessment of resulting transcriptome sequencing data were conducted with FastQC v0.11.5 (98). All reads mappings were performed against the reference strain of S. aureus SG511 Berlin (RefSeq ID NZ_CP076660.1). The mappings of all samples were conducted with HISAT2 v2.1.0 (99). Spliced alignment of reads was disabled and library type was set to reverse (HISAT2 parameter --no-spliced-alignment and --rna-strandness R). The resulting mapping files in SAM format were converted to BAM format using SAMtools v1.9 (100). Mapping statistics, including percentage of mapped reads and fraction exonic region coverage, were conducted with the RNA-Seq module of QualiMap2 v2.2.2-dev (101). Gene counts for all samples were computed with featureCounts v1.6.4 (102) based on the annotation of the respective reference genome, where the selected feature type was set to transcript records (featureCounts parameter -t transcript). To assess variability of the replicates of each condition, a principal-component analysis (PCA) was conducted with the DESeq2 package v1.20.0 (103).

For the computation of genes differentially expressed between the wild-type strain and the strains S. aureus SG511, CmR-02, and 02REV, respectively, DESeq2 v1.20.0 (103) was applied to the absolute gene counts as computed with featureCounts. Genes with low counts (less than 10 reads) over all replicates in both media were filtered prior to differential expression analysis. For differences between each strain, genes with an adjusted *P*-value (false discovery rate [FDR]) < 0.05 and absolute log_2_ fold change (FC) ≥ 1 were reported as differentially expressed.

### Proteomics.

Total protein extraction was performed from S. aureus cultures in TSB using the same cultivation conditions as for total RNA extraction. Cells were harvested (6,000 × *g*, 10 min, 4°C) and washed with 1 mL ice-cold ultrapure water. The cells were resuspended in 1 mL SDS buffer (4% wt/vol SDS, in 100 mM Tris-HCl, pH 8.0), incubated at 95°C for 10 min, chilled on ice for 5 min, transferred into bead beating tubes with 0.1 mm glass beads (Carl Roth GmbH, Karlsruhe, Germany), and disrupted via bead beating (3 × 30 s, 5,000 rpm with pauses of 90 s). Cell debris was removed via centrifugation (11,000 × *g*, 5 min, 4°C) and the supernatant was treated with 10 mM DTT (45 min, 650 rpm, RT) and 5.5 mM iodoacetamide (45 min, 650 rpm, RT). Debris was removed (12,000 × *g*, 15 min, RT) and proteins were precipitated with eight volumes of ice-cold acetone/methanol (8:1 vol/vol mixture) at −20°C overnight. Precipitated proteins were harvested by centrifugation (13,000 × *g*, 10 min, RT) and washed 3 times with 1 mL 80% aqueous acetone. Residual acetone was evaporated, protein pellets were resuspended in denaturation buffer (6 M urea, 2 M thiourea in 10 mM Tris-HCl, pH 7.5), and protein concentration was determined using the Bradford method. Ten micrograms of proteins per sample were purified on a NuPAGE 12% gel (Invitrogen) and Coomassie-stained gel pieces were digested in gel with trypsin (104). Desalted peptide mixtures (105) were separated on an Easy-nLC 1200 system coupled to a quadrupole Orbitrap Exploris 480 mass spectrometer (all Thermo Fisher Scientific) as described previously (106) with slight modifications: peptides were separated using a 57-min segmented gradient from 10% to 33, 50, and 90% of HPLC solvent B (80% acetonitrile in 0.1% formic acid) in HPLC solvent A (0.1% formic acid) at a flow rate of 200 nl/min. The mass spectrometer was operated in data-dependent mode, collecting MS spectra in the Orbitrap mass analyzer (60,000 resolution, 300–1,750 *m/z* range) with an automatic gain control (AGC) set to standard and a maximum ion injection time set to automatic. The 20 most intense precursor ions were sequentially fragmented with a normalized collision energy of 28 in each scan cycle using higher energy collisional dissociation (HCD) fragmentation. In all measurements, sequenced precursor masses were excluded from further selection for 30 s. MS/MS spectra were recorded with a resolution of 15,000, whereby fill time was set to automatic.

Acquired MS spectra were processed with MaxQuant software package v1.6.14.0 (107) with integrated Andromeda search engine (108). Database search was performed against a Staphylococcus aureus database obtained from Uniprot (https://www.uniprot.org/; 2.889 protein entries), and 286 commonly observed contaminants. Endoprotease trypsin was defined as protease with a maximum of two missed cleavages. Oxidation of methionine, and protein N-terminal acetylation were specified as variable modifications. Carbamidomethylation on cysteine was set as fixed modification. Initial maximum allowed mass tolerance was set to 4.5 ppm for precursor ions and 20 ppm for fragment ions. Peptide, protein and modification site identifications were reported at a FDR of 0.01, estimated by the target-decoy approach (109). The Intensity Based Absolute Quantification (iBAQ) and Label-Free Quantification (LFQ) algorithms were enabled, as was the “match between runs” option (110, 111). The generated data were analyzed using the LFQ-Analyst (https://bioinformatics.erc.monash.edu/apps/LFQ-Analyst/).

### Cloning, overexpression, and purification of S. aureus WalK and ClpXP.

Cloning and purification of the full-length WalK histidine kinase (112, 113) and the ClpX ATPase (114) was described before. WalK- and ClpP variants (listed in Table 3) were created using the same protocol and the primers listed in Table S3. Shortly, the genes of interest were cloned into pET22bΔpelB, transformed into E. coli C43(DE3) pREP4groESL(MT) for expression of membrane proteins, or E. coli BL21 LOBSTR (115) for expression of cytoplasmic proteins, and overexpressed overnight at 30°C. Cells were harvested, lysed by ultrasonication, and membrane proteins were removed from the membrane using n-dodecyl-β-D-maltoside (DDM). Proteins were purified via Ni-NTA affinity chromatography. The WalK proteins were dialyzed (50 mM HEPES [N-2-hydroxyethylpiperazine-N′-2-ethanesulfonic acid], 200 mM KCl, 50% [vol/vol] glycerol, pH 8) using Slide-A-Lyzer dialysis cassettes (Thermo Fisher Scientific), supplemented with glycerol to a final concentration of 50% and stored at −20°C. ClpX and ClpP were dialyzed in sodium phosphate buffer (25 mM sodium phosphate pH 8.0, 100 mM KCl, 5% glycerol [vol/vol]).

### ClpP activity assays.

The ClpP peptidase activity, ClpP FITC-casein, and SaClpXP assays were performed in triplicates as previously described (52). For the ClpP peptidase activity assay, ClpP (final concentration 2 μM) was preincubated in assay buffer (100 mM HEPES, pH 7.0, 100 mM NaCl) at 32°C. Then, 50 μL Suc-Leu-Tyr-7-amino-4-methylcoumarin (Suc-LY-AMC) was added to a final concentration of 200 μM and a total volume of 100 μL. The reaction was measured every minute using an Infinite M200Pro plate reader (Tecan), following the increase of fluorescence (excitation, 380 nm; emission, 440 nm). Fluorescence was plotted over time and slopes of the initial segment of the fluorescence-time plot were calculated via linear regression.

The ClpP FITC-casein assay was performed in black flat-bottomed 96-well plates (Sarstedt). Next, 1 μL ADEP 2 (final concentration 0 to 10 μM) was incubated with 49 μL ClpP (final concentration 1 μM) in assay buffer at 37°C. FITC-casein was added to a final concentration of 30 μM and a total volume of 100 μL. The reaction was measured using an infinite M200Pro plate reader (Tecan), following the increase of fluorescence (excitation, 494 nm; emission, 521 nm). The initial 300 s were used for data analysis. A casein solution without ClpP served as reference.

The SaClpXP assay was performed in PZ buffer (PZ: 25 mM HEPES, 200 mM KCl, 5 mM MgCl_2_, 1 mM DTT, 10% [vol/vol] glycerol, pH 7.6) in a white flat-bottom well plate (Greiner, Frickenhausen, Germany) with a 100 μL reaction volume at 30°C. The reaction contained 2.8 μM ClpP, 2.4 μM ClpX, 0.36 μM eGFP-SsrA, and an ATP regeneration system (4 mM ATP, 16 mM creatine phosphate, 20 U/mL creatine phosphokinase). eGFP-SsrA degradation was measured in an Infinite M200 Pro plate reader (Tecan) with an exciting wavelength of 465 nm and a measured emission of 535 nm. eGFP-SsrA unfolding activity was derived as initial slopes in fluorescence time courses.

### Detection of WalK phosphorylation by Phos-tag SDS-PAGE or radioactively labeled ATP.

Auto-phosphorylation and phosphorylation detection of WalK was performed in Triton X-100 micelles and via Phos-tag PAGE or in phospholipid liposomes with radioactively labeled ATP as described in Gajdiss et al. (113).

### Data availability.

The whole-genome sequencing data of the CmR strains, the revertants, and the allelic exchange mutants S. aureus WalK^A243V^ and S. aureus SG511 ClpP^I29F^ were deposited in NCBI under the BioProject number PRJNA852436. All high-throughput RNA-sequencing data in this publication have been deposited in NCBI’s Gene Expression Omnibus (116) and are accessible under accession number GSE206309. The mass spectrometry proteomics data have been deposited to the ProteomeXchange Consortium via the PRIDE (117) partner repository with the data set identifier PXD034970.
