# Supplementary material for: Cervimycin-Resistant Staphylococcus aureus Strains Display Vancomycin-Intermediate Resistant Phenotypes
Source: Microbiol Spectr. 2022 Sep 29;10(5):e02567-22. doi: 10.1128/spectrum.02567-22 (PMC9603734; doi:10.1128/spectrum.02567-22)
Supplement: Supplemental file 1 — Fig. S1 to S6 and Tables S1 to S5. Download spectrum.02567-22-s0001.pdf, PDF file, 2.5 MB [file spectrum.02567-22-s0001.pdf]

Supplementary Material

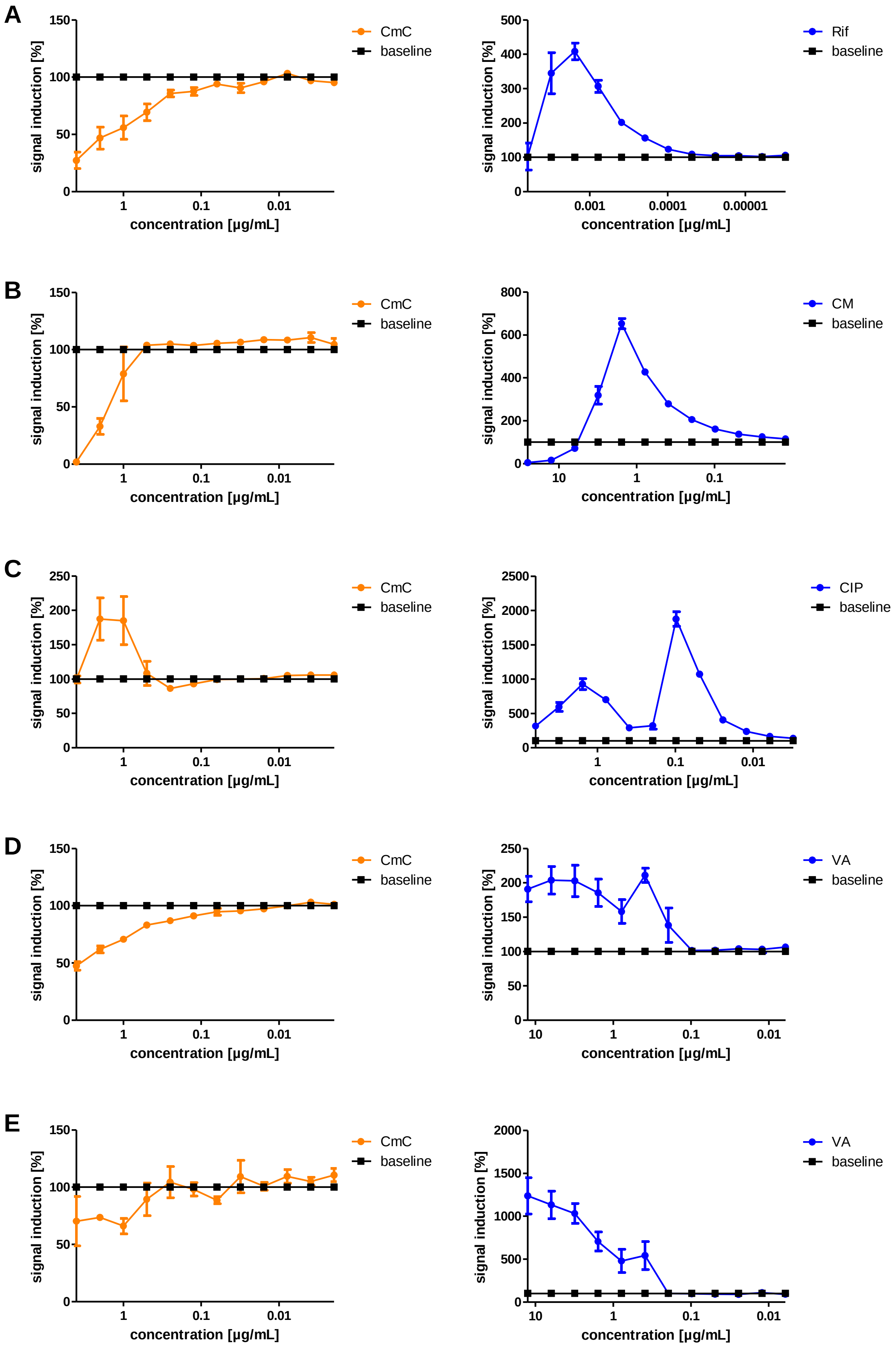
A
B
C
D
E
signal induction [%]
concentration [µg/mL]
CmC
baseline
Rif
CM
CIP
VA
0
50
100
150
200
250
300
400
500
600
800
1000
1500
2000
2500
10
1
0.1
0.01
0.001
0.0001
0.00001

Figure S1: Biomarker induction assay with cervimycin C (left, orange line) and positive controls (right, blue line). Antibiotic interference in the major biosynthetic pathways of bacteria was tested with firefly luciferase reporter strains, (**A**) *helD* strain (RNA reporter), (**B**) *bmrC* strain (translation stalling reporter), (**C**) *yorB* strain (DNA reporter), (**D**, **E**) *ypuA* and *liaI* strain (cell envelope reporters) (1). Exponentially growing *Bacillus subtilis* reporter strains were incubated with indicated antibiotic concentrations, and induction of the promoters was measured as luminescence signal. Signal induction is displayed as the percentage ratio of the untreated control versus the treated sample. Antibiotics with known mode of action were used as positive controls: Rif (rifampicin); CM (chloramphenicol), CIP (ciprofloxacin), VA (Vancomycin).

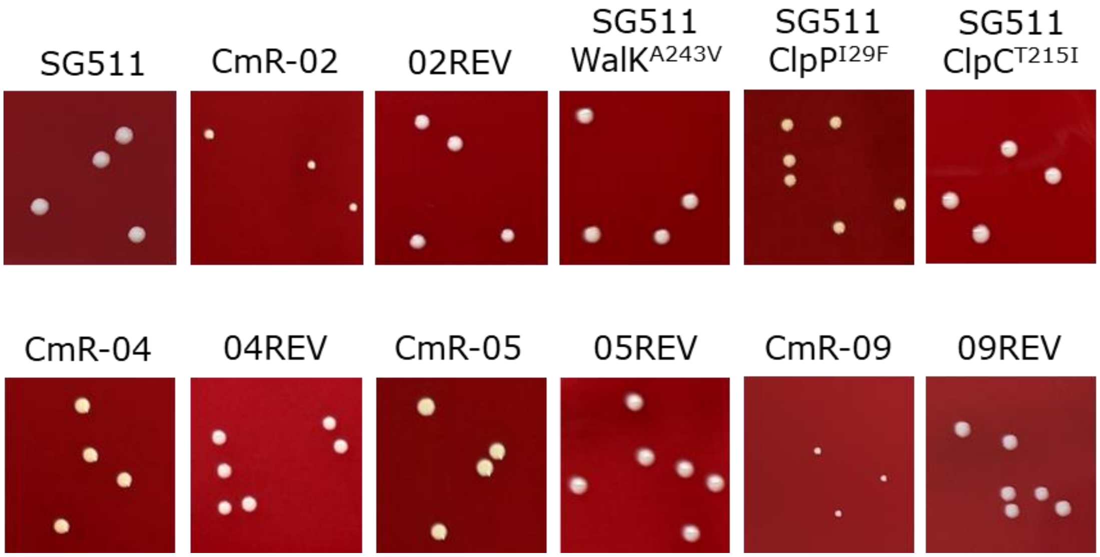

Figure S2: Colony morphology of cervimycin resistant mutants (CmR), cervimycin-susceptible revertants (REV), and allelic exchange mutants with SNPs in *walK*, *clpP* or *clpC* after over-night incubation at 37 °C. Stationary phase cells were diluted in 0.9% NaCl and plated on Columbia blood agar. A significant decrease of the colony diameter was observed for some mutants, and an intense yellow color.

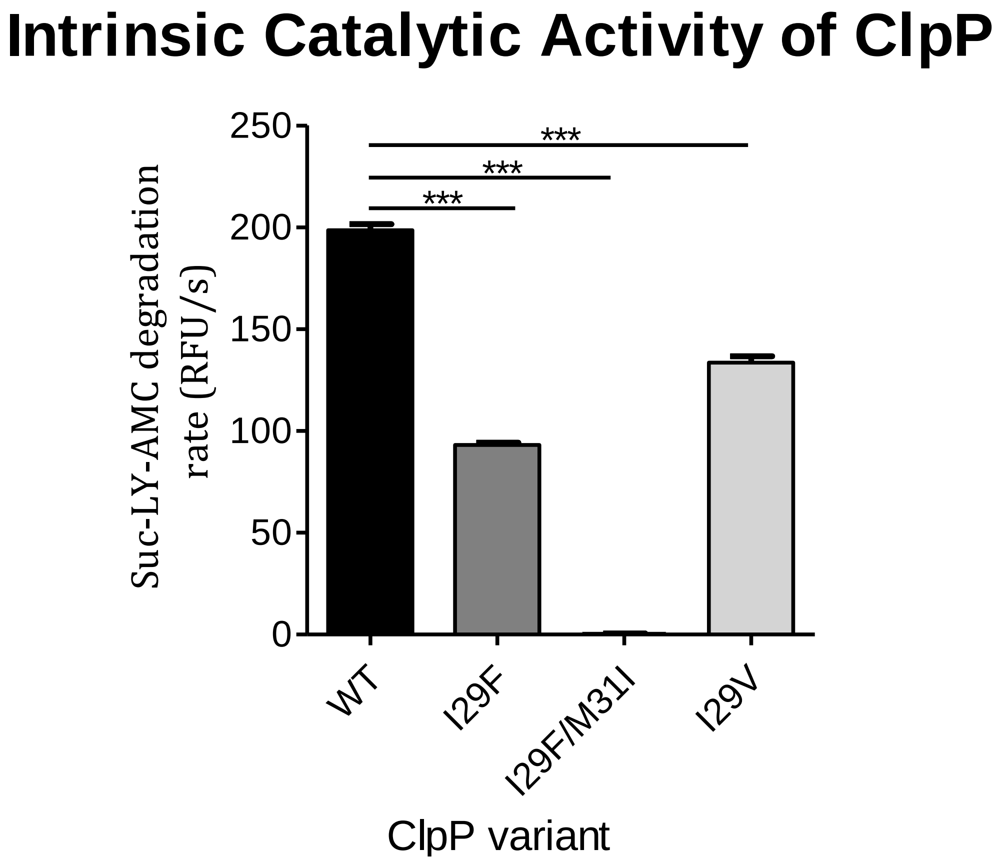

Figure S3: Suc-LY-AMC (200 µM) degradation by wild-type and mutant SaClpP (2 µM). Activity was tested with ClpP-variants from *S. aureus* SG511 (WT), the cervimycin C-resistant mutant CmR-02 (substitution I29F), and the cervimycin-susceptible revertant strains 02REV (I29F/M31I) and 03REV (I29V). Significantly decreased intrinsic catalytic activity was seen for all mutated ClpP variants (***, p value ≤ 0.001).

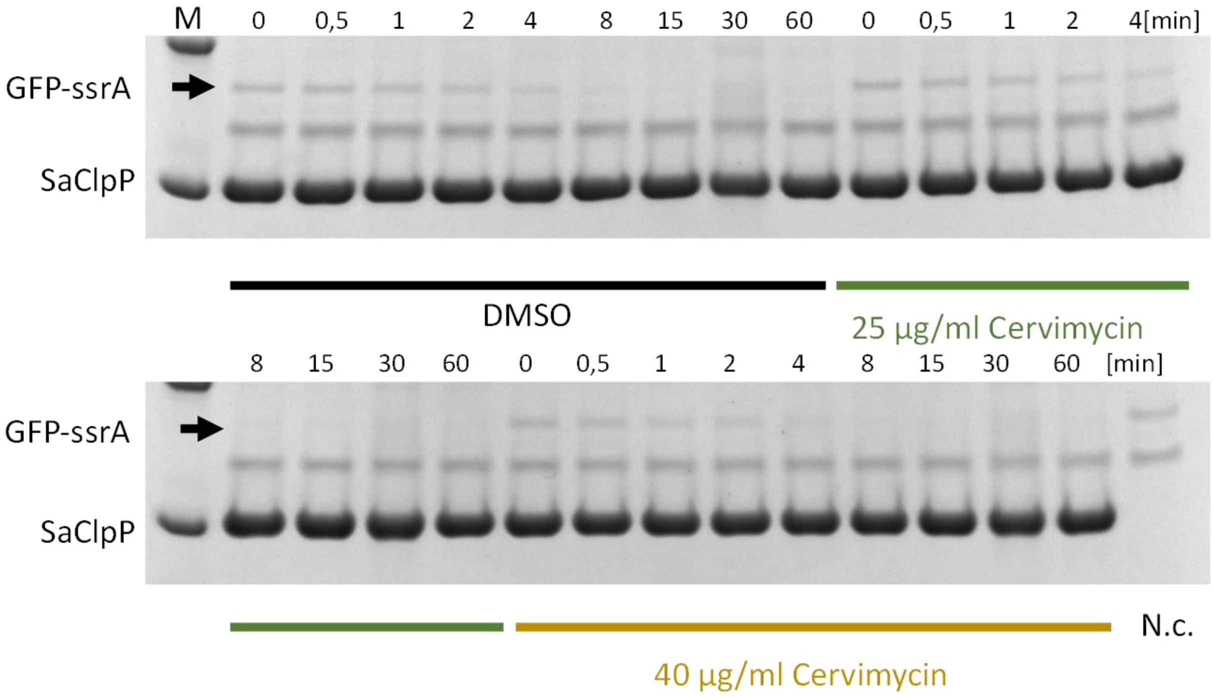

Figure S4: eGFP-SsrA degradation by ClpXP in absence and presence of cervimycin C (Cervimycin). Because of the auto-fluorescence of cervimycin, GFP-SsrA degradation was followed via SDS PAGE. eGFP-SsrA was incubated with SaClpXP in PZ buffer at 30 °C together with cervimycin (25 or 40 µg/mL) and samples were taken after indicated time intervals. Protein degradation activity was immediately stopped by addition of Laemmli sample buffer, and boiling. Samples were separated on a Coomassie-stained SDS PAGE; arrow, eGFP-SsrA; lower band, SaClpP; middle band, SaClpX; N.c., negative control without ClpP.

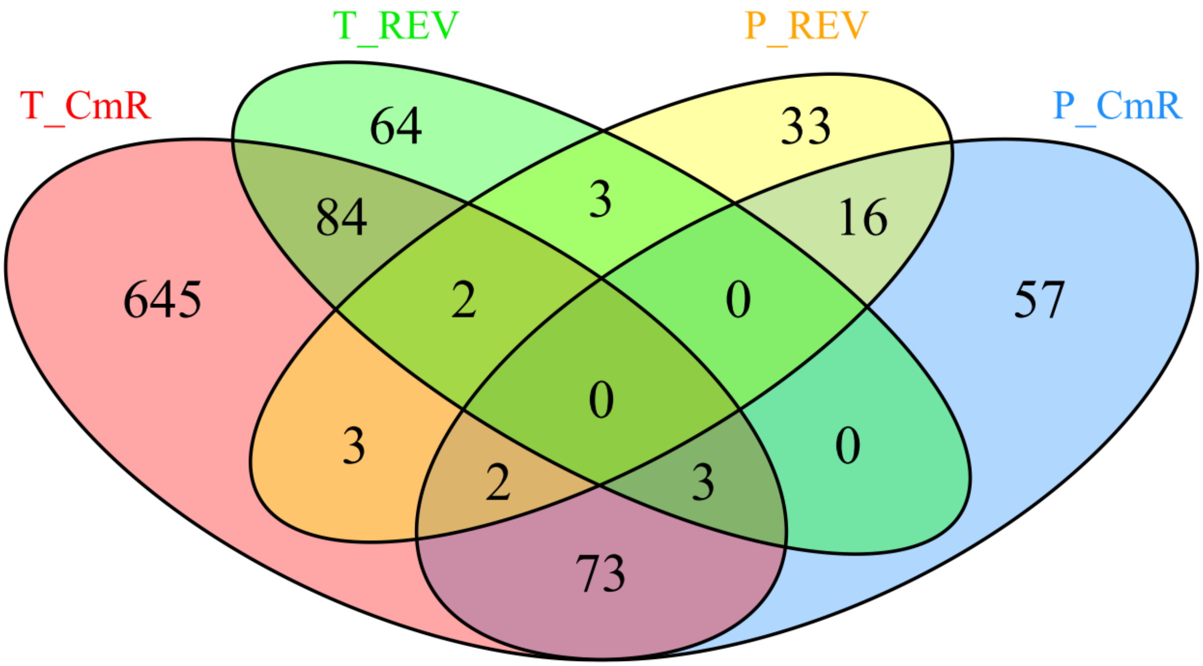

Figure S5: Venn Diagram of whole-transcriptome and whole-proteome data of *S. aureus* CmR-02 (CmR) and its revertant 02REV (REV) versus the wild-type *S. aureus* SG511 Berlin. The number of differentially expressed genes (T_CmR and T_REV) or differentially translated proteins (P_CmR and P_REV) is shown in comparison across the different genetic backgrounds (compare Table 3). The threshold value for differential expression/translation was a fold-change of at least two-fold (Log2 Fold change ≥ 1 or ≤ -1) with a significant p value (≤ 0.05). The total number of genes differentially expressed were 812 (CmR-02) and 156 (02REV), and the number of differentially translated genes was 151 (CmR-02) and 59 (02REV).

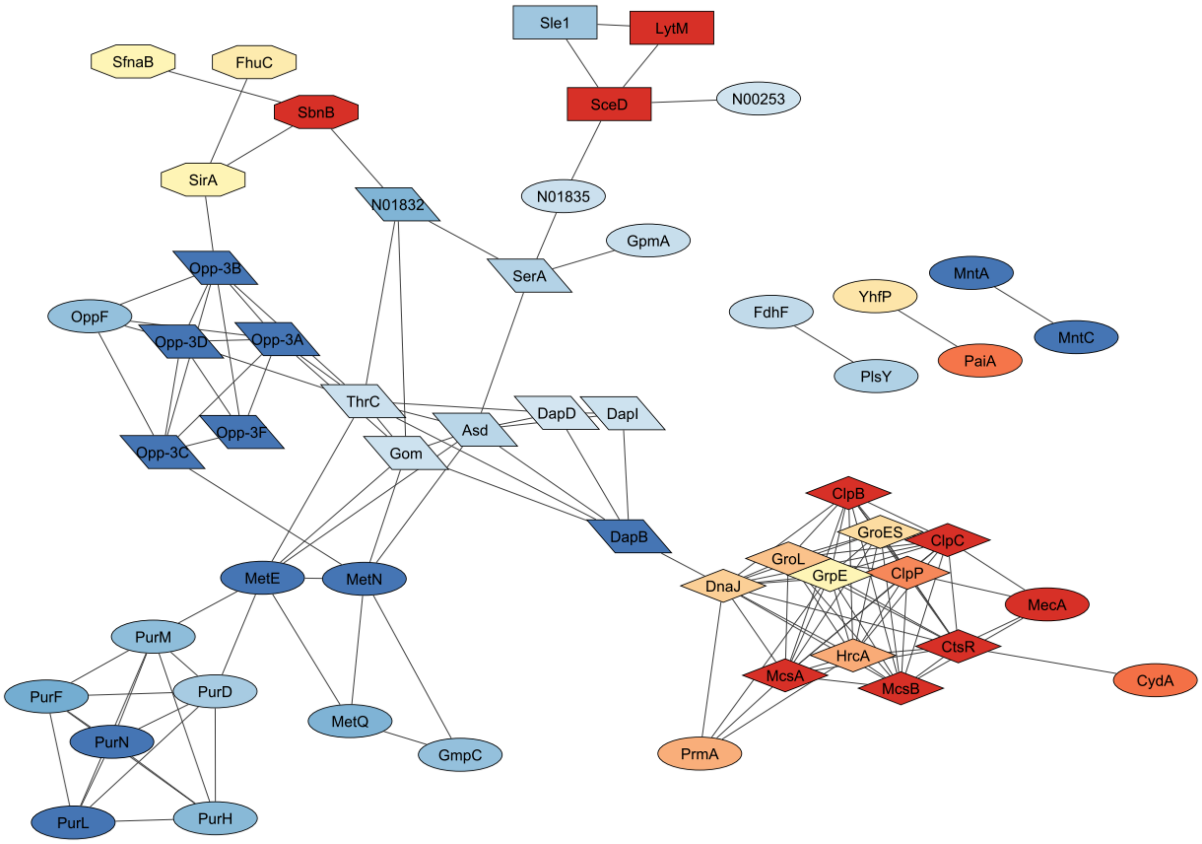

Figure S6: Protein interactome network *S. aureus* CmR-02 for proteins with significantly higher (Log2 FC ≥ 1, *p* value ≤ 0.05, yellow to red color) and lower abundance (Log2 FC ≤ −1, blue color), created using Cytoscape v3.8.2 software (https://cytoscape.org/, last access: 08.07.2022). Depicted proteins also showed differential expression on the transcriptome level. Proteins without differentially expressed interaction partner are not displayed. Medium-confidence interactions (≥ 0.4) in STRING software were selected for this study. The final network for the global protein repository is composed of 54 nodes (proteins) and 141 edges (interactions). Conjunction to a regulon is marked by the border shape: CodY parallelogram, CtsR diamond, Fur octagon, WalR rectangle; N00253 refers to SAOUHSC_00253.

Table S1: Minimal bactericidal concentrations (µg/mL) for cervimycin C (CmC) and D (CmD); n.d., not determined.

Strain	CmC	CmD
Bacillus subtilis 168	0.25	1
Enterococcus faecium BM 4147 (vancomycin-resistant)	4	16
Listeria welshimeri DSM 20650	n.d.	8
Micrococcus luteus ATCC4698	1	2
Mycobacterium smegmatis	>64	> 64
Staphylococcus aureus N315 (methicillin-resistant)	16	> 64
Staphylococcus simulans 22	16	> 64

Table S2: Bacterial strains used in this study.

Strain	Description	Source
Staphylococci		
Staphylococcus aureus		
**SG511 Berlin**	Antibiotic-susceptible strain (CP076660, locus tag prefix KQU62_)	RKI Berlin, Germany
**CmR-01 to CmR-09**	Cervimycin-resistant derivatives of *S. aureus* SG511 Berlin; generated by serial passaging	This study
**01REV to 09REV**	Cervimycin-susceptible revertants of CmR-01 to CmR-09; generated by passaging without antibiotic	This study
**SG511 ClpC^{T215I}**	Allelic exchange mutant of *S. aureus* SG511 Berlin; introduced SNP in *clpC* gene causes amino acid exchange T215I	This study
**SG511 ClpP^{I29F}**	Allelic exchange mutant of *S. aureus* SG511 Berlin; introduced SNP in *clpP* gene causes amino acid exchange I29F	This study
**SG511 DnaK^{A112P}**	Allelic exchange mutant of *S. aureus* SG511 Berlin; introduced SNP in *dnaK* gene causes amino acid exchange A112P	This study
**WalK^{A243V}**	Allelic exchange mutant of *S. aureus* CmR-01; introduced SNP in *clpP* gene reverted amino acid exchange from F to I at position 29, like in the wild-type strain; mutation in *walK* gene was maintained	This study
**SA113 Δatl**	Derivative of *S. aureus* SA113; spectinomycin-resistant;	2
**N315**	Hospital-acquired MRSA; first isolated in 1982 in Japan	3
**NCTC 8325-4**	Laboratory strain; derivative of NCTC 8325; *rsbU*-, *agr*-, UV-cured of Φ11, Φ12, Φ13 prophages	4
**NCTC 8325-4 ΔclpP**	Derivative of NCTC 8325-4 with a complete deletion of the *clpP* gene	5

Strain	Description	Source
**RN4220**	Laboratory strain; derivative of NCTC 8325-4; *rsbU*-, *agr*-, UV-cured of Φ11, Φ12, Φ13 prophages; restriction-negative	6
**USA300 JE2**	Community-acquired MRSA; derivative of *S. aureus* USA300 LAC; cured of plasmids	7
**USA300 JE2 ΔclpP (KB1399)**	Derivative of *S. aureus* USA300 JE2; disruption of *clpP* gene by erythromycin resistance-coding transposon	8
Staphylococcus haemolyticus		
***S. haemolyticus* 655-2**	Vancomycin-susceptible	R. Schwalbe, Baltimore, USA
***S. haemolyticus* 655-2 R16**	Vancomycin-resistant	R. Schwalbe, Baltimore, USA
***S. haemolyticus* 655-2 R32**	Vancomycin-resistant	R. Schwalbe, Baltimore, USA
Staphylococcus simulans		
***S. simulans* 22**	formerly *S. cohnii* 22	9
Bacillus subtilis		
***B. subtilis* 168**	*trpC2*	10; 11
***B. subtilis* 168 ΔclpC (QPB418)**	Derivative of *B. subtilis* 168; deletion of *clpC*; tetracycline-resistant	12
***B. subtilis* 168 ΔclpP (QB4916)**	Derivative of *B. subtilis* 168; deletion of *clpP*; spectinomycin-resistant	13
***B. subtilis* 168 ΔclpX (QB4933)**	Derivative of *B. subtilis* 168; pMutin4-vector integrated into *clpX*	14
B. subtilis 1S34	Derivative of *B. subtilis* 168; non-spore-forming	15
***B. subtilis* JH642**	*trpC2 phe-1*	16
***B. subtilis* JH642 Δspx (ORB3834)**	Derivative of *B. subtilis* JH642; deletion of *spx* (*yjbD*); neomycin-resistant	17
***B. subtilis* JH642 Δspx ΔclpP (ORB3839)**	Derivative of *B. subtilis* ORB3834; deletion of *spx* (*yjbD*); deletion of *clpP*; neomycin and erythromycin-resistant	17
**Enterococci**		

Strain	Description	Source
***Enterococcus faecium* BM 4147**	Clinical isolate; Plasmid pIP816 (Tra^-); vancomycin/ penicillin/ tetracycline-resistant	18
***E. faecium* BM 4147-1**	Plasmid cured derivative of *E. faecium* BM 4147; vancomycin/ penicillin/ tetracycline-susceptible	18
***Escherichia coli* MB5746**	*leu thr lac* (*thi*) *galK*; *lpxC*, *tolC*::Tn*10* (→ defect in the outer membrane)	19
***E. coli* BL21 LOBSTR**	Derived from *E. coli* BL21(DE3); modified copies of *arnA* and *slyD*	20
***E. coli* C43(DE3)**	Derived from *E. coli* BL21(DE3); for expression of toxic membrane proteins	21
**Other**		
***Corynebacterium xerosis* VA 167198**	Clinical isolate	Lab stock
***Listeria welshimeri* DSM 20650**	Isolated from decaying plant material	German collection of Microorganisms and cell cultures GmbH
Micrococcus luteus	Susceptible indicator strain; ATCC4698	American Type Culture Collection, Rockville, Md
***Mycobacterium smegmatis* mc(2)155**	ATCC 70084; Derived from existing strain, -mc(2)154, grown in the absence of kanamycin	American Type Culture Collection, Rockville, Md
***Neisseria sicca* Ku**	Clinical isolate	Lab stock
***Streptococcus agalactiae* B Ku**	Human isolate	Lab stock
***Streptococcus pyogenes* Ku**	Human isolate	Lab stock
**Plasmids**		
**pEPSA5**	Down-regulation of *dnaK*, *pbp3*, *smc*, *ftsZ*, *walK*, *walR*/ over-expression *of ssaA*, *lytM*, *walR*, $walR^C$ in host *S. aureus* RN4220	22; and this study
**pET22b-clpX**	Overexpression construct for staphylococcal ClpX ATPase; from *S. aureus* NCTC8325	23

Strain	Description	Source
**pET22bΔpelB**	Overexpression construct for staphylococcal WalK, and ClpP; from *S. aureus* SG511 and CmR strains	this study
**pREP4groESL(MT)**	Plasmid encoding *E. coli* JM109 GroESL chaperones; facilitates folding and avoids formation of inclusion bodies during protein over-expression	24

Table S3: Oligonucleotide primers used in this study.

Primer	Sequence (5'→3')	T_{An}	Reference
**a. Oligonucleotides for sequencing of *S. aureus* mutants:**			
clpC-for	CGTGTTTTTCGCTCGCTAGG	64.5 °C	this work
clpC-rev	ACACTCTCGGCGTTGTTTCA	64.5 °C	this work
clpC-del-for	AGCTGAGAGTGAGGTGCAAC	58.0 °C	this work
clpC-del-rev	AACCAGTTGGACTTGGTGCA	58.0 °C	this work
clpP-for	ACAAAGGAAAGAGACAAGCGT	64.0 °C	this work
clpP-rev	TCCTTCAAGCCCTAACTGTTT	64.0 °C	this work
dnaK-for	GCCAAAGCCAATGTTCTATTGAC	69.0 °C	this work
dnaK-innen	CAACTAAAGATGCTGGTAAAATT	69.0 °C	this work
dnaK-rev	TCTGGCGAAATCACTCAAGAAC	69.0 °C	this work
walK-for	GGGGCTATGATTACTTTGGCG	64.1 °C	this work
vicK innen	GCTTTAGCATTTAATAACTTGTCTA	ND	24
walK-rev	TGGCTGTCATAGGTGTCGTT	64.1 °C	this work
**b. Oligonucleotides for cloning of pMAD-constructs:**			
pMAD-clpC-for	TTCGGATCCATGAAGGTGTTGCAGCAAGAG (BamHI)	68.0 °C	this work
pMAD-clpC-rev	GTAGAATTCCACGTGCTAATG (EcoRI)	68.0 °C	this work
pMAD-clpP-for	TGTGGATCCCAAATCACTGTACTATGAGAAG (BamHI)	68.0 °C	this work
pMAD-clpP-rev	AACGAATTCCGTTTACCTTTAGCACCAG (EcoRI)	68.0 °C	this work
pMAD-dnaK-for	GCCGGATCCGTTGTCACCGGCTGTTGATAG (BamHI)	68.0 °C	this work
pMAD-dnaK-rev	AAAGAATTCCAAAACCCTGAAGGTTCACG (EcoRI)	68.0 °C	this work
**c. Oligonucleotides for cloning of pET22b-clpX:**			
clpX-for	AAACTCGAGAGCTGATGTTTTACTATTAT	60.5 °C	this work
clpX-rev	AAACATATGTTTAAATTCAATGAAGATGA	60.5 °C	this work

Primer	Sequence (5'→3')	T_{An}	Reference
**d. Oligonucleotides for cloning of pET22bΔpelB-constructs:**			
clpP-NcoI-for	GGAGGCCATGGAGATGAACTTAATTCCTACAG (NcoI)	60.0 °C	this work
clpP-XhoI-rev	TTTCTCGAGCTTTGTTTCAGGTACCATC (XhoI)	60.0 °C	this work
vicK-For	GGTCCATGGGAATGAAGTGGCTAAA (NcoI)	65.5 °C	24
vicK-Rev	TCCCTCGAGTTCATCCCAATCACCG (XhoI)	65.5 °C	24
**e. Oligonucleotides for cloning of pEPSA5-constructs:**			
lytM-for	ATTGAATTCGTATGTGCAGTACAG (EcoRI)	59.0 °C	this work
lytM-rev	TTTTCTAGATTATCTACTTTGCAAGTATG (XbaI)	59.0 °C	this work
ssaA-for	AGAGAATTCATTTTTAGGAGGATATTTTT	61.3 °C	this work
ssaA-rev	GCCTCTAGAAATTAGTGAATGAAGTTATA	61.3 °C	this work
**f. Oligonucleotides for QuikChange Lightning PCR:**			
QCL-ssaA-for	CAAACAGCTACCGTACTGGTAGTTTAGGTGCAAGCTACAGCACTTCAAGC	60.0 °C	this work
QCL-ssaA-rev	CTTGAAGTGCTGTAGCTTGCACCTAAACTACCAGTACGGTAGCTGTTTG	60.0 °C	this work

Table S4: Complete list of cervimycin-resistant *S. aureus* mutants and the respective revertants generated in this study. Sequence comparisons revealed a combination of amino acid exchanges in the protease ClpP or the cognate Clp ATPase ClpC, and the essential histidine kinase WalK and in one case the cognate response regulator WalR.

**Strain**	**MIC CmC**	***clpP***	**ClpP**	***walK***	**WalK**
CmR-01	128	A →T	I29F	C →T	A243V
CmR-02*	128	A →T	I29F	C →T	A243V
CmR-03	128	A →T	I29F	C →T	A243V
01REV	2	A →T; G → C	I29F; M31I	C →T; C →T	A243V; S191L
02REV	2	A →T; G → C	I29F; M31I	C →T; C →T	A243V; S191L
03REV	2	A →G	I29V	C →T; C →T	A243V; S191L
	**MIC CmC**	***clpC***	**ClpC**	***walK***	**WalK**
CmR-04	128	4,561 bp Del.	Deletion	ΔCAA	ΔQ371
CmR-05	128	C →T	T215I	C →T	A243V
CmR-06	128	C →T	T215I	C →T	A243V
04REV	32	4,561 bp Del.	Deletion	ΔCAA; G →A	ΔQ371, A554T
05REV	16	C →T	T215I	C →T; C →T	A243V, T217M
06REV	32	C →T	T215I	C →T; G →A	A243V, R557H
	**MIC CmD**	***clpC***	**ClpC**	***walK***	**WalK**
CmR-07	107.5	C →A	P204H	T → G	Y549D
CmR-08	107.5	C →A	P204H	T → G	Y549D
CmR-09	107.5	C →A	P204H	T → G	Y549D
07REV	> 64	C →A	P204H	T → G; *walR*: G →T	Y549D, WalR: M57I
08REV	32	C →A	P204H	T → G; C →T	Y549D, S393F
09REV	16	C →A	P204H	T → G; G →A	Y549D, R555H

*CmR-02 harbors an additional mutation in the heat shock protein gene *dnaK* (G → C), leading to the amino acid exchange A112P.

Table S5: Transcriptomic and proteomic data of *S. aureus* CmR-02 and its revertant 02REV. Genes/ proteins with at least 2-fold differential expression in comparison to the wild-type *S. aureus* SG511 and significant p-value ($p \leq 0.05$) are shown, which corresponds to a Log2 Fold Change ≥ 1 or ≤ -1. The specified regulators correspond to the genome of *S. aureus* NCTC 8325 and were derived from the Aureowiki database (25). Significantly differentially expressed genes/ proteins are marked in blue (down-regulated) and red (up-regulated), respectively. 0 indicates a non-significant expression/ detection level.

						Transcriptome (Log2 Fold Change)		Proteome (Log2 Fold Change)	
**Locus SG511**	**Gene Name**	**Regulator 1**	**Regulator 2**	**Regulator 3**	**Regulator 4**	**CmR-02**	**02REV**	**CmR-02**	**02REV**
KQU62_00025	-	0	0	0	0	-1.885	0	0	0
KQU62_00035	-	0	0	0	0	-1.845	0	0	0
KQU62_00045	-	0	0	0	0	-1.958	0	0	0
KQU62_00050	-	0	0	0	0	-2.589	0	0	0
KQU62_00065	-	0	0	0	0	-2.785	0	-0.518	-0.576
KQU62_00075	-	SigB	0	0	0	-1.195	-0.929	0	0
KQU62_00090	psuG	CcpA	0	0	0	-1.111	0	0	0
KQU62_00100	nanT	NanR	CcpA	0	0	-3.997	-2.172	0	0
KQU62_00105	nanA	NanR	CcpA	0	0	-5.419	-2.733	0.663	0
KQU62_00110	nanK	NanR	CcpA	0	0	-2.209	-1.281	0	0
KQU62_00115	nanR	NanR	0	0	0	0.451	-0.492	1.58	0
KQU62_00120	nanE	NanR	0	0	0	-1.672	-1.275	0	0
KQU62_00125	-	HutR	0	0	0	-1.615	0	0	0
KQU62_00130	geh	0	0	0	0	-1.396	0.633	0	0
KQU62_00135	-	0	0	0	0	1.039	-0.687	0	0
KQU62_00140	yqiG	0	0	0	0	1.266	0	0	0
KQU62_00150	-	0	0	0	0	2.107	0	0	0
KQU62_00155	gcvH-L	0	0	0	0	1.679	0	0	0

						Transcriptome (Log2 Fold Change)		Proteome (Log2 Fold Change)	
Locus SG511	Gene Name	Regulator 1	Regulator 2	Regulator 3	Regulator 4	CmR-02	02REV	CmR-02	02REV
KQU62_00160	-	0	0	0	0	1.985	0	0	0
KQU62_00165	sirTM	0	0	0	0	1.971	0	0	0
KQU62_00170	lplA2	0	0	0	0	1.943	0	0	0
KQU62_00175	-	SigB	0	0	0	1.568	-0.368	0.78	0
KQU62_00215	glpT	CcpA	0	0	0	-1.384	0.592	0	0
KQU62_00220	catE	SigB	0	0	0	-1.164	0	0	0
KQU62_00260	tatC	Fur	0	0	0	1.824	0	0	0
KQU62_00265	tatA	Fur	0	0	0	2.473	0	0	0
KQU62_00270	-	0	0	0	0	2.663	0	0	0
KQU62_00275	-	0	0	0	0	-1.143	-0.869	0	0
KQU62_00310	metE	0	0	0	0	-1.619	0	-4.14	0
KQU62_00315	metF	T-box	0	0	0	-1.937	0	0	0
KQU62_00320	metC	T-box	0	0	0	-2.239	-0.311	0	0
KQU62_00325	metI	T-box	0	0	0	-1.947	0	0	0
KQU62_00370	-	0	0	0	0	1.229	0.379	0	0
KQU62_00410	-	0	0	0	0	1.255	0	0	0
KQU62_00475	-	0	0	0	0	-1.297	0.431	0	0
KQU62_00480	-	0	0	0	0	-1.299	0	0	0
KQU62_00485	-	0	0	0	0	-1.253	0	0	0
KQU62_00490	-	0	0	0	0	-1.459	-0.267	0	0
KQU62_00530	-	0	0	0	0	-1.353	0	0	0
KQU62_00545	-	0	0	0	0	1.011	0	0.389	-0.411
KQU62_00575	nfrA	CodY	0	0	0	1.01	-0.492	0.67	0
KQU62_00580	tcyP	CodY	CymR	0	0	-2.589	-0.53	-1.75	0.336
KQU62_00605	xpt	0	0	0	0	-1.079	0	-0.988	0
KQU62_00610	pbuX	G-box	0	0	0	-1.023	-0.432	0	0
KQU62_00615	guaB	0	0	0	0	-1.163	-0.556	-0.987	-0.291

Locus SG511	Gene Name	Regulator 1	Regulator 2	Regulator 3	Regulator 4	Transcriptome (Log2 Fold Change)		Proteome (Log2 Fold Change)	
						CmR-02	02REV	CmR-02	02REV
KQU62_00620	guaA	0	0	0	0	-1.134	-0.636	-0.852	0
KQU62_00635	-	0	0	0	0	0	1.79	0	0
KQU62_00650	-	0	0	0	0	0.889	-0.629	1.06	0
KQU62_00660	ssl2	0	0	0	0	1.481	0	0	0
KQU62_00695	hsdS1	0	0	0	0	-0.944	-0.549	-1.88	0
KQU62_00710	SPIN	0	0	0	0	-2.335	-0.734	0	0
KQU62_00715	lpl5	0	0	0	0	0	1.04	0	0
KQU62_00765	-	0	0	0	0	-1.306	0	0	0
KQU62_00770	yciC	0	0	0	0	1.032	0	0	0
KQU62_00795	mpsA	0	0	0	0	-1.319	-0.856	0	0
KQU62_00800	mpsB	0	0	0	0	-1.128	-0.455	0	0
KQU62_00810	-	0	0	0	0	-1.621	0	0	0
KQU62_00830	-	HisR	0	0	0	1.616	0.501	0	0
KQU62_00845	metN1	CymR	0	0	0	-2.11	0	0	0
KQU62_00850	metP2	CymR	0	0	0	-2.653	0	0	0
KQU62_00855	gmpC	CymR	0	0	0	-2.214	-0.344	-1.67	0.574
KQU62_00860	sle1	WalR	0	0	0	-1.657	1.006	-1.56	1.25
KQU62_00865	-	0	0	0	0	1.001	0	0	0
KQU62_00870	-	0	0	0	0	-1.051	0	0.723	1.58
KQU62_00890	gltC	GltC	CodY	0	0	1.136	0.801	0.907	0
KQU62_00895	gltB	GltC	CodY	0	0	-1.223	0	0	0
KQU62_00910	treP	TreR	CcpA	0	0	-2.516	0	-0.37	0
KQU62_00915	treC	TreR	CcpA	0	0	-1.012	0	0	0
KQU62_00935	-	0	0	0	0	-0.24	0	0	1.86
KQU62_00975	ldcC	0	0	0	0	-1.219	-0.323	0	0
KQU62_01045	ispE	0	0	0	0	-1.343	0.407	0	0
KQU62_01050	purR	0	0	0	0	-1.293	0	-0.546	0

Locus SG511	Gene Name	Regulator 1	Regulator 2	Regulator 3	Regulator 4	Transcriptome (Log2 Fold Change)		Proteome (Log2 Fold Change)	
						CmR-02	02REV	CmR-02	02REV
KQU62_01055	yabJ	SigB	0	0	0	-1.257	0	0	0
KQU62_01060	spoVG	SigB	0	0	0	-1.112	-0.298	0	0
KQU62_01130	ftsH	0	0	0	0	1.102	0.264	0.313	0.248
KQU62_01235	pdxR	0	0	0	0	1.18	-0.469	0	0
KQU62_01250	nupC	CcpA	0	0	0	-1.037	0.298	-0.614	0.467
KQU62_01255	ctsR	CtsR	0	0	0	3.101	-0.482	4.74	0
KQU62_01260	mcsA	CtsR	0	0	0	3.469	-0.496	4.59	0.507
KQU62_01265	mcsB	CtsR	0	0	0	3.692	-0.343	4.59	2.13
KQU62_01270	clpC	CtsR	0	0	0	3.533	-0.293	2.92	0.679
KQU62_01280	-	0	0	0	0	0	0	0	2.41
KQU62_01335	rplK	0	0	0	0	1.092	0.453	0	0
KQU62_01340	rplA	0	0	0	0	1.125	0	0	0
KQU62_01415	-	CcpA	0	0	0	-1.844	0	-0.325	0
KQU62_01435	dgk	0	0	0	0	0.589	0.74	0	2.15
KQU62_01455	sdrC	0	0	0	0	-1.548	0.344	0	0
KQU62_01460	sdrD	0	0	0	0	-2.023	-0.421	-0.82	-0.725
KQU62_01475	-	0	0	0	0	-1.161	-0.404	0	-0.257
KQU62_01500	hxlA	SigB	0	0	0	2.303	-0.549	1.11	-0.513
KQU62_01505	hxlB	SigB	0	0	0	2.135	-0.876	0.915	-0.494
KQU62_01510	-	0	0	0	0	2.34	0.904	0	0
KQU62_01540	vraX	0	0	0	0	-2.869	0.99	0	0
KQU62_01590	lipL	0	0	0	0	-0.961	-0.39	-1.07	0
KQU62_01595	mvk	SigB	0	0	0	1.058	0.48	0.291	0
KQU62_01630	-	0	0	0	0	-1.479	-0.31	-1.76	0
KQU62_01635	-	0	0	0	0	-1.532	0	0	0
KQU62_01640	IS1182	0	0	0	0	-1.432	-0.338	0	0
KQU62_01645	-	0	0	0	0	-1.5	-0.289	0	0

Locus SG511	Gene Name	Regulator 1	Regulator 2	Regulator 3	Regulator 4	Transcriptome (Log2 Fold Change)		Proteome (Log2 Fold Change)	
						CmR-02	02REV	CmR-02	02REV
KQU62_01650	-	0	0	0	0	-1.443	0	0	0
KQU62_01680	adhP	Rex	0	0	0	-3.656	-1.004	-0.404	-0.572
KQU62_01685	-	0	0	0	0	-2.923	0	0	0
KQU62_01690	-	0	0	0	0	-0.89	0	-1.07	0
KQU62_01725	-	0	0	0	0	-0.977	-1.239	-0.664	-0.834
KQU62_01735	-	SigB	0	0	0	0.689	-0.582	0	-1.24
KQU62_01760	-	SigB	0	0	0	2.043	1.354	0	0
KQU62_01815	mntC	MntR	0	0	0	-5.057	-1.214	-2.08	0
KQU62_01820	mntB	MntR	0	0	0	-5.038	0	0	0
KQU62_01825	mntA	MntR	0	0	0	-4.762	0	-2.12	0
KQU62_01880	nupG	0	0	0	0	-1.021	0.18	-1.16	0.473
KQU62_01895	fhuC	Fur	0	0	0	2.643	0.471	1.14	0.53
KQU62_01900	fhuB	Fur	0	0	0	2.145	0	0	0
KQU62_01905	fhuG	Fur	0	0	0	1.34	0	0	0
KQU62_01925	-	0	0	0	0	0	0	2.95	-1.09
KQU62_01930	-	0	0	0	0	2.032	0	0	0
KQU62_01935	-	0	0	0	0	1.509	0	0	0
KQU62_01965	vraF	GraR	0	0	0	0.261	0	0.845	1.12
KQU62_01975	pitR	GraR	0	0	0	1.12	0.383	0	0.185
KQU62_02000	sarX	0	0	0	0	-2.264	-0.484	0	-0.8
KQU62_02055	-	0	0	0	0	1.975	0	0	0
KQU62_02060	-	SigB	0	0	0	1.305	0	0	-0.37
KQU62_02085	uppP	0	0	0	0	1.101	0.297	0.795	0
KQU62_02100	mgrA	RNAIII	0	0	0	-1.287	-0.305	0	0
KQU62_02120	-	0	0	0	0	-1.867	-0.632	0	0
KQU62_02125	phrB	0	0	0	0	-1.465	-0.489	0	0
KQU62_02130	-	0	0	0	0	1.375	0	0	0

Locus SG511	Gene Name	Regulator 1	Regulator 2	Regulator 3	Regulator 4	Transcriptome (Log2 Fold Change) CmR-02	Transcriptome (Log2 Fold Change) 02REV	Proteome (Log2 Fold Change) CmR-02	Proteome (Log2 Fold Change) 02REV
KQU62_02150	-	0	0	0	0	0.569	1.503	0	0
KQU62_02160	fruR	FruR	CcpA	0	0	-1.535	0	0	0
KQU62_02165	pfkB	FruR	CcpA	0	0	-1.455	0	-0.405	0
KQU62_02170	fruA	FruR	CcpA	0	0	-1.378	0	-0.567	0
KQU62_02175	nagA	0	0	0	0	1.107	0	0.457	-0.43
KQU62_02185	-	0	0	0	0	1.509	-0.29	0.639	0
KQU62_02190	csbB	0	0	0	0	1.269	-0.185	0	0
KQU62_02215	-	0	0	0	0	1.29	0.577	0	0
KQU62_02280	opuBA	0	0	0	0	1.502	0	0.904	0
KQU62_02285	opuBB	0	0	0	0	1.177	-0.45	0.464	-0.417
KQU62_02290	hisC	0	0	0	0	-1.243	-0.193	-0.525	0
KQU62_02310	dtpT	CodY	0	0	0	-1.251	-0.13	-0.857	0.389
KQU62_02325	nrdI	NrdR	0	0	0	0	-0.245	2.35	1.25
KQU62_02340	sstA	0	0	0	0	2.546	0	0	0
KQU62_02345	sstB	0	0	0	0	2.892	0	0	0
KQU62_02350	sstC	0	0	0	0	3.271	0	0	0
KQU62_02355	sstD	0	0	0	0	2.214	-0.668	0.67	0.279
KQU62_02435	raiA	CodY	0	0	0	-2.536	-1.347	0	-0.297
KQU62_02440	secA	0	0	0	0	1.189	0	0.499	0
KQU62_02445	-	0	0	0	0	1.214	0	0	0
KQU62_02460	-	0	0	0	0	1.124	0	0.292	-0.351
KQU62_02465	csbA	0	0	0	0	1.234	0	0	0
KQU62_02515	whiA	SigB	0	0	0	0	0	1.95	1.1
KQU62_02520	clpP	CtsR	0	0	0	2.796	-0.728	1.82	0.352
KQU62_02525	-	0	0	0	0	0	-1.407	0	0
KQU62_02540	gapR	GapR	SigB	0	0	-1.444	0	0	0
KQU62_02545	gap	GapR	0	0	0	-1.434	-0.656	0	0

Locus SG511	Gene Name	Regulator 1	Regulator 2	Regulator 3	Regulator 4	Transcriptome (Log2 Fold Change)		Proteome (Log2 Fold Change)	
						CmR-02	02REV	CmR-02	02REV
KQU62_02550	pgk	GapR	0	0	0	-1.728	-0.47	-0.189	-0.197
KQU62_02555	tpiA	GapR	0	0	0	-1.63	-0.476	0	0
KQU62_02560	pgm	GapR	0	0	0	-1.368	0	0	0
KQU62_02600	-	0	0	0	0	0.153	-14.247	0	0
KQU62_02605	integrase	0	0	0	0	0.396	-12.883	0	0
KQU62_02610	-	0	0	0	0	0	-5.04	0	0
KQU62_02620	-	0	0	0	0	0	-7.259	0	0
KQU62_02625	-	0	0	0	0	0	-6.503	0	0
KQU62_02630	-	0	0	0	0	0	-7.986	0	0
KQU62_02635	-	0	0	0	0	0.533	-5.974	0	0
KQU62_02640	-	0	0	0	0	0	-8.093	0	0
KQU62_02645	-	0	0	0	0	0	-8.717	0	0
KQU62_02650	-	0	0	0	0	0	-8.853	0	0
KQU62_02655	-	0	0	0	0	-1.801	-9.655	0	0
KQU62_02660	-	0	0	0	0	-0.676	-9.728	0	0
KQU62_02670	-	0	0	0	0	0	-7.92	0	0
KQU62_02675	-	0	0	0	0	0	-8.683	0	0
KQU62_02680	-	0	0	0	0	0	-7.684	0	0
KQU62_02685	tst	0	0	0	0	0	-6.767	0	0
KQU62_02690	-	0	0	0	0	-1.207	-8.932	0	0
KQU62_02695	sec3	0	0	0	0	-0.843	-10.278	0	0
KQU62_02700	sel	0	0	0	0	-3.701	-15.267	0	0
KQU62_02710	-	0	0	0	0	-2.684	-0.435	-1.16	0
KQU62_02725	-	SigB	0	0	0	0	-0.6	9.14	0
KQU62_02730	clfA	SigB	0	0	0	-1.111	-0.561	0	0
KQU62_02740	emp	0	0	0	0	-1.469	-0.751	0	0
KQU62_02760	-	SigB	0	0	0	1.147	0	0	0

Locus SG511	Gene Name	Regulator 1	Regulator 2	Regulator 3	Regulator 4	Transcriptome (Log2 Fold Change)		Proteome (Log2 Fold Change)	
						CmR-02	02REV	CmR-02	02REV
KQU62_02765	-	SigB	0	0	0	1.33	0	0	0
KQU62_02770	InsA	0	0	0	0	1.692	0.519	0	0
KQU62_02800	lysE	CodY	0	0	0	-1.757	0.44	0	0
KQU62_02805	-	0	0	0	0	-1.372	0	0	0
KQU62_02815	aroD	0	0	0	0	1.081	0.206	0.535	0
KQU62_02820	-	Fur	0	0	0	1.477	-0.195	0	-0.518
KQU62_02825	-	0	0	0	0	1.123	0	0.448	0
KQU62_02855	metN	S-box	0	0	0	-2.216	-0.595	-2.59	0.416
KQU62_02860	metI2	S-box	0	0	0	-2.584	-0.524	0	0
KQU62_02865	metQ	S-box	0	0	0	-2.695	-0.532	-1.89	0.215
KQU62_02870	-	SigB	0	0	0	-1.082	-1.074	0.769	0
KQU62_02875	-	0	0	0	0	1.745	0.167	0	0
KQU62_02905	-	0	0	0	0	1.616	0	0	0
KQU62_02910	-	0	0	0	0	1.136	0	0	0
KQU62_02915	mpfB	SigB	0	0	0	1.267	0.361	0	0
KQU62_02920	fabK	SigB	0	0	0	1.249	0	0	0
KQU62_02950	-	0	0	0	0	1.074	0	0	0
KQU62_02955	-	0	0	0	0	1.066	0.478	0	0
KQU62_02970	-	GraR	0	0	0	1.062	0.609	0	0
KQU62_02985	dltB	0	0	0	0	-0.173	0	-1.76	0
KQU62_03000	nfu	0	0	0	0	1.387	0.277	0	0
KQU62_03035	nhaC	HisR	0	0	0	1.172	0.736	0	0
KQU62_03050	mnhG	0	0	0	0	-0.498	0	1.7	2.03
KQU62_03095	-	0	0	0	0	-1.322	-0.156	0	0
KQU62_03100	namA	0	0	0	0	1.637	-0.955	1.77	-0.453
KQU62_03105	rocD	ArgR	CcpA	0	0	-1.71	0	0.248	0.541
KQU62_03110	gudB	CcpA	0	0	0	-1.387	0	0	-0.307

Locus SG511	Gene Name	Regulator 1	Regulator 2	Regulator 3	Regulator 4	Transcriptome (Log2 Fold Change) CmR-02	Transcriptome (Log2 Fold Change) 02REV	Proteome (Log2 Fold Change) CmR-02	Proteome (Log2 Fold Change) 02REV
KQU62_03115	glpQ	0	0	0	0	-1.95	0.369	0	0
KQU62_03120	argH	ArgR	CodY	0	0	-4.094	-1.656	0	0
KQU62_03125	argG	CodY	ArgR	0	0	-4.139	-1.521	0	-0.593
KQU62_03165	-	0	0	0	0	1.868	0.844	0	0
KQU62_03180	-	0	0	0	0	1.026	0	0.344	0
KQU62_03185	sufT	0	0	0	0	1.717	0.169	0	0
KQU62_03195	clpB	CtsR	0	0	0	4.281	-0.621	4.01	1.46
KQU62_03205	leuA2	0	0	0	0	1.561	0	0	0
KQU62_03215	-	0	0	0	0	1.176	1.5	0	0
KQU62_03245	-	0	0	0	0	-1.381	0.514	0	0
KQU62_03250	opp-3B	CodY	0	0	0	-3.89	0.293	-4.45	0
KQU62_03255	opp-3C	CodY	0	0	0	-4.317	0	-3.62	0
KQU62_03260	opp-3D	CodY	0	0	0	-4.411	0	-4.51	0.666
KQU62_03265	opp-3F	CodY	0	0	0	-4.441	0	-3.03	0.725
KQU62_03270	opp-3A	CodY	0	0	0	-4.385	0	-5.71	0.635
KQU62_03275	opp-4A	CodY	0	0	0	-4.767	0	0	0
KQU62_03325	spxA	0	0	0	0	-1.43	-0.451	2.43	-2.67
KQU62_03330	mecA/trfA	0	0	0	0	1.17	0	2.19	0.388
KQU62_03340	pepF	0	0	0	0	1.133	0	0.433	-0.304
KQU62_03345	yjbH	0	0	0	0	0.866	-0.48	2.25	0
KQU62_03360	-	0	0	0	0	1.082	0	-0.71	0
KQU62_03400	agcS	CodY	0	0	0	-1.667	0.24	0	0
KQU62_03405	-	0	0	0	0	-0.726	0.251	-1.11	0
KQU62_03420	-	CcpA	0	0	0	-1.184	0	0	0
KQU62_03485	-	CodY	0	0	0	-2.927	-1.084	0	0
KQU62_03490	lplA1	0	0	0	0	1.869	0	0.701	0
KQU62_03495	-	0	0	0	0	1.545	0.301	0	0

						Transcriptome (Log2 Fold Change)		Proteome (Log2 Fold Change)	
**Locus SG511**	**Gene Name**	**Regulator 1**	**Regulator 2**	**Regulator 3**	**Regulator 4**	**CmR-02**	**02REV**	**CmR-02**	**02REV**
KQU62_03500	-	0	0	0	0	1.476	0.331	0	0
KQU62_03535	-	0	0	0	0	1.901	0	0	0
KQU62_03545	tarM	0	0	0	0	1.021	1.104	0	0
KQU62_03550	-	0	0	0	0	1.15	0.269	0	0
KQU62_03560	-	SigB	0	0	0	1.466	0	0	0
KQU62_03570	-	0	0	0	0	1.158	0	0.501	-0.632
KQU62_03600	-	0	0	0	0	-1.101	-0.816	0	0
KQU62_03620	-	GraR	0	0	0	3.842	0	0	0
KQU62_03625	-	0	0	0	0	-1.323	0	0	0
KQU62_03635	atl	WalR	SigB	0	0	-0.948	0.781	-1.25	0.389
KQU62_03660	qoxD	0	0	0	0	-1.363	0	0	0
KQU62_03665	qoxC	0	0	0	0	-1.487	0	-0.699	0
KQU62_03670	qoxB	0	0	0	0	-1.532	0	-0.645	0
KQU62_03675	qoxA	GraR	0	0	0	-1.587	0	0	0
KQU62_03685	-	0	0	0	0	-2.617	0	0	0
KQU62_03700	purE	0	0	0	0	0	0	-1.16	0
KQU62_03705	purK	0	0	0	0	0	0.398	-1.66	0.614
KQU62_03710	purC	0	0	0	0	-0.699	0	-1.88	0
KQU62_03715	purS	0	0	0	0	-0.718	0.732	-4.98	0
KQU62_03720	purQ	0	0	0	0	-0.931	0.617	-2.01	0
KQU62_03725	purL	0	0	0	0	-1.497	0.261	-2.3	0
KQU62_03730	purF	0	0	0	0	-1.953	0	-2	0
KQU62_03735	purM	0	0	0	0	-2.182	0	-1.73	0
KQU62_03740	purN	0	0	0	0	-2.244	-0.326	-2.49	0
KQU62_03745	purH	0	0	0	0	-2.404	-0.558	-1.8	0
KQU62_03750	purD	0	0	0	0	-2.365	-0.406	-1.48	0
KQU62_03755	thiX	0	0	0	0	-1.497	0	-0.504	0

Locus SG511	Gene Name	Regulator 1	Regulator 2	Regulator 3	Regulator 4	Transcriptome (Log2 Fold Change)		Proteome (Log2 Fold Change)	
						CmR-02	02REV	CmR-02	02REV
KQU62_03760	thiW	0	0	0	0	-1.508	0	-0.735	0
KQU62_03765	thiV	0	0	0	0	-1.385	0	-1.07	0.403
KQU62_03775	-	0	0	0	0	-0.414	0	1.16	1.28
KQU62_03800	nrdH	0	0	0	0	1.078	0.59	0	0
KQU62_03805	cydA	0	0	0	0	1.058	0	1.98	0.415
KQU62_03810	cydB	0	0	0	0	0.987	0	1.85	-1.21
KQU62_03835	def	0	0	0	0	1.35	0.442	0.319	-0.269
KQU62_03840	-	0	0	0	0	1.991	0.262	0.639	0
KQU62_03890	potD	0	0	0	0	0	0.582	0	1.04
KQU62_03900	-	0	0	0	0	0	-0.482	0	1.94
KQU62_03905	mntH	MntR	0	0	0	-1.025	0	0	1.72
KQU62_03910	-	0	0	0	0	1.565	0.502	0	0
KQU62_03925	typA	0	0	0	0	1.163	0.836	0	0.335
KQU62_03945	ftsW	0	0	0	0	1.139	0.4	0	0
KQU62_03950	pycA	CodY	0	0	0	-1.631	-0.271	-0.932	-0.331
KQU62_03955	ctaA	0	0	0	0	-1.767	0	0	0
KQU62_03960	ctaB	0	0	0	0	-1.194	0.487	0	0
KQU62_03965	ctaM	0	0	0	0	-1.438	0	0	0
KQU62_03980	-	0	0	0	0	1.707	0	0	0
KQU62_03985	-	0	0	0	0	1.804	0.847	0	0
KQU62_04005	-	0	0	0	0	1.142	0	0	0
KQU62_04010	ylbN	0	0	0	0	1.337	1.002	1.74	0
KQU62_04020	sasJ	Fur	0	0	0	1.774	0.338	0	0
KQU62_04035	isdD	Fur	0	0	0	1.084	0.734	0	0
KQU62_04060	-	0	0	0	0	3.553	0	0	0
KQU62_04080	rnhC	0	0	0	0	1.691	1.022	0	0
KQU62_04120	sdhC	CcpA	0	0	0	-2.368	0.351	-0.325	0

Locus SG511	Gene Name	Regulator 1	Regulator 2	Regulator 3	Regulator 4	Transcriptome (Log2 Fold Change)		Proteome (Log2 Fold Change)	
						CmR-02	02REV	CmR-02	02REV
KQU62_04125	sdhA	CcpA	0	0	0	-2.327	0.321	-0.512	0.268
KQU62_04130	sdhB	CcpA	0	0	0	-2.353	0.331	-0.573	0.265
KQU62_04170	-	0	0	0	0	-2.161	0	0	0
KQU62_04230	argF	0	0	0	0	-3.24	-1.399	0	-0.817
KQU62_04245	-	0	0	0	0	-1.626	0.348	0	0
KQU62_04250	eta	0	0	0	0	-3.229	-0.429	0	0
KQU62_04255	-	0	0	0	0	0	1.317	0	0
KQU62_04270	yfnB	0	0	0	0	1.312	0	1.06	0
KQU62_04280	bshC	0	0	0	0	1.162	0	0.414	-0.213
KQU62_04315	divIB	0	0	0	0	0	0	2.03	1.81
KQU62_04330	pgeF	0	0	0	0	-0.402	0	-1.37	0
KQU62_04340	sepF	0	0	0	0	0	0	2.8	0
KQU62_04385	pyrR	pyrR leader	0	0	0	0	1.141	0	0
KQU62_04390	uraA	pyrR leader	0	0	0	-0.724	1.256	0	0
KQU62_04395	pyrB	pyrR leader	0	0	0	0	1.015	0	0
KQU62_04460	-	0	0	0	0	-2.899	-0.309	-1.62	0
KQU62_04465	-	SigB	0	0	0	-1.122	-0.752	0	0
KQU62_04515	rpmB	0	0	0	0	-1.902	-0.531	0	0
KQU62_04540	plsX	FapR	0	0	0	1.161	0.33	0.396	0
KQU62_04545	fabD	FapR	0	0	0	1.169	0	0.331	-0.568
KQU62_04550	fabG	FapR	0	0	0	1.052	0	0.284	0
KQU62_04640	dprA	0	0	0	0	0	1.318	0	0
KQU62_04645	topA	0	0	0	0	1.091	0	0	0
KQU62_04650	trmFO	0	0	0	0	1.026	0	0	0
KQU62_04655	xerC	0	0	0	0	-0.634	0	1.14	1
KQU62_04675	-	0	0	0	0	1.037	0.566	0	0
KQU62_04695	pyrH	0	0	0	0	1.029	0.702	-0.231	0

						Transcriptome (Log2 Fold Change)		Proteome (Log2 Fold Change)	
**Locus SG511**	**Gene Name**	**Regulator 1**	**Regulator 2**	**Regulator 3**	**Regulator 4**	**CmR-02**	**02REV**	**CmR-02**	**02REV**
KQU62_04710	cdsA	0	0	0	0	1.036	1.016	0	0
KQU62_04765	ribF	0	0	0	0	1.002	0.144	0.727	0.298
KQU62_04795	-	0	0	0	0	1.112	0.877	-0.239	0
KQU62_04825	cinA	0	0	0	0	1.239	0	0.496	0
KQU62_04840	-	0	0	0	0	1.476	-0.618	2.64	0
KQU62_04940	glnR	GlnR	CodY	0	0	1.863	0	0	0
KQU62_04945	glnA	CodY	GlnR	0	0	1.157	0	0	-0.305
KQU62_04950	-	0	0	0	0	1.932	0.748	0	0
KQU62_04965	-	0	0	0	0	0	1.103	0	0
KQU62_04970	IS30	0	0	0	0	-5.285	-7.22	0	0
KQU62_04975	-	0	0	0	0	-4.674	-2.706	0	-0.519
KQU62_04980	IS30	0	0	0	0	-1.008	0	0	0
KQU62_04985	-	0	0	0	0	0	1.523	0	0
KQU62_05010	-	0	0	0	0	-2.263	0	0	0
KQU62_05015	-	0	0	0	0	-1.718	0	0	0
KQU62_05025	-	0	0	0	0	0	2.089	0	0
KQU62_05035	-	0	0	0	0	-1.844	0	0	0
KQU62_05040	-	0	0	0	0	-2.098	0	0	0
KQU62_05065	-	0	0	0	0	-1.611	-1.051	0	0
KQU62_05070	-	0	0	0	0	-1.402	0	0	0
KQU62_05090	nucI	0	0	0	0	1.629	0.88	0	0
KQU62_05100	-	CcpA	0	0	0	-1.212	0.36	0	0
KQU62_05105	thrD	CodY	0	0	0	-1.244	0.661	0	0
KQU62_05110	hom	CodY	0	0	0	-1.639	0.232	-1.08	0
KQU62_05115	thrC	CodY	0	0	0	-1.985	0	-1.11	0
KQU62_05120	thrB	CodY	0	0	0	-2.042	0.209	0	0
KQU62_05130	-	0	0	0	0	-1.909	-0.537	0	0

Locus SG511	Gene Name	Regulator 1	Regulator 2	Regulator 3	Regulator 4	Transcriptome (Log2 Fold Change)		Proteome (Log2 Fold Change)	
						CmR-02	02REV	CmR-02	02REV
KQU62_05135	-	0	0	0	0	-1.309	0	0	0
KQU62_05140	lysP2	CodY	0	0	0	-1.358	-0.298	-1.1	0.382
KQU62_05160	guaC	0	0	0	0	1.428	0.407	-1.29	0
KQU62_05165	narI2	0	0	0	0	1.36	0.23	0	0
KQU62_05175	-	0	0	0	0	2.778	0	0	0
KQU62_05210	sbcC	LexA	0	0	0	0.584	0.275	1.41	1.68
KQU62_05240	plsY	0	0	0	0	-1.196	0	-1.38	0
KQU62_05300	dinB2	LexA	0	0	0	1.705	0	0	0
KQU62_05395	-	0	0	0	0	1.149	0.684	0	0
KQU62_05450	lysC	CodY	L-box	0	0	-3.099	-0.697	0	0
KQU62_05455	asd	CodY	0	0	0	-3.331	-0.744	-1.29	0.431
KQU62_05460	dapA	CodY	L-box	0	0	-3.294	-0.718	-0.674	0.371
KQU62_05465	dapB	CodY	L-box	0	0	-3.298	-0.876	-2.39	0
KQU62_05470	dapD	CodY	L-box	0	0	-3.122	-0.636	-1.02	0
KQU62_05475	dapI	CodY	L-box	0	0	-2.842	-0.382	-1.08	0
KQU62_05480	alr2	CodY	L-box	0	0	-2.847	-0.387	0	0
KQU62_05485	lysA	CodY	L-box	0	0	-1.773	-0.264	-0.392	0
KQU62_05490	msa	0	0	0	0	-1.15	0.896	0	0
KQU62_05495	cspA	0	0	0	0	-1.135	-0.375	-0.505	-0.46
KQU62_05510	xpaC	0	0	0	0	1.17	0	0	0
KQU62_05520	brnQ3	0	0	0	0	1.51	0.718	0	0
KQU62_05530	-	0	0	0	0	1.029	0.256	0.261	0.299
KQU62_05550	sucA	0	0	0	0	-1.399	0	0.262	0
KQU62_05555	arlS	0	0	0	0	-1.029	0	0	0
KQU62_05615	msrA1	0	0	0	0	0.236	-0.22	1.05	0
KQU62_05650	-	0	0	0	0	-1.087	0	0	0

						Transcriptome (Log2 Fold Change)		Proteome (Log2 Fold Change)	
**Locus SG511**	**Gene Name**	**Regulator 1**	**Regulator 2**	**Regulator 3**	**Regulator 4**	**CmR-02**	**02REV**	**CmR-02**	**02REV**
KQU62_05655	-	PreQ1 riboswitch	0	0	0	1.373	1.358	-0.673	0.384
KQU62_05660	rnhA	0	0	0	0	1.133	0	0	0
KQU62_05670	norB	0	0	0	0	-3.182	-0.901	0	0
KQU62_05675	-	CodY	0	0	0	-4.349	-1.552	0	-0.798
KQU62_05680	tdcB	CodY	0	0	0	-5.503	-1.866	0	-0.651
KQU62_05685	ald	Rex	0	0	0	-5.277	-1.777	0	-0.737
KQU62_05700	piuB	0	0	0	0	1.283	0.85	0	0
KQU62_05710	-	0	0	0	0	1.678	0.811	0	0
KQU62_05750	nth	0	0	0	0	0.795	0	0.979	1.28
KQU62_05770	birA	0	0	0	0	0.566	0	2.39	0
KQU62_05775	papS	0	0	0	0	0.387	0	1.94	1.82
KQU62_05785	mazG	0	0	0	0	1.16	0	0	0
KQU62_05885	-	0	0	0	0	1.12	0	0.379	0
KQU62_05890	-	0	0	0	0	2.042	1.2	0	0
KQU62_05955	srrB	Rex	0	0	0	-1.609	0	0	0
KQU62_05960	srrA	Rex	0	0	0	-1.269	0	0	-0.492
KQU62_05985	xerD	0	0	0	0	-1.75	-0.203	0	0
KQU62_06040	malR	0	0	0	0	-2.005	-0.61	0	0
KQU62_06045	-	0	0	0	0	1.029	0	0	0
KQU62_06170	-	0	0	0	0	1.348	0.265	0.432	0
KQU62_06175	-	0	0	0	0	1.01	0	0	0
KQU62_06205	aroK	0	0	0	0	-1.506	0	0	0
KQU62_06215	comGF	0	0	0	0	1.088	1.347	0	0
KQU62_06265	-	0	0	0	0	-0.328	0	0	1.64
KQU62_06320	trmK	0	0	0	0	0.629	0.762	-1.34	0
KQU62_06375	phoH	0	0	0	0	-1.222	-0.331	0	0

Locus SG511	Gene Name	Regulator 1	Regulator 2	Regulator 3	Regulator 4	Transcriptome (Log2 Fold Change)		Proteome (Log2 Fold Change)	
						CmR-02	02REV	CmR-02	02REV
KQU62_06390	yqeZ	0	0	0	0	-1.025	0	0	0
KQU62_06410	prmA	0	0	0	0	2.627	0	1.56	0
KQU62_06415	dnaJ	CtsR	HrcA	0	0	2.881	0.19	1.33	0.338
KQU62_06420	dnaK	CtsR	HrcA	0	0	2.559	0	0.725	0
KQU62_06425	grpE	CtsR	HrcA	0	0	2.591	0	1.07	0
KQU62_06430	hrcA	CtsR	HrcA	0	0	2.128	0	1.58	-1.2
KQU62_06515	-	0	0	0	0	1.067	0.454	0	0
KQU62_06520	-	0	0	0	0	1.631	0	0	0
KQU62_06525	-	0	0	0	0	0.549	0.344	0	1.4
KQU62_06530	-	CcpA	0	0	0	-1.44	0	0	0
KQU62_06535	lamB	CcpA	0	0	0	-1.116	0.612	0	0
KQU62_06540	accC	CcpA	0	0	0	-1.23	0.355	0	0
KQU62_06545	accB_1	CcpA	0	0	0	-1.169	0.588	0	0
KQU62_06550	ahs2	CcpA	0	0	0	-1.162	0.753	0	0
KQU62_06555	dur1	CcpA	0	0	0	-1.107	0.735	0	0
KQU62_06575	-	0	0	0	0	0.616	0.607	0	1.12
KQU62_06595	-	0	0	0	0	0	0	0	-2.11
KQU62_06605	recD2	0	0	0	0	0.316	0.321	1.21	1.42
KQU62_06610	-	0	0	0	0	1.132	0.722	0	0
KQU62_06655	-	0	0	0	0	0.945	1.163	0	0
KQU62_06770	-	0	0	0	0	-1.262	0.577	0	0
KQU62_06790	comC	0	0	0	0	0	1.533	0	0
KQU62_06815	-	0	0	0	0	-1.368	0	0	0
KQU62_06820	ISL-3	0	0	0	0	-1.174	0	0	0
KQU62_06840	hemC	0	0	0	0	-1.162	0	0	-0.372
KQU62_06845	hemX	0	0	0	0	-1.572	0	0	0
KQU62_06850	hemA	0	0	0	0	-1.887	0	0	0

Locus SG511	Gene Name	Regulator 1	Regulator 2	Regulator 3	Regulator 4	Transcriptome (Log2 Fold Change) CmR-02	Transcriptome (Log2 Fold Change) 02REV	Proteome (Log2 Fold Change) CmR-02	Proteome (Log2 Fold Change) 02REV
KQU62_06855	yihA	0	0	0	0	1.342	0.621	0.236	0
KQU62_06860	clpX	0	0	0	0	1.536	0.26	0.766	0
KQU62_06865	tig	0	0	0	0	1.095	0.568	0	0
KQU62_06875	-	0	0	0	0	0.566	0	0	-1.57
KQU62_06880	-	0	0	0	0	1.001	0.308	0	0
KQU62_06925	nrdR	0	0	0	0	-1.035	-0.28	0	0
KQU62_06930	gapB	0	0	0	0	-1.188	-1.321	0.389	0.569
KQU62_06965	icd	0	0	0	0	1.105	0.373	0.57	0.269
KQU62_06970	citZ	0	0	0	0	0.72	0	1.74	0
KQU62_07015	-	0	0	0	0	-0.291	0	-1.48	0
KQU62_07040	ald2	CcpA	0	0	0	0	0	1.04	0
KQU62_07045	uspA1	0	0	0	0	-2.165	-1.287	0	-0.539
KQU62_07055	-	0	0	0	0	1.226	0	0.607	-0.283
KQU62_07060	tpx	0	0	0	0	1.264	-0.351	0.601	0
KQU62_07100	-	0	0	0	0	1.104	0	0	-1.33
KQU62_07110	-	CodY	0	0	0	-1.736	0	-1.88	0
KQU62_07115	serA	CodY	0	0	0	-1.751	-0.377	-1.35	0.483
KQU62_07125	-	0	0	0	0	-1.303	0	-1.11	0
KQU62_07130	nagE	0	0	0	0	-1.07	-0.22	-0.957	0
KQU62_07140	htrA1	0	0	0	0	1.001	0	0.57	0
KQU62_07155	isdH	Fur	0	0	0	1.551	0	0	0
KQU62_07170	acsA	CodY	CcpA	0	0	-2.359	-1.157	1.82	0
KQU62_07175	acuA	CcpA	CodY	0	0	-3.59	0.233	0	0
KQU62_07180	acuC	CcpA	CodY	0	0	-3.267	-0.293	0	0
KQU62_07215	murC	0	0	0	0	1.06	0	0.491	-0.33
KQU62_07260	ccrZ	0	0	0	0	-0.477	0	1.74	0.734
KQU62_07315	-	0	0	0	0	1.069	0	0	0

Locus SG511	Gene Name	Regulator 1	Regulator 2	Regulator 3	Regulator 4	Transcriptome (Log2 Fold Change)		Proteome (Log2 Fold Change)	
						CmR-02	02REV	CmR-02	02REV
KQU62_07325	rot	RNAIII	0	0	0	-1.054	-0.301	0	0
KQU62_07330	-	0	0	0	0	1.147	0.351	0.231	0
KQU62_07335	-	0	0	0	0	2.868	0	0	0
KQU62_07340	putA	CcpA	0	0	0	-4.369	-2.23	1.49	0
KQU62_07345	ribH	FMN-box	0	0	0	1.333	0	1.5	0
KQU62_07350	ribA	FMN-box	0	0	0	1.356	0	0	0
KQU62_07355	ribB	FMN-box	0	0	0	1.535	0	0	0
KQU62_07360	ribD	FMN-box	0	0	0	1.675	0.386	0	0
KQU62_07390	sigS	0	0	0	0	1.15	0	0	0
KQU62_07395	comK2	0	0	0	0	1.276	1.524	0	0
KQU62_07400	-	0	0	0	0	1.811	0.593	0	0
KQU62_07410	-	0	0	0	0	-1.182	0	0	0
KQU62_07420	-	0	0	0	0	1.444	0.393	0	0
KQU62_07425	crcB1	0	0	0	0	1.349	1.143	0	0
KQU62_07450	pckA	CcpA	0	0	0	-1.359	-1.283	0.457	-0.578
KQU62_07475	menE	0	0	0	0	0	0	-1.33	0
KQU62_07485	-	0	0	0	0	-3.156	0.375	0	0
KQU62_07490	-	0	0	0	0	-3.17	0.457	-0.956	0
KQU62_07495	-	0	0	0	0	-1.677	1.377	0	0
KQU62_07500	-	0	0	0	0	-1.67	1.192	0	0
KQU62_07505	-	0	0	0	0	-1.369	1.139	0	0
KQU62_07510	-	0	0	0	0	-1.165	0	0	0
KQU62_07515	-	0	0	0	0	-1.585	0	0	0
KQU62_07525	pepA1	0	0	0	0	0	-1.178	0	0
KQU62_07530	-	0	0	0	0	1.391	0	0	0
KQU62_07550	hysA	0	0	0	0	-1.323	0	0	0
KQU62_07565	-	0	0	0	0	-1.888	0	0	0

Locus SG511	Gene Name	Regulator 1	Regulator 2	Regulator 3	Regulator 4	Transcriptome (Log2 Fold Change)		Proteome (Log2 Fold Change)	
						CmR-02	02REV	CmR-02	02REV
KQU62_07570	-	0	0	0	0	-2.141	-0.464	0	0
KQU62_07595	splF	0	0	0	0	1.046	0	0	0
KQU62_07625	tru.splB	0	0	0	0	-1.149	0	0	0
KQU62_07630	-	0	0	0	0	0	1.097	0	0
KQU62_07640	IS30	0	0	0	0	1.02	0.688	0	0
KQU62_07665	seu	0	0	0	0	0	1.055	0	0
KQU62_07670	sei	0	0	0	0	0	1.331	0	0
KQU62_07675	sem	0	0	0	0	0	1.422	0	0
KQU62_07680	seo	0	0	0	0	1.469	2.265	0	0
KQU62_07700	tRNA-Gly	0	0	0	0	1.309	0	0	0
KQU62_07715	tRNA-Asp	0	0	0	0	1.672	1.078	0	0
KQU62_07720	tRNA-Met	0	0	0	0	1.804	0	0	0
KQU62_07725	-	0	0	0	0	0.337	0	0	1.06
KQU62_07755	-	0	0	0	0	1.014	0	0.468	0.209
KQU62_07760	ecsA	0	0	0	0	1.626	0.343	0.422	0
KQU62_07775	-	0	0	0	0	1.053	0.462	0	0
KQU62_07780	prsA	0	0	0	0	1.298	0	0.65	0
KQU62_07795	-	0	0	0	0	-1.076	0	-0.482	0
KQU62_07805	-	0	0	0	0	1.023	0.21	0	0
KQU62_07810	-	0	0	0	0	1.536	1.189	0.256	0
KQU62_07815	airR	0	0	0	0	0	0	-1.01	0
KQU62_07850	cspR	0	0	0	0	-1.409	0	0	0
KQU62_07855	queG	0	0	0	0	-1.553	-0.321	0	0
KQU62_07860	artQ	ArgR	CodY	0	0	-4.245	-1.604	0	0
KQU62_07865	artM	ArgR	CodY	0	0	-4.385	-1.357	0	0
KQU62_07880	tRNA-Gly	0	0	0	0	0	1.26	0	0
KQU62_07925	tRNA-Met	0	0	0	0	3.226	0	0	0

Locus SG511	Gene Name	Regulator 1	Regulator 2	Regulator 3	Regulator 4	Transcriptome (Log2 Fold Change)		Proteome (Log2 Fold Change)	
						CmR-02	02REV	CmR-02	02REV
KQU62_07930	tRNA-Ser	0	0	0	0	0.49	1.018	0	0
KQU62_07935	tRNA-Asp	0	0	0	0	0.67	1.213	0	0
KQU62_08050	msbA	0	0	0	0	1.609	0.411	0	0
KQU62_08085	recX	0	0	0	0	0.575	0	0	-1.01
KQU62_08090	sgtB	0	0	0	0	-1.322	0	0	0
KQU62_08095	-	SigB	0	0	0	0.757	0	1.63	0
KQU62_08120	yfkK	0	0	0	0	1.354	0	0	0
KQU62_08125	ptpA	SigB	0	0	0	-0.459	-0.331	0	-1.42
KQU62_08170	-	0	0	0	0	1.329	0	0	0
KQU62_08190	ftnA	Fur	PerR	0	0	-4.263	-0.724	-0.54	0
KQU62_08200	-	0	0	0	0	1.435	0.592	0	-1.2
KQU62_08225	-	0	0	0	0	1.014	1.067	0	0
KQU62_08240	gatC	0	0	0	0	2.106	0.855	0	0
KQU62_08245	putP	CcpA	0	0	0	-2.983	0.154	0	2.25
KQU62_08265	pcrB	0	0	0	0	1.082	0.333	0	0
KQU62_08275	purB	0	0	0	0	1.081	0	0	0
KQU62_08280	sspB2	0	0	0	0	-1.079	0	0	0
KQU62_08285	scpB	0	0	0	0	-1.237	0	0	0
KQU62_08310	nos	0	0	0	0	-1.884	-0.484	0	-0.394
KQU62_08315	pheA	0	0	0	0	-1.241	-0.327	0	0
KQU62_08325	sdcS	CcpA	0	0	0	-1.52	-0.52	0	0
KQU62_08355	-	0	0	0	0	1.189	0	0.976	0
KQU62_08455	phage-related	SaeR	0	0	0	1.183	0.512	0	0
KQU62_08475	phage-related	0	0	0	0	3.39	0	0	0

Locus SG511	Gene Name	Regulator 1	Regulator 2	Regulator 3	Regulator 4	Transcriptome (Log2 Fold Change)		Proteome (Log2 Fold Change)	
						CmR-02	02REV	CmR-02	02REV
KQU62_08480	phage-related	0	0	0	0	3.581	0	0	0
KQU62_08495	phage-related	0	0	0	0	2.471	0	0	0
KQU62_08500	phage-related	0	0	0	0	2.869	0	0	0
KQU62_08505	phage-related	0	0	0	0	3.936	0	0	0
KQU62_08510	phage-related	0	0	0	0	2.45	0	0	0
KQU62_08515	phage-related	0	0	0	0	3.389	0	0	0
KQU62_08520	phage-related	0	0	0	0	2.707	0	0	0
KQU62_08525	phage-related	0	0	0	0	3.057	0	0	0
KQU62_08530	phage-related	0	0	0	0	2.626	0	0	0
KQU62_08535	phage-related	0	0	0	0	2.923	0	0	0
KQU62_08540	phage-related	0	0	0	0	2.631	0	0	0
KQU62_08545	phage-related	0	0	0	0	2.62	0	0	0
KQU62_08550	phage-related	0	0	0	0	2.74	0	0	0
KQU62_08555	phage-related	0	0	0	0	2.92	0	0	0

						Transcriptome (Log2 Fold Change)		Proteome (Log2 Fold Change)	
Locus SG511	Gene Name	Regulator 1	Regulator 2	Regulator 3	Regulator 4	CmR-02	02REV	CmR-02	02REV
KQU62_08560	phage-related	0	0	0	0	2.685	0	0	0
KQU62_08565	phage-related	0	0	0	0	2.847	0	0	0
KQU62_08570	phage-related	0	0	0	0	2.868	0	0	0
KQU62_08575	phage-related	0	0	0	0	2.7	0	0	0
KQU62_08580	phage-related	0	0	0	0	2.68	0	0	0
KQU62_08585	phage-related	0	0	0	0	2.596	0	0	0
KQU62_08590	phage-related	0	0	0	0	2.701	0	0	0
KQU62_08595	phage-related	0	0	0	0	2.353	0	0	0
KQU62_08600	phage-related	0	0	0	0	2.5	0	0	0
KQU62_08610	phage-related	0	0	0	0	2.358	0	0	0
KQU62_08615	phage-related	0	0	0	0	1.422	0	0	0
KQU62_08620	phage-related	0	0	0	0	1.776	0	0	0
KQU62_08625	phage-related	0	0	0	0	2.234	0	0	0
KQU62_08630	phage-related	0	0	0	0	2.219	0	0	0

Locus SG511	Gene Name	Regulator 1	Regulator 2	Regulator 3	Regulator 4	Transcriptome (Log2 Fold Change)		Proteome (Log2 Fold Change)	
						CmR-02	02REV	CmR-02	02REV
KQU62_08635	phage-related	0	0	0	0	2.228	0.411	0	0
KQU62_08640	phage-related	0	0	0	0	2.131	0	0	0
KQU62_08645	phage-related	0	0	0	0	2.286	0	0	0
KQU62_08650	phage-related	0	0	0	0	2.135	0	0	0
KQU62_08655	phage-related	0	0	0	0	2.088	0	0	0
KQU62_08660	phage-related	0	0	0	0	2.359	0	0	0
KQU62_08665	phage-related	0	0	0	0	2.146	0	0	0
KQU62_08670	phage-related	0	0	0	0	2.033	0	0	0
KQU62_08675	phage-related	0	0	0	0	2.463	0	0	0
KQU62_08680	phage-related	0	0	0	0	2.067	0	0	0
KQU62_08690	phage-related	0	0	0	0	2.261	0	0	0
KQU62_08695	phage-related	0	0	0	0	2.371	0	0	0
KQU62_08700	phage-related	0	0	0	0	2.632	0	1.22	0
KQU62_08705	phage-related	0	0	0	0	-0.989	0	-1.14	0

Locus SG511	Gene Name	Regulator 1	Regulator 2	Regulator 3	Regulator 4	Transcriptome (Log2 Fold Change) CmR-02	Transcriptome (Log2 Fold Change) 02REV	Proteome (Log2 Fold Change) CmR-02	Proteome (Log2 Fold Change) 02REV
KQU62_08710	phage-related	0	0	0	0	3.163	0	0	0
KQU62_08715	phage-related	0	0	0	0	2.998	0	0	0
KQU62_08735	phage-related	SigH	0	0	0	1.639	0	0	0
KQU62_08740	phage-related	0	0	0	0	1.163	0	0	0
KQU62_08745	lukF	SaeR	0	0	0	-2.428	-0.315	0	0
KQU62_08750	lukS	SaeR	0	0	0	-2.279	0	0	0
KQU62_08755	-	SigB	0	0	0	-0.196	0	0	-1.82
KQU62_08770	-	0	0	0	0	-1.114	-0.464	0	0
KQU62_08775	-	0	0	0	0	-1.208	-0.758	0	0
KQU62_08780	groL	CtsR	HrcA	0	0	2.96	-0.447	1.42	0
KQU62_08785	groES	CtsR	HrcA	0	0	3.365	-0.679	1.24	0
KQU62_08815	agrB	AgrA	CodY	0	0	-1.823	0	0	0
KQU62_08860	-	0	0	0	0	0	-0.43	-1.29	-0.406
KQU62_08865	-	CymR	0	0	0	0.178	-0.238	-2.18	0
KQU62_08885	-	0	0	0	0	-0.836	0	-1.4	0
KQU62_08915	ilvD	CodY	0	0	0	-3.319	0	0	0
KQU62_08920	ilvB	CodY	0	0	0	-3.128	0.308	0	0
KQU62_08925	ilvN	CodY	0	0	0	-2.567	0	0	0
KQU62_08930	ilvC	CodY	0	0	0	-3.114	0	0	0
KQU62_08935	leuA	CodY	0	0	0	-2.966	0	0	0
KQU62_08940	leuB	CodY	0	0	0	-2.038	0	0	0
KQU62_08945	leuC	CodY	0	0	0	-1.888	0	0	0
KQU62_08950	leuD	CodY	0	0	0	-1.784	0	0	0

						Transcriptome (Log2 Fold Change)		Proteome (Log2 Fold Change)	
**Locus SG511**	**Gene Name**	**Regulator 1**	**Regulator 2**	**Regulator 3**	**Regulator 4**	**CmR-02**	**02REV**	**CmR-02**	**02REV**
KQU62_08955	ilvA2	CodY	0	0	0	-1.506	0	0	0
KQU62_08960	-	0	0	0	0	3.164	3.318	0	0
KQU62_08985	tRNA-Leu	0	0	0	0	1.549	0	0	0
KQU62_09085	cshA	0	0	0	0	1.268	0.958	0	0
KQU62_09105	IS1182	0	0	0	0	3.249	0	0	0
KQU62_09110	rexA	SigB	0	0	0	-1.403	-0.719	0	0
KQU62_09115	-	0	0	0	0	1.332	0	0	0
KQU62_09130	-	0	0	0	0	-1.075	0	0	0
KQU62_09135	yidC	0	0	0	0	1.28	0.543	0	0
KQU62_09140	thiE	0	0	0	0	1.239	0	0	0
KQU62_09145	thiM	0	0	0	0	1.807	0	0	0
KQU62_09150	thiD2	0	0	0	0	2.138	0	0	0
KQU62_09155	tenA	0	0	0	0	2.265	0	0	0
KQU62_09160	sceD	WalR	0	0	0	7.255	-1.377	2.68	0
KQU62_09290	-	SigB	0	0	0	1.529	-0.423	0.697	-0.214
KQU62_09315	pyrG	0	0	0	0	1.136	0.349	0	0
KQU62_09320	rpoE	0	0	0	0	1.094	0	-0.377	0
KQU62_09325	-	0	0	0	0	1.485	0.406	0.188	0
KQU62_09375	dps	PerR	0	0	0	-1.079	-0.706	0	0
KQU62_09395	manA	0	0	0	0	0	0.435	-2.13	0
KQU62_09420	-	0	0	0	0	-1.312	0	0	0
KQU62_09430	-	0	0	0	0	1.19	0	0.662	0
KQU62_09440	glmS	glms leader	0	0	0	-1.383	-0.881	0.268	0
KQU62_09445	mtlF	MtlR	0	0	0	-1.928	-2.061	0	0
KQU62_09490	tRNA-Lys	0	0	0	0	1.576	1.782	0	0
KQU62_09505	tRNA-Val	0	0	0	0	1.097	1.239	0	0
KQU62_09530	16SrRNA	0	0	0	0	1.825	1.195	0	0

						Transcriptome (Log2 Fold Change)		Proteome (Log2 Fold Change)	
Locus SG511	Gene Name	Regulator 1	Regulator 2	Regulator 3	Regulator 4	CmR-02	02REV	CmR-02	02REV
KQU62_09535	-	0	0	0	0	1.956	0	0	0
KQU62_09570	-	LexA	CcpA	0	0	-1.231	-0.769	0	0
KQU62_09575	-	CcpA	0	0	0	-2.493	-1.266	0	0
KQU62_09580	-	0	0	0	0	-1.703	-0.432	0	0
KQU62_09585	htsC	Fur	0	0	0	1.863	0	0	0
KQU62_09590	htsB	Fur	0	0	0	2.624	0	0	0
KQU62_09595	htsA	Fur	0	0	0	3.121	0	0.388	0
KQU62_09605	sfaC	Fur	SigB	0	0	1.181	-0.18	0	0
KQU62_09610	sfnaB	Fur	SigB	0	0	1.134	-0.26	1.07	0
KQU62_09615	sfaA	Fur	0	0	0	1.391	0	0	0
KQU62_09650	-	0	0	0	0	1.049	-0.371	0.793	-0.223
KQU62_09655	-	0	0	0	0	1.015	0	0	0
KQU62_09660	lacG	LacR	0	0	0	-1.682	0	1.62	0
KQU62_09665	lacE	LacR	0	0	0	-2.45	-0.354	3.11	0
KQU62_09670	lacF	LacR	0	0	0	-3.01	-0.829	0	0
KQU62_09675	lacD	LacR	0	0	0	-3.063	-0.592	1.95	0
KQU62_09680	lacC	LacR	0	0	0	-3.16	-0.762	0	0
KQU62_09685	lacB	LacR	0	0	0	-3.393	-0.986	3.24	0
KQU62_09690	lacA	LacR	0	0	0	-3.546	-0.987	4.68	2.39
KQU62_09750	-	0	0	0	0	0	-0.303	1.58	1.12
KQU62_09755	map-w	SigB	0	0	0	0	-1.38	0	0
KQU62_09760	budA	0	0	0	0	-2.435	-1.08	0	-0.634
KQU62_09765	alsS	0	0	0	0	-2.338	-0.948	0	-0.568
KQU62_09775	-	0	0	0	0	1.604	0	0	0
KQU62_09810	cbiO2	0	0	0	0	-0.383	0	0	1.1
KQU62_09990	glcU	0	0	0	0	1.217	-0.277	0	0
KQU62_10035	marR	0	0	0	0	1.279	1.133	0	0

						Transcriptome (Log2 Fold Change)		Proteome (Log2 Fold Change)	
Locus SG511	Gene Name	Regulator 1	Regulator 2	Regulator 3	Regulator 4	CmR-02	02REV	CmR-02	02REV
KQU62_10090	moaB	0	0	0	0	-1.045	0	0	-0.41
KQU62_10095	moeB	0	0	0	0	-1.555	-0.158	0	0
KQU62_10100	modC	0	0	0	0	-1.694	-0.304	-1.61	0
KQU62_10105	modB	0	0	0	0	-1.833	-0.226	0	0
KQU62_10110	modA	0	0	0	0	-1.412	0	-0.326	0
KQU62_10135	fhuD2	Fur	0	0	0	2.21	0.411	-0.342	0
KQU62_10150	ureA	0	0	0	0	3.222	0	0	0
KQU62_10155	ureB	0	0	0	0	3.098	0	0	0
KQU62_10160	ureC	0	0	0	0	2.722	-0.288	0	0
KQU62_10165	ureE	0	0	0	0	2.355	0	0	0
KQU62_10170	ureF	0	0	0	0	2.46	0	0	0
KQU62_10175	ureG	0	0	0	0	2.216	-0.396	1.86	0
KQU62_10180	ureD	0	0	0	0	2.231	0	0	0
KQU62_10185	sarR	0	0	0	0	-1.523	-0.312	0	0
KQU62_10195	sarY	SigB	0	0	0	-1.25	0.588	0	0
KQU62_10200	-	SigB	0	0	0	-1.558	0.448	0	0
KQU62_10205	ssaA	WalR	GraR	0	0	2.875	1.444	-0.489	0.81
KQU62_10220	-	SigB	0	0	0	1.195	0.521	-0.228	0
KQU62_10230	ssaA2	WalR	0	0	0	1.225	0	0	0
KQU62_10255	yrhD	SigB	0	0	0	-2.356	-0.463	0	0
KQU62_10260	fdhF	SigB	0	0	0	-2.249	-0.299	-1.21	0
KQU62_10275	-	CcpA	0	0	0	-1.046	-0.396	0	0
KQU62_10280	spdB	0	0	0	0	2.274	0.924	0	0
KQU62_10290	rpiRc	0	0	0	0	-1.943	0	0	0
KQU62_10325	-	0	0	0	0	-0.538	0.36	3.08	3.1
KQU62_10330	glvC	GlvR	CcpA	0	0	-1.538	0	0	0
KQU62_10335	glvR	CcpA	0	0	0	0.827	1.119	-1.37	0

Locus SG511	Gene Name	Regulator 1	Regulator 2	Regulator 3	Regulator 4	Transcriptome (Log2 Fold Change) CmR-02	Transcriptome (Log2 Fold Change) 02REV	Proteome (Log2 Fold Change) CmR-02	Proteome (Log2 Fold Change) 02REV
KQU62_10355	-	SigB	0	0	0	-1.518	-0.506	0	-1.49
KQU62_10360	hipO2	0	0	0	0	-2.026	-0.389	0	0
KQU62_10390	sdpC	0	0	0	0	0	1.161	-1.1	0.905
KQU62_10405	galM	0	0	0	0	-1.265	-0.56	-0.561	0
KQU62_10410	ynfA	0	0	0	0	-1.518	-0.778	0	0
KQU62_10415	-	0	0	0	0	-1.142	0	0	0
KQU62_10430	-	0	0	0	0	-2.149	-0.164	-1.11	0
KQU62_10440	gltS	CcpA	0	0	0	1.72	1.32	0	0
KQU62_10455	-	0	0	0	0	2.109	0	0	0
KQU62_10460	-	0	0	0	0	1.617	0	0	0
KQU62_10485	-	0	0	0	0	1.831	1.002	0	0
KQU62_10490	IS1182	0	0	0	0	2.434	0	0	0
KQU62_10515	-	0	0	0	0	1.122	0	0	0
KQU62_10550	-	0	0	0	0	1.57	0	0	0
KQU62_10555	-	0	0	0	0	-0.167	-1.566	0	0
KQU62_10560	-	0	0	0	0	-0.158	-1.758	0	0
KQU62_10570	lctP_2	Rex	0	0	0	-3.764	-0.59	0	0
KQU62_10585	paiA	0	0	0	0	1.54	0	1.95	0
KQU62_10590	yhfP	0	0	0	0	1.438	-0.283	1.18	0
KQU62_10595	-	0	0	0	0	1.316	0	0	0
KQU62_10600	-	0	0	0	0	1.138	-0.573	0	0
KQU62_10605	iruO	0	0	0	0	2.646	0	2.46	0
KQU62_10610	-	0	0	0	0	-2.447	0	0	0
KQU62_10635	scrA	0	0	0	0	-1.91	0	0	0
KQU62_10650	-	SigB	0	0	0	-1.144	-0.708	0	-0.618
KQU62_10670	sarZ	0	0	0	0	-1.4	0	1.68	2.24
KQU62_10675	hsp20	0	0	0	0	1.657	0	0	0

						Transcriptome (Log2 Fold Change)		Proteome (Log2 Fold Change)	
**Locus SG511**	**Gene Name**	**Regulator 1**	**Regulator 2**	**Regulator 3**	**Regulator 4**	**CmR-02**	**02REV**	**CmR-02**	**02REV**
KQU62_10680	narK	Rex	NreC	0	0	-8.211	-1.746	0	0
KQU62_10695	nreC	Rex	NreC	0	0	-1.803	-0.472	0	0
KQU62_10700	nreB	Rex	NreC	0	0	-2.487	-0.62	0	0
KQU62_10705	nreA	Rex	NreC	0	0	-3.073	-0.857	0	0
KQU62_10710	narI	Rex	NreC	0	0	-4.585	-1.121	0	0
KQU62_10715	narJ	Rex	NreC	0	0	-7.6	-1.499	0	0
KQU62_10720	narH	Rex	NreC	0	0	-8.341	-1.762	0	-1.13
KQU62_10725	narG	Rex	NreC	0	0	-7.919	-1.683	0	-0.775
KQU62_10730	nasF	Rex	NreC	0	0	-7.306	-2.333	0	0
KQU62_10735	nasE	Rex	NreC	0	0	-8.007	-2.451	0	0
KQU62_10740	nasD	Rex	NreC	0	0	-7.491	-2.044	0	0
KQU62_10745	nirR	Rex	NreC	0	0	-6.749	-1.291	0	0
KQU62_10755	-	0	0	0	0	-1.17	0	-0.585	0
KQU62_10775	dsbA	0	0	0	0	-1.377	-0.254	0	0
KQU62_10780	priA	0	0	0	0	-1.6	0	-1.02	0
KQU62_10810	-	0	0	0	0	-1.664	-0.621	0	0
KQU62_10815	gpmA	0	0	0	0	-4.306	-1.507	-1.15	-0.467
KQU62_10835	hlgA	0	0	0	0	-1.628	-0.819	0	0
KQU62_10850	bioX	0	0	0	0	1.111	0.774	0	0
KQU62_10875	bioD	0	0	0	0	0	1.049	0	0
KQU62_10890	msbA3	0	0	0	0	1.011	0.721	0	0
KQU62_10895	-	0	0	0	0	-0.844	-1.006	0	0
KQU62_10915	bcr	0	0	0	0	-1.018	0	0	0
KQU62_10920	-	0	0	0	0	1.21	0	0	0
KQU62_10930	-	0	0	0	0	1.512	0	0	0
KQU62_10935	cycA	CcpA	CodY	0	0	-1.677	-1.368	0	0
KQU62_10945	kimA	0	0	0	0	-1.683	-0.198	0	0

Locus SG511	Gene Name	Regulator 1	Regulator 2	Regulator 3	Regulator 4	Transcriptome (Log2 Fold Change)		Proteome (Log2 Fold Change)	
						CmR-02	02REV	CmR-02	02REV
KQU62_10955	-	0	0	0	0	1.155	-0.591	0.866	-0.319
KQU62_10970	opuCD	CodY	0	0	0	-1.717	-0.511	0	0
KQU62_10975	opuCC	CodY	0	0	0	-2.06	-0.84	0.257	-0.184
KQU62_10980	opuCB	CodY	0	0	0	-2.311	-0.961	0	0
KQU62_10985	opuCA	CodY	0	0	0	-2.602	-1.131	0.262	0
KQU62_11010	pnbA	SigB	0	0	0	-1.569	-0.759	0	0
KQU62_11070	cntD	0	0	0	0	1.336	0.587	0	0
KQU62_11090	cntM	0	0	0	0	2.317	0	0	0
KQU62_11095	cntL	0	0	0	0	2.136	0	0	0
KQU62_11100	cntK	0	0	0	0	2.506	0	0	0
KQU62_11115	-	0	0	0	0	-2.181	-0.263	0	0
KQU62_11120	-	SigB	0	0	0	-0.768	-1.113	0	-0.598
KQU62_11125	-	0	0	0	0	1.686	0	0	0
KQU62_11130	-	0	0	0	0	1.265	0	0.81	0
KQU62_11140	-	0	0	0	0	2.576	0	0	0
KQU62_11145	-	Rex	0	0	0	-4.473	-1.532	0	0
KQU62_11210	gntP	CcpA	GntR	0	0	-1.218	0	1.22	0
KQU62_11215	gntK	CcpA	GntR	0	0	-2.037	-0.993	2.38	0
KQU62_11220	gntR	CcpA	GntR	0	0	-3.328	-1.993	0	-1.74
KQU62_11225	-	0	0	0	0	1.231	-0.271	0	0
KQU62_11230	relP	0	0	0	0	1.571	0	2.41	0
KQU62_11255	-	0	0	0	0	-2.741	0.793	0	0
KQU62_11260	-	0	0	0	0	-3.271	1.074	0	0
KQU62_11270	-	0	0	0	0	-1.221	0	-0.938	0
KQU62_11275	-	0	0	0	0	-1.753	0	0	0
KQU62_11315	frp	Rex	0	0	0	1.682	-0.434	0.695	0
KQU62_11320	ddh	Rex	0	0	0	-1.56	-0.468	0	-0.467

Locus SG511	Gene Name	Regulator 1	Regulator 2	Regulator 3	Regulator 4	Transcriptome (Log2 Fold Change)		Proteome (Log2 Fold Change)	
						CmR-02	02REV	CmR-02	02REV
KQU62_11340	pat	0	0	0	0	2.157	0	0	0
KQU62_11360	sdaAB	CcpA	0	0	0	1.468	0.522	0	0
KQU62_11365	pfoR	CcpA	0	0	0	1.012	0.371	0	0
KQU62_11390	-	0	0	0	0	0.434	0	1.42	0.442
KQU62_11395	glcB	0	0	0	0	-1.163	0.591	-0.807	0.278
KQU62_11415	cidR	0	0	0	0	0.925	0.553	0	1.05
KQU62_11420	-	0	0	0	0	-1.228	-0.436	0	0
KQU62_11425	amiD2	WalR	0	0	0	0.61	1.34	0	0
KQU62_11435	mvaS	CcpA	0	0	0	1.557	-0.261	0.768	0
KQU62_11440	-	0	0	0	0	0	-0.448	1.48	1.06
KQU62_11455	feoB	Fur	0	0	0	1.126	0.278	0	0
KQU62_11460	feoA	Fur	0	0	0	1.303	0	0	0
KQU62_11480	rocA	CcpA	0	0	0	0.554	-0.755	1.07	0
KQU62_11485	-	0	0	0	0	1.091	-0.428	0	0
KQU62_11490	cwrA	0	0	0	0	-0.459	1.396	0	0
KQU62_11495	copA	0	0	0	0	-1.029	-0.164	0	0
KQU62_11505	-	CodY	0	0	0	-1.185	-0.65	-0.446	0.332
KQU62_11510	ywfG	CodY	0	0	0	-1.218	-0.738	0	0
KQU62_11515	crtN	SigB	0	0	0	0.72	-0.418	0	-1.29
KQU62_11530	crtI	SigB	0	0	0	1.121	0	0	0
KQU62_11535	crtO	SigB	0	0	0	1.652	0	0	0
KQU62_11540	ssaA1	WalR	RNAIII	0	0	1.332	0	0.74	1.85
KQU62_11545	oatA	0	0	0	0	1.218	0.621	0	0
KQU62_11550	isaA	WalR	0	0	0	2.38	0.978	0.396	-0.625
KQU62_11555	pfoS/R	CodY	0	0	0	-1.342	0	0	0
KQU62_11585	nrmA	0	0	0	0	1.232	0.294	0	0
KQU62_11595	gbaA	0	0	0	0	1.214	0	0	0

Locus SG511	Gene Name	Regulator 1	Regulator 2	Regulator 3	Regulator 4	Transcriptome (Log2 Fold Change)		Proteome (Log2 Fold Change)	
						CmR-02	02REV	CmR-02	02REV
KQU62_11600	gbaB	0	0	0	0	1.155	0	0	0
KQU62_11605	hisI	SigB	0	0	0	1.136	0	0	0
KQU62_11655	-	0	0	0	0	0	0	0	-1.09
KQU62_11710	-	CodY	0	0	0	-2.503	-0.393	0	0
KQU62_11715	gabT	CodY	0	0	0	-1.707	-0.255	0	0
KQU62_11725	fdaB	0	0	0	0	1.468	-0.939	0.999	-0.319
KQU62_11745	-	SigB	0	0	0	-0.665	-1.085	0	0
KQU62_11755	betA	0	0	0	0	1.153	-2.75	-0.925	-3.83
KQU62_11760	betB	0	0	0	0	1.299	-2.872	0	-4.5
KQU62_11775	cudT	0	0	0	0	0.867	-1.467	0	0
KQU62_11790	nrdG	NrdR	0	0	0	-0.992	-1.768	0	0
KQU62_11795	nrdD	NrdR	0	0	0	-0.586	-1.129	0	0
KQU62_11800	citM	0	0	0	0	1.323	0	0	0
KQU62_11860	-	0	0	0	0	1.167	0	0	0
KQU62_11895	arcC	ArgR	CcpA	Rex	ArcR	-1.979	-1.699	0	-0.527
KQU62_11900	arcD	ArgR	CcpA	Rex	ArcR	-3.233	-1.99	0	0
KQU62_11905	arcB	ArgR	CcpA	Rex	ArcR	-2.756	-2.175	0	-0.556
KQU62_11910	arcA	ArgR	CcpA	Rex	ArcR	-2.159	-2.216	0	-0.686
KQU62_11920	argR	0	0	0	0	-2.153	0	0	0
KQU62_11925	aur	CodY	0	0	0	-1.521	0	0	0
KQU62_11940	manR	CcpA	0	0	0	-1.997	0.281	0	0
KQU62_11945	manP	ManR	CcpA	0	0	-2.643	-1.094	0	0
KQU62_11980	gtfA	SigB	0	0	0	-0.835	-0.183	-1.36	-0.954
KQU62_11985	secA2	SigB	0	0	0	-1.137	-0.182	3.05	0
KQU62_11990	asp3	0	0	0	0	-1.132	0	0	0
KQU62_11995	asp2	0	0	0	0	-1.234	0	0	0
KQU62_12000	asp1	0	0	0	0	-1.103	0	-1.76	0

Locus SG511	Gene Name	Regulator 1	Regulator 2	Regulator 3	Regulator 4	Transcriptome (Log2 Fold Change)		Proteome (Log2 Fold Change)	
						CmR-02	02REV	CmR-02	02REV
KQU62_12010	sasA	0	0	0	0	-1.482	0	0	0
KQU62_12020	-	0	0	0	0	-2.285	-0.279	0	0
KQU62_12025	-	0	0	0	0	-2.023	0	0	0
KQU62_12030	-	SigB	0	0	0	-1.204	-0.579	0	0
KQU62_12040	msrA3	0	0	0	0	1.228	0.838	0	0
KQU62_12060	cap1A	0	0	0	0	1.303	0	0	0
KQU62_12090	lip	CcpA	0	0	0	-3.231	-1.777	0	0
KQU62_12135	hisZ	HisR	CodY	0	0	0	1.115	0	0
KQU62_12140	-	0	0	0	0	-1.168	0	0	0
KQU62_12155	mtsB	0	0	0	0	-1.522	0.428	0	0
KQU62_12160	mtsA	0	0	0	0	-1.861	0.445	0	0
KQU62_12165	-	0	0	0	0	-2.242	0.348	0	0
KQU62_12180	-	0	0	0	0	2.843	0.362	2.38	0
KQU62_12195	cna	0	0	0	0	-1.89	-0.372	0	0
KQU62_12200	-	0	0	0	0	-1.323	-0.729	0	0
KQU62_12220	-	0	0	0	0	1.729	-0.365	0.66	0
KQU62_12250	-	0	0	0	0	-0.543	0.577	0	1.56
KQU62_12265	cspB	0	0	0	0	1.205	0.883	-2.23	-3.29
KQU62_12270	-	0	0	0	0	-1.068	-0.392	0	0
KQU62_12275	-	0	0	0	0	-2.368	-0.692	0	0
KQU62_12280	-	0	0	0	0	-2.408	-0.963	0	0
KQU62_12305	rnpA	0	0	0	0	1.901	0.921	0	0
KQU62_12345	nnrD	0	0	0	0	-0.388	0	1.27	0
KQU62_12350	hutH	CcpA	0	0	0	-1.251	-0.631	0	0
KQU62_12360	azlC	0	0	0	0	-2.306	0	0	0
KQU62_12365	azlD	0	0	0	0	-2.503	0	0	0
KQU62_12375	-	0	0	0	0	0.731	0.339	0	1.31

Locus SG511	Gene Name	Regulator 1	Regulator 2	Regulator 3	Regulator 4	Transcriptome (Log2 Fold Change)		Proteome (Log2 Fold Change)	
						CmR-02	02REV	CmR-02	02REV
KQU62_12395	purA	PurR	0	0	0	2.483	0	0.426	-0.206
KQU62_12430	walJ	0	0	0	0	0.773	0	1.95	0
KQU62_12435	adsA	0	0	0	0	-2.614	0	0	0
KQU62_12445	sirC	Fur	0	0	0	1.164	-0.331	0	0
KQU62_12450	sirB	Fur	0	0	0	2.111	0	0	0
KQU62_12455	sirA	Fur	0	0	0	3.328	0	1.08	0
KQU62_12460	sbnA	Fur	0	0	0	5.989	0	0	-1.22
KQU62_12465	sbnB	Fur	0	0	0	4.659	-1.348	3.17	0
KQU62_12470	sbnC	Fur	0	0	0	4.342	-1.033	0	0
KQU62_12475	sbnD	Fur	0	0	0	3.91	-0.646	0	0
KQU62_12480	sbnE	Fur	0	0	0	3.399	-0.797	0	0
KQU62_12485	sbnF	Fur	0	0	0	2.565	-0.438	0	0
KQU62_12490	sbnG	Fur	0	0	0	2.335	-0.442	0	0
KQU62_12495	sbnH	Fur	0	0	0	2.041	-0.591	0	0
KQU62_12500	sbnI	Fur	0	0	0	2.196	0	0	0
KQU62_12510	butA	CodY	0	0	0	-1.66	-0.951	0	0
KQU62_12530	cap8H	0	0	0	0	2.406	1.954	0	0
KQU62_12535	cps2H	0	0	0	0	1.299	0.861	0	0
KQU62_12550	sasD	0	0	0	0	-3.502	-3.062	0	0
KQU62_12560	deoD1	0	0	0	0	-1.521	-1.594	0	0
KQU62_12605	ushA	0	0	0	0	-1.076	-0.309	0	0
KQU62_12620	adhE	Rex	0	0	0	-4.587	-1.905	0	-0.492
KQU62_12625	cap5A	CodY	SigB	0	0	-2.56	-0.276	0	0
KQU62_12630	cap5B	CodY	SigB	0	0	-2.394	-0.286	0	0
KQU62_12635	cap5C	CodY	SigB	0	0	-2.687	-0.277	0	0
KQU62_12640	cap5D	CodY	SigB	0	0	-2.907	-0.395	0	0
KQU62_12645	cap5E	CodY	SigB	0	0	-2.654	-0.333	0	0

Locus SG511	Gene Name	Regulator 1	Regulator 2	Regulator 3	Regulator 4	Transcriptome (Log2 Fold Change)		Proteome (Log2 Fold Change)	
						CmR-02	02REV	CmR-02	02REV
KQU62_12650	cap5F	CodY	SigB	0	0	-2.433	-0.292	0	0
KQU62_12655	cap5G	CodY	SigB	0	0	-2.353	-0.303	0	0
KQU62_12660	cap8I	CodY	SigB	0	0	-1.884	0	0	0
KQU62_12665	cap8J	CodY	SigB	0	0	-1.672	0	0	0
KQU62_12670	capJ	CodY	SigB	0	0	-1.508	0	0	0
KQU62_12675	cap8K	CodY	SigB	0	0	-1.208	0	0	0
KQU62_12680	cap5L	CodY	SigB	0	0	-1.108	0	0	0
KQU62_12715	aldA	CcpA	0	0	0	-1.343	-2.45	1.46	-0.321
KQU62_12725	-	SigB	0	0	0	0	-1.226	0	0
KQU62_12780	-	GraR	0	0	0	-2.481	-0.837	0	0
KQU62_12795	argC	ArgR	0	0	0	-1.047	-0.364	0	0
KQU62_12805	brnQ	CodY	0	0	0	-2.017	0.233	-1.35	0.687
KQU62_12825	ptsG	CcpA	0	0	0	-1.255	0.385	-0.633	0
KQU62_12830	mupG	MurR	CcpA	0	0	-2.738	-0.913	0	0
KQU62_12835	murQ	MurR	0	0	0	-1.147	0	0	0
KQU62_12855	hsdR	0	0	0	0	-1.103	0	-0.611	0.354
KQU62_12860	-	0	0	0	0	-1.058	0	0	0
KQU62_12865	oppF	CymR	0	0	0	-2.045	0	-1.67	0
KQU62_12870	oppB	0	0	0	0	-3.36	0.504	0	0
KQU62_12875	-	0	0	0	0	-2.932	0.45	0	0
KQU62_12880	rlp	0	0	0	0	-2.707	0.476	0	0
KQU62_12885	ggt	0	0	0	0	-1.802	0.355	0	0
KQU62_12895	-	0	0	0	0	1.389	0	0	0
KQU62_12900	malK	MalR	CcpA	0	0	-4.701	-2.618	0	0
KQU62_12905	malE	MalR	CcpA	0	0	-3.469	-1.934	0	0
KQU62_12910	malF	MalR	CcpA	0	0	-1.604	0	0	0
KQU62_12915	malD	MalR	CcpA	0	0	-1.006	0.479	0	0

Locus SG511	Gene Name	Regulator 1	Regulator 2	Regulator 3	Regulator 4	Transcriptome (Log2 Fold Change) CmR-02	Transcriptome (Log2 Fold Change) 02REV	Proteome (Log2 Fold Change) CmR-02	Proteome (Log2 Fold Change) 02REV
KQU62_12935	-	SaeR	0	0	0	0	1.148	0	0
KQU62_12940	uhpT	CcpA	0	0	0	-2.798	0	0	0
KQU62_12955	hptA	0	0	0	0	-1.219	-0.198	0	0
KQU62_12960	pflB	CcpA	Rex	0	0	-6.897	-1.228	0	-0.61
KQU62_12965	pflA	Rex	CcpA	0	0	-7.072	-1.019	0	0
KQU62_12970	-	0	0	0	0	-5.089	0	0	0
KQU62_12980	SCIN_2	0	0	0	0	1.549	0.478	0	0
KQU62_12995	fadA	CcpA	0	0	0	-1.11	0	1.16	0
KQU62_13000	fadB	CcpA	0	0	0	-1.115	0.343	0.781	-0.424
KQU62_13005	fadD	CcpA	0	0	0	-1.861	0.527	0.672	1.68
KQU62_13010	fadE	CcpA	0	0	0	-1.063	0.789	2.73	3.19
KQU62_13015	fadX	CcpA	0	0	0	-1.286	0.699	0	0
KQU62_13025	prsS	0	0	0	0	-0.801	0.402	-1.43	0
KQU62_13030	opp-5A	0	0	0	0	-0.398	-0.555	1.69	0
KQU62_13035	-	0	0	0	0	-2.165	-0.902	0	0
KQU62_13040	-	0	0	0	0	-1.575	0	0	0
KQU62_13045	hmp	0	0	0	0	-1.712	-0.941	0.408	-0.585
KQU62_13055	ldh1/ lctE	Rex	0	0	0	-3.418	-0.977	0	-0.523
KQU62_13060	glcC	NanR	0	0	0	-2.18	-1.183	0	0
KQU62_13065	-	0	0	0	0	1.285	0.835	-1.3	0
KQU62_13075	bglG	0	0	0	0	-0.62	-1.522	0	0
KQU62_13110	tarI'	0	0	0	0	-1.349	-0.455	0	0
KQU62_13115	tarJ'	0	0	0	0	-1.276	-0.378	0	0
KQU62_13120	tarL'	0	0	0	0	-1	-0.399	0	0
KQU62_13125	tarF	0	0	0	0	2.15	0	0.308	0
KQU62_13145	tarS	0	0	0	0	1.041	-0.348	0.599	-0.252
KQU62_13150	scdA	0	0	0	0	-2.469	-1.214	0	-0.638

						Transcriptome (Log2 Fold Change)		Proteome (Log2 Fold Change)	
**Locus SG511**	**Gene Name**	**Regulator 1**	**Regulator 2**	**Regulator 3**	**Regulator 4**	**CmR-02**	**02REV**	**CmR-02**	**02REV**
KQU62_13155	lytS	0	0	0	0	-1.366	-0.169	0	0
KQU62_13165	lrgA	0	0	0	0	-2.055	-1.937	0	0
KQU62_13170	lrgB	0	0	0	0	-3.226	-2.297	0	0
KQU62_13175	-	0	0	0	0	-3.313	-1.859	0	0
KQU62_13195	ubiE	0	0	0	0	1.295	0.446	0	0
KQU62_13220	rbsR	SigB	0	0	0	-1.055	-0.469	0	0
KQU62_13230	mnmA	0	0	0	0	-1.973	-0.505	-1.11	0
KQU62_13235	-	0	0	0	0	3.85	0	0	0
KQU62_13240	-	0	0	0	0	1.33	0	0	0
KQU62_13245	lytM	WalR	0	0	0	3.158	-0.685	2.73	0
KQU62_13250	-	0	0	0	0	-1.567	-0.39	0	0
KQU62_13255	-	0	0	0	0	-1.588	-0.287	0	0
KQU62_13260	-	0	0	0	0	-1.341	0	0	0
KQU62_13265	-	0	0	0	0	-1.059	-0.189	-1.06	0
KQU62_13270	-	0	0	0	0	-1.315	0	0	0
KQU62_13275	esxA	0	0	0	0	-3.114	-0.727	0	0
KQU62_13345	-	0	0	0	0	-1.642	-0.36	0	0
KQU62_13350	-	0	0	0	0	-1.942	0	0	0
KQU62_13355	-	0	0	0	0	-2.049	-0.385	0	0
KQU62_13360	-	0	0	0	0	-1.717	0	0	0
KQU62_13365	-	0	0	0	0	-1.781	-0.62	0	0
KQU62_13370	-	0	0	0	0	-1.842	-0.695	0	0
KQU62_13375	-	0	0	0	0	-1.303	0	0	0
KQU62_13380	-	0	0	0	0	-1.483	-0.337	0	0
KQU62_13385	-	0	0	0	0	-1.549	0	0	0
KQU62_13395	-	0	0	0	0	-1.16	0	0	0
KQU62_13400	-	0	0	0	0	-2.413	0	0	0.943

						Transcriptome (Log2 Fold Change)		Proteome (Log2 Fold Change)	
**Locus SG511**	**Gene Name**	**Regulator 1**	**Regulator 2**	**Regulator 3**	**Regulator 4**	**CmR-02**	**02REV**	**CmR-02**	**02REV**
KQU62_13405	-	0	0	0	0	-2.144	0	0	0
KQU62_13415	-	0	0	0	0	-2.379	0	0	0
KQU62_13420	-	SigB	0	0	0	-1.172	-1.159	0	0

References

1. Urban A, Eckermann S, Fast B, Metzger S, Gehling M, Ziegelbauer K, Rübsamen-Waigmann H, Freiberg C. 2007. Novel whole-cell antibiotic biosensors for compound discovery. Appl Environ Microbiol 73:6436–6443. doi:10.1128/AEM.00586-07.
2. Biswas R, Voggu L, Simon UK, Hentschel P, Thumm G, Götz F. 2006. Activity of the major staphylococcal autolysin Atl. FEMS Microbiol Lett 259:260–268. doi:10.1111/j.1574-6968.2006.00281.x.
3. Kuroda M, Ohta T, Uchiyama I, Baba T, Yuzawa H, Kobayashi I, Cui L, Oguchi A, Aoki K-i, Nagai Y, Lian J, Ito T, Kanamori M, Matsumaru H, Maruyama A, Murakami H, Hosoyama A, Mizutani-Ui Y, Takahashi NK, Sawano T, Inoue R-i, Kaito C, Sekimizu K, Hirakawa H, Kuhara S, Goto S, Yabuzaki J, Kanehisa M, Yamashita A, Oshima K, Furuya K, Yoshino C, Shiba T, Hattori M, Ogasawara N, Hayashi H, Hiramatsu K. 2001. Whole genome sequencing of meticillin-resistant *Staphylococcus aureus*. The Lancet 357:1225–1240. doi:10.1016/S0140-6736(00)04403-2.
4. Novick R. 1967. Properties of a cryptic high-frequency transducing phage in *Staphylococcus aureus*. Virology 33:155–166. doi:10.1016/0042-6822(67)90105-5.
5. Frees D, Qazi SNA, Hill PJ, Ingmer H. 2003. Alternative roles of ClpX and ClpP in *Staphylococcus aureus* stress tolerance and virulence. Mol Microbiol 48:1565–1578. doi:10.1046/j.1365-2958.2003.03524.x.
6. Kreiswirth BN, Löfdahl S, Betley MJ, O'Reilly M, Schlievert PM, Bergdoll MS, Novick RP. 1983. The toxic shock syndrome exotoxin structural gene is not detectably transmitted by a prophage. Nature 305:709–712. doi:10.1038/305709a0.
7. Fey PD, Endres JL, Yajjala VK, Widhelm TJ, Boissy RJ, Bose JL, Bayles KW. 2013. A genetic resource for rapid and comprehensive phenotype screening of nonessential *Staphylococcus aureus* genes. mBio 4:e00537-12. doi:10.1128/mBio.00537-12.
8. Bæk KT, Gründling A, Mogensen RG, Thøgersen L, Petersen A, Paulander W, Frees D. 2014. β-Lactam resistance in methicillin-resistant *Staphylococcus aureus* USA300 is increased by inactivation of the ClpXP protease. Antimicrob Agents Chemother 58:4593–4603. doi:10.1128/AAC.02802-14.
9. Sahl H-G, Brandis H. 1982. Mode of Action of the Staphylococcin-like Peptide Pep 5 and Culture Conditions Effecting its Activity. Zentralbl Bakteriol Mikrobiol Hyg A 252:166–175. doi:10.1016/S0174-3031(82)80114-5.
10. Burkholder PR, Giles NH. 1947. Induced Biochemical Mutations in *Bacillus subtilis*. Am J Bot 34:345. doi:10.2307/2437147.
11. Spizizen J. 1958. Transformation of biochemically deficient strains of *Bacillus subtilis* by deoxyribonucleate. Proc Natl Acad Sci U S A 44:1072–1078. doi:10.1073/pnas.44.10.1072.
12. Pan Q, Garsin DA, Losick R. 2001. Self-Reinforcing Activation of a Cell-Specific Transcription Factor by Proteolysis of an Anti-σ Factor in *B. subtilis*. Mol Cell 8:873–883. doi:10.1016/S1097-2765(01)00362-8.
13. Msadek T, Dartois V, Kunst F, Herbaud ML, Denizot F, Rapoport G. 1998. ClpP of *Bacillus subtilis* is required for competence development, motility, degradative enzyme synthesis, growth at high temperature and sporulation. Mol Microbiol 27:899–914. doi:10.1046/j.1365-2958.1998.00735.x.
14. Gerth U, Krüger E, Derré I, Msadek T, Hecker M. 1998. Stress induction of the *Bacillus subtilis clpP* gene encoding a homologue of the proteolytic component of the Clp protease and the

involvement of ClpP and ClpX in stress tolerance. Mol Microbiol 28:787–802. doi:10.1046/j.1365-2958.1998.00840.x.

15. Piggot PJ. 1973. Mapping of asporogenous mutations of *Bacillus subtilis*: a minimum estimate of the number of sporeulation operons. J Bacteriol 114:1241–1253. doi:10.1128/jb.114.3.1241-1253.1973.
16. Perego M, Cole SP, Burbulys D, Trach K, Hoch JA. 1989. Characterization of the gene for a protein kinase which phosphorylates the sporulation-regulatory proteins Spo0A and Spo0F of *Bacillus subtilis*. J Bacteriol 171:6187–6196. doi:10.1128/jb.171.11.6187-6196.1989.
17. Nakano MM, Hajarizadeh F, Zhu Y, Zuber P. 2001. Loss-of-function mutations in *yjbD* result in ClpX- and ClpP-independent competence development of *Bacillus subtilis*. Mol Microbiol 42:383–394. doi:10.1046/j.1365-2958.2001.02639.x.
18. Leclercq R, Derlot E, Duval J, Courvalin P. 1988. Plasmid-mediated resistance to vancomycin and teicoplanin in *Enterococcus faecium*. N Engl J Med 319:157–161. doi:10.1056/NEJM198807213190307.
19. Kodali S, Galgoci A, Young K, Painter R, Silver LL, Herath KB, Singh SB, Cully D, Barrett JF, Schmatz D, Wang J. 2005. Determination of selectivity and efficacy of fatty acid synthesis inhibitors. J Biol Chem 280:1669–1677. doi:10.1074/jbc.M406848200.
20. Andersen KR, Leksa NC, Schwartz TU. 2013. Optimized *E. coli* expression strain LOBSTR eliminates common contaminants from His-tag purification. Proteins 81:1857–1861. doi:10.1002/prot.24364.
21. Miroux B, Walker JE. 1996. Over-production of proteins in *Escherichia coli*: mutant hosts that allow synthesis of some membrane proteins and globular proteins at high levels. J Mol Biol 260:289–298. doi:10.1006/jmbi.1996.0399.
22. Forsyth RA, Haselbeck RJ, Ohlsen KL, Yamamoto RT, Xu H, Trawick JD, Wall D, Wang L, Brown-Driver V, Froelich JM, C KG, King P, McCarthy M, Malone C, Misiner B, Robbins D, Tan Z, Zhu Zy Z-y, Carr G, Mosca DA, Zamudio C, Foulkes JG, Zyskind JW. 2002. A genome-wide strategy for the identification of essential genes in *Staphylococcus aureus*. Mol Microbiol 43:1387–1400. doi:10.1046/j.1365-2958.2002.02832.x.
23. Berscheid A. 2014. Characterization of *Staphylococcus aureus* VC40 reveals a major role of the VraS and WalK histidine kinases in the development of vancomycin and daptomycin resistance. Friedrich Wilhelms Universität, Bonn.
24. Türck M, Bierbaum G. 2012. Purification and activity testing of the full-length YycFGHI proteins of *Staphylococcus aureus*. PLoS ONE 7:e30403. doi:10.1371/journal.pone.0030403.
25. Fuchs S, Mehlan H, Bernhardt J, Hennig A, Michalik S, Surmann K, Pané-Farré J, Giese A, Weiss S, Backert L, Herbig A, Nieselt K, Hecker M, Völker U, Mäder U. 2018. AureoWiki‑The repository of the *Staphylococcus aureus* research and annotation community. Int J Med Microbiol 308:558–568. doi:10.1016/j.ijmm.2017.11.011.
